# Effectiveness of drug interventions to prevent delirium after surgery for older adults: systematic review and network meta-analysis of randomised controlled trials

**DOI:** 10.1136/bmj-2025-085539

**Published:** 2026-02-12

**Authors:** Matthew Luney, Luke Holdsworth, Arwa Hagana, Viktorija Kaminskaite, Lin Qiu, Juan Berner, Kyle Pattinson, Jagdeep Nanchahal, Gary S Collins, Matthew Costa

**Affiliations:** 1 Kadoorie Institute of Trauma, Emergency and Critical Care, Nuffield Department of Orthopaedics Rheumatology and Musculoskeletal Science, University of Oxford, Oxford, UK; 2 Nuffield Department of Anaesthesia, Oxford University Hospitals NHS Foundation Trust, Oxford, UK; 3 Department of Anaesthesia, Buckinghamshire Healthcare Trust, Aylesbury, UK; 4 Department of Otolarynology, Oxford University NHS Foundation Trust, Oxford, UK; 5 Nuffield Department of Clinical Neurosciences, University of Oxford, Oxford, UK; 6 Division of Plastic and Reconstructive Surgery, Department of Surgery, The University of Texas Health Science Center at San Antonio, TX, USA; 7 Kennedy Institute, Nuffield Department of Orthopaedics, Rheumatology and Musculoskeletal Science, University of Oxford; 8 Department of Applied Health Sciences, School of Health Sciences, College of Medicine and Health, University of Birmingham, Birmingham, UK; 9 NIHR Birmingham Biomedical Research Centre, University Hospitals Birmingham NHS Foundation Trust, Birmingham, UK

## Abstract

**Objective:**

To identify which drugs are effective at preventing delirium after surgery in adults over 60 years of age and estimate the effects on morbidity and mortality.

**Design:**

Systematic review and network meta-analysis.

**Data sources:**

Embase, Medline, and Cochrane Library up to 4 March 2024.

**Eligibility criteria:**

Randomised controlled trials with administration of one or more drugs for the prevention of delirium after surgery requiring general or regional anaesthesia that recruited participants at least 60 years old and used a validated delirium assessment tool to measure the outcome. Surgery under local anaesthesia only, preoperative mechanical ventilation, and studies of interventions to treat delirium were excluded.

**Data extraction and synthesis:**

Assessors masked to each other’s decisions screened studies, extracted data, and assessed risk of bias and quality of evidence in duplicate by using the Cochrane risk of bias tool version 2 and the CINeMA tool. Bayesian arm based network meta-analysis was used to compare interventions.

**Results:**

158 trials were identified with 41 084 participants comparing 52 drug interventions. Seventeen trials were rated as being at high risk of bias. The overall risk of delirium after surgery was 14.5% (n=5957). Dexmedetomidine (odds ratio 0.46, 95% credible interval 0.36 to 0.57), corticosteroids (0.53, 0.31 to 0.87), melatonin receptor agonists (0.54, 0.34 to 0.85), parecoxib (0.34, 0.16 to 0.74), olanzapine (0.27, 0.07 to 0.94), and intranasal insulin (0.13, 0.04 to 0.34) were the most effective interventions at preventing delirium in trials not at high risk of bias. Only corticosteroids reduced the severity of delirium (mean difference −2.42 (95% credible interval −4.72 to −0.12) Memorial Delirium Assessment Scale points). Most interventions had no effect on length of stay, mortality, cognition, or quality of life. Hypotension and bradycardia were more common with dexmedetomidine, but postoperative nausea and vomiting were reduced. Postoperative infection rates were not increased by corticosteroids.

**Conclusions:**

Dexmedetomidine is effective in the prevention of postoperative delirium. This finding remains after exclusion of studies at high risk of bias. Corticosteroids, melatonin receptor agonists, parecoxib, intranasal insulin, and olanzapine have potential benefit, although evidence is of moderate to very low quality. Evidence synthesis in this area is complicated by inadequate trial registration practices and incomplete adoption of core outcome sets.

**Systematic review registration:**

PROSPERO CRD42023488337.

## Introduction

Delirium is a common, serious, and costly condition that is a growing challenge for both patients and healthcare professionals in the face of an ageing population.^1^ ^2^ ^3^ ^4^ The condition is characterised by an acute fluctuating disturbance of attention and consciousness and is associated with a threefold increase in the risk of development of dementia over five years.^5^ ^6^ After major surgery, up to a quarter of older adults experience delirium.^7^ The increased economic costs attributable to postoperative delirium exceed $32bn (£24bn; €27bn) per year in the US.^8^

Uncertainty persists as to whether the prophylactic administration of drugs is an effective strategy to prevent delirium, with multiple national and international guidelines (including the UK’s National Institute for Health and Care Excellence) concluding that further evidence is needed to make practice recommendations.^9^ ^10^ ^11^ ^12^ ^13^ Previous evidence syntheses of drug prevention of delirium primarily focused on intensive care and general medical patients. However, the timing and nature of these patients’ presentation to hospital is highly unpredictable.^14^ ^15^ By contrast, the day of surgery is a clearly defined and opportune time for drug prophylaxis against delirium. With increased availability of more sensitive biomarkers of neuronal dysfunction, the evidence for the neuroinflammatory basis of the pathogenesis of delirium is improving.^16^ ^17^ ^18^ ^19^ ^20^ ^21^ Consequently, a better mechanistic rationale now exists to justify drug intervention to prevent delirium, particularly with drugs that down regulate pro-inflammatory pathways.

Doing a network meta-analysis allows clinicians and policy makers to compare clinical effectiveness across the range of drugs studied in delirium prevention trials and to assess the relative effectiveness between potential treatments that may not have been directly tested. This method intrinsically facilitates the inclusion of multi-arm trials and interventions using more than one drug. The findings also serve to guide researchers in identifying interventions to prioritise for further research and generate robust estimates of treatment effects between different drugs. Therefore, we did a systematic review and network meta-analysis to compare the effectiveness of drug interventions to prevent delirium in people aged 60 years or over undergoing surgery. The primary outcome was the incidence of delirium after surgery, with additional outcomes based on the core outcome set for delirium trials and outcomes important to our patient and public involvement group, including severity of delirium, quality of life, postoperative cognition, length of stay, mortality, and complications.^22^ ^23^

## Methods

The findings are reported according to the Preferred Reporting Items for Systematic Reviews and Meta-Analyses-Network Meta-analyses (PRISMA-NMA) guideline.^24^ The review was registered on PROSPERO (CRD42023488337) before searches were conducted.

### Eligibility

Eligible studies were randomised controlled trials that included participants aged 60 years or older who were undergoing surgery that required general or regional anaesthesia. The intervention was perioperative administration of a prophylactic drug to reduce postoperative delirium. A drug was defined as a prescription-only medication or other medication given by any route under the direction of a physician or other suitably qualified healthcare professional. Perioperative administration required that the intervention was delivered on the same day as the operation. The assessment of delirium required the use of a validated tool or a clinical diagnosis made explicitly using published criteria such as the *Diagnostic and Statistical Manual of Mental Disorders*. The comparator could be placebo, another active drug to prevent delirium, or standard care.

We excluded studies if the surgery was done under local anaesthesia, the intervention was administered to treat delirium, or the participants required mechanical ventilation before surgery. We excluded interventions with zero events across all arms of that comparison (similar to both arm zero event studies in pairwise meta-analyses), as they preclude a reliable estimation of treatment effect without excessive assumptions.

### Information sources

We searched Ovid Medline, Embase, and the Cochrane Central Register of Controlled Trials (CENTRAL) databases, from inception to 4 March 2024, without filters or language restriction. Full search strategies are reported in supplementary file 1 section 1.

### Study selection and data collection

Duplicate records were removed in Rayyan.^25^ Two reviewers (ML, AH) independently screened titles and abstracts to identify potentially relevant studies. We retrieved full text reports for all potential studies identified during the title and abstract screening. Two reviewers (ML and either VK or LQ) independently reviewed full text reports against review eligibility criteria. When the reviewers were uncertain whether to include a study at screening or full text review, they consulted a third reviewer (MC) masked to the decision of the other reviewers. Two authors (ML, LH) extracted data in duplicate and independently.

The data collected included the intervention drug name, dose, route, and duration of administration; the time of administration relative to time of operation; the number of participants with delirium by allocated intervention; and the delirium assessment tool(s) used. We also collected data on the type of anaesthetic used to explore whether this affected the results. General study data included age and sex of participants, type of operation, urgency (elective or emergency) and duration of surgery, number of participants screened and rate of loss to follow-up, year of publication, country (or countries) of recruitment, masking of participants and assessors, and trial registration details. To assess secondary outcomes, we collected data on the severity of delirium, length of admission, mortality, complication rates, quality of life, postoperative cognitive dysfunction, healthcare costs, and level of independence after discharge.

### Certainty of evidence and risk of bias

We used the Cochrane risk of bias tool version 2 for assessment of bias in individual studies.^26^ Assessments were made in duplicate (ML, LH), with reviewers masked to each other’s ratings and a third reviewer (MC) adjudicating conflicts. We used the Confidence in Network Meta-analysis (CINeMA) tool for assessment of the quality of evidence in keeping with the latest review guidance (Cochrane Comparing Multiple Interventions Methods Group).^27^ ^28^ The CINeMA framework has been specifically developed using GRADE (Grading of Recommendations Assessment, Development, and Evaluation) for implementation within network meta-analyses.^29^ Detailed implementation of the CINeMA methods is available in supplementary file 1 section 10.

### Synthesis

We used a bayesian random effects arm based network meta-analysis for the primary outcome of incidence of postoperative delirium as well as our pre-specified subgroups and secondary outcomes, including analysing emergency and elective operations separately. We also assessed whether the type of anaesthesia moderated the treatment effect for the primary outcome by comparing trials in which surgery was done under general anaesthesia versus trials with surgery under regional anaesthesia (predominantly spinal or epidural anaesthesia). We did post hoc subgroup analysis by multiple age groups at the request of a reviewer. We compared effect sizes for event outcomes with odds ratios and 95% credible intervals. For the continuous outcomes length of stay and peak delirium severity we used means and 95% credible intervals for the measure of treatment effect. Severity of delirium was measured on the Memorial Delirium Assessment Scale (MDAS) or converted.^30^ Where median and range were reported, we used standard methods from the Cochrane Handbook where possible to impute mean and standard deviation.^31^

### Model fitting and assessments

We did the bayesian random effects meta-analyses model fitting and consistency assessments in the R programming language by using the *multinma* package in R (version 4.2.1). We fitted random effects models by using objective prior distributions (supplementary file 1 table S1). We constructed arm based random effects models (using the *multinma* package) to model relative effects both within studies and between interventions, such that clustering of interventions with an individual study was included in the model fit as an additional term in the model specification. We used binomial and gaussian likelihoods for the binary and continuous outcome models, respectively. The prior distribution for treatment effects was a normal distribution, whereas a half normal distribution was used for the model heterogeneity priors and normal distribution for treatment effect priors.^32^ ^33^ We assessed inconsistency by comparing the posterior mean residual deviance and deviance information criterion between the consistency model (random effects model) and inconsistency model (unrelated mean effects model).^34^ We assessed loop inconsistency by node splitting,^35^ and we compared effect estimates from direct and indirect evidence for each intervention visualised in density plots. We qualitatively assessed studies for transitivity on the basis of the distribution of potential effect modifiers, including age, sex, timing and mode of administration of the intervention, duration of surgery, and type of anaesthesia.^32^ ^33^ ^36^ The statistical code used for analysis is available on the Open Science Framework (https://osf.io/a63d7/?view_only=59ee035ad2854313b282292246e194b4).

### Patient and public involvement

People from the UK Musculoskeletal Trauma Patient and Public Involvement Group with experience of major surgery formed an advisory group to discuss the objectives of this review during the design phase, including identifying outcomes that were important to them. They also contributed to the discussion on how to present the results and in the production of a plain language summary (supplementary file 1 section 2).

## Results

### Study selection

**Fig 1:**
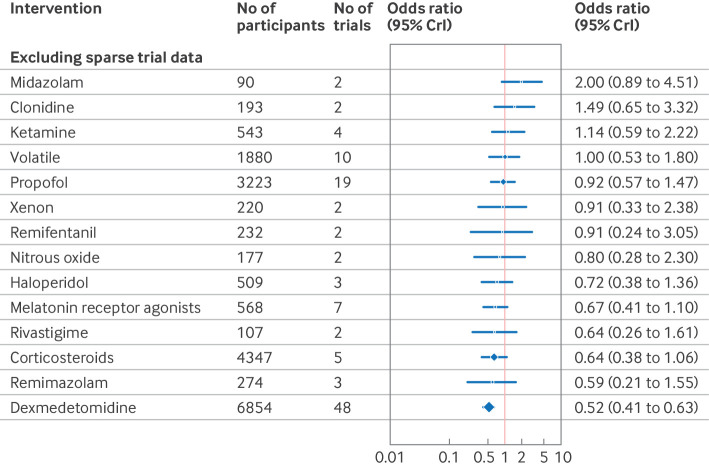
PRISMA flow diagram of study selection process

The searches of the electronic databases included studies up until 4 March 2024 and identified 9436 reports. Figure 1 shows the study selection process. After full text review, we included 158 randomised controlled trials consisting of 41 084 participants, 337 comparison arms, and 52 interventions and placebo. Four interventions consisted of a two drug combination; one intervention was a triple drug intervention. One hundred and twenty trials had an inactive control arm (placebo, n=113; usual care, n=7), and 38 trials involved two or more comparisons of active interventions. Unless otherwise specified, placebo refers to the inactive control arm including standard care. The median study size was 120 (interquartile range 80-259; range 16-7507) participants. Most were single centre trials (n=135), with 23 multicentre trials. The included studies were published between 1999 and 2024, with 55% (87/158) published between 2021 and 2024. Overall, the incidence of delirium after surgery was 14.5% (5957/41 084). The characteristics of included trials are listed in supplementary file 2 table S1, including event rates and postoperative intensive care use, along with full references.

### Network geometry

**Fig 2:**
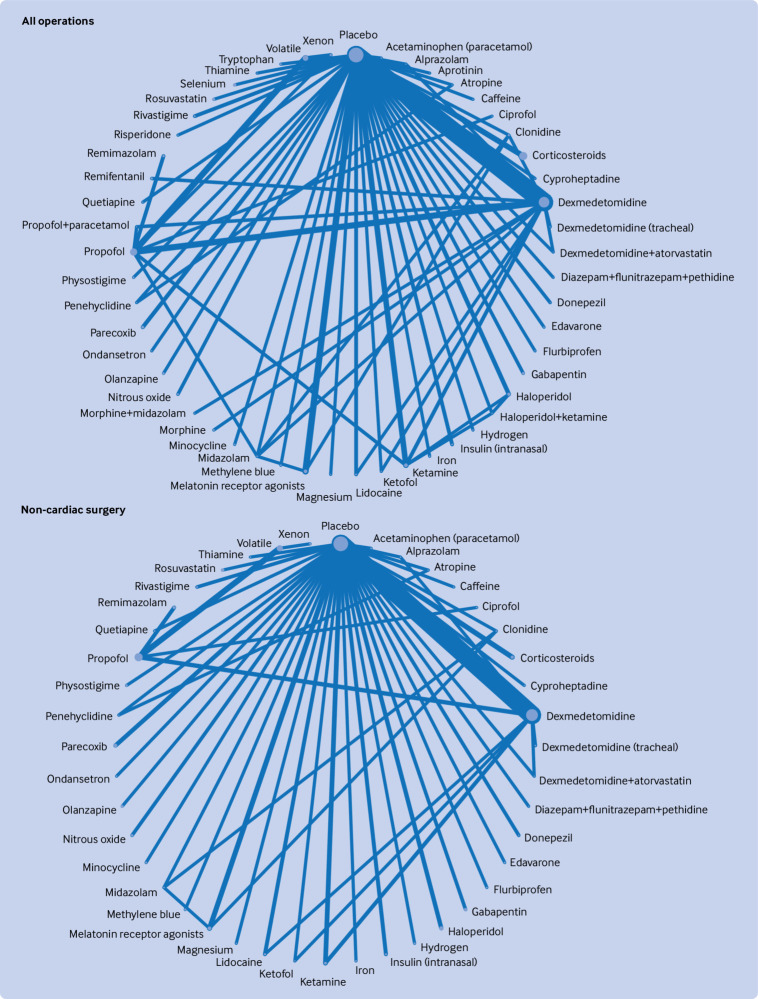
Network graphs of randomised controlled trials of drug prophylaxis against postoperative delirium depicting connectedness of networks for all operations and non-cardiac surgery. Node sizes correspond to number of participants in comparison arm; thickness of edges correspond to number of studies within that pair of direct comparisons

**Fig 3:**
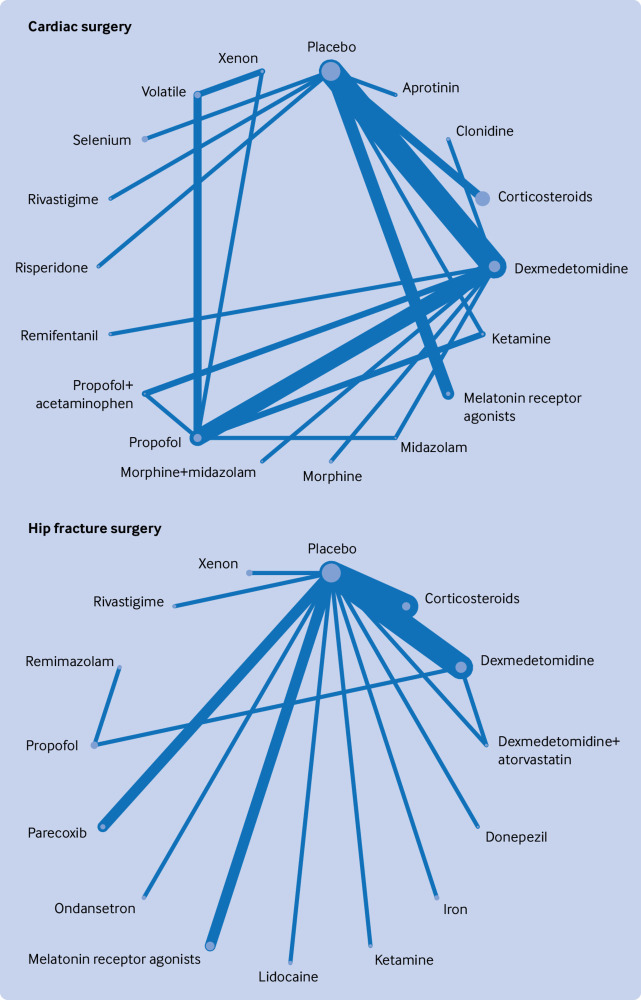
Network graphs of randomised controlled trials of drug prophylaxis against postoperative delirium depicting connectedness of networks for cardiac and hip fracture surgery. Node sizes correspond to number of participants in comparison arm; thickness of edges correspond to number of studies within that pair of direct comparisons

**Fig 4:**
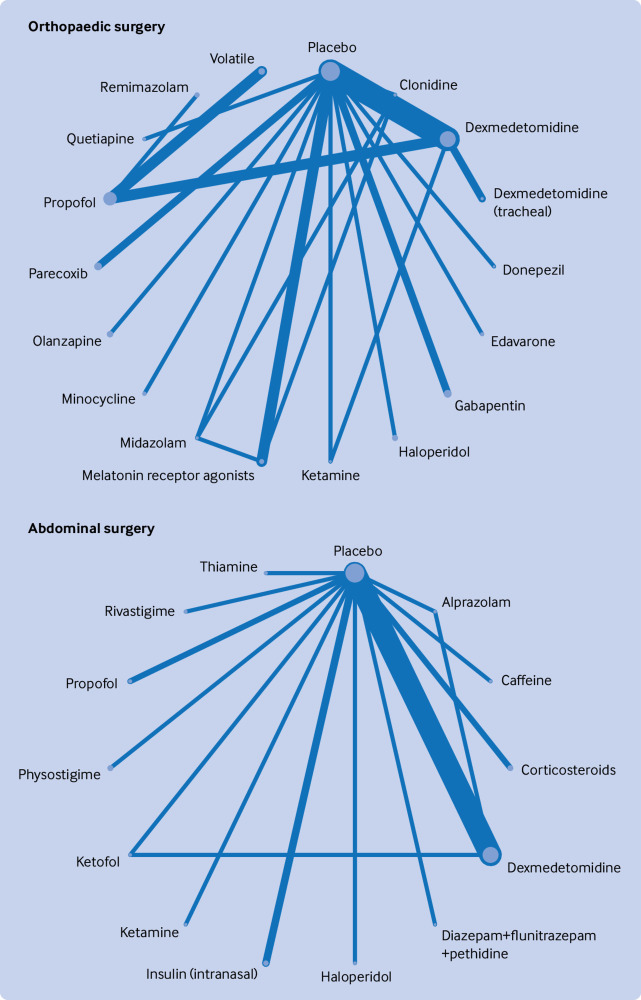
Network graphs of randomised controlled trials of drug prophylaxis against postoperative delirium depicting connectedness of networks for orthopaedic and abdominal surgery. Node sizes correspond to number of participants in comparison arm; thickness of edges correspond to number of studies within that pair of direct comparisons

**Fig 5:**
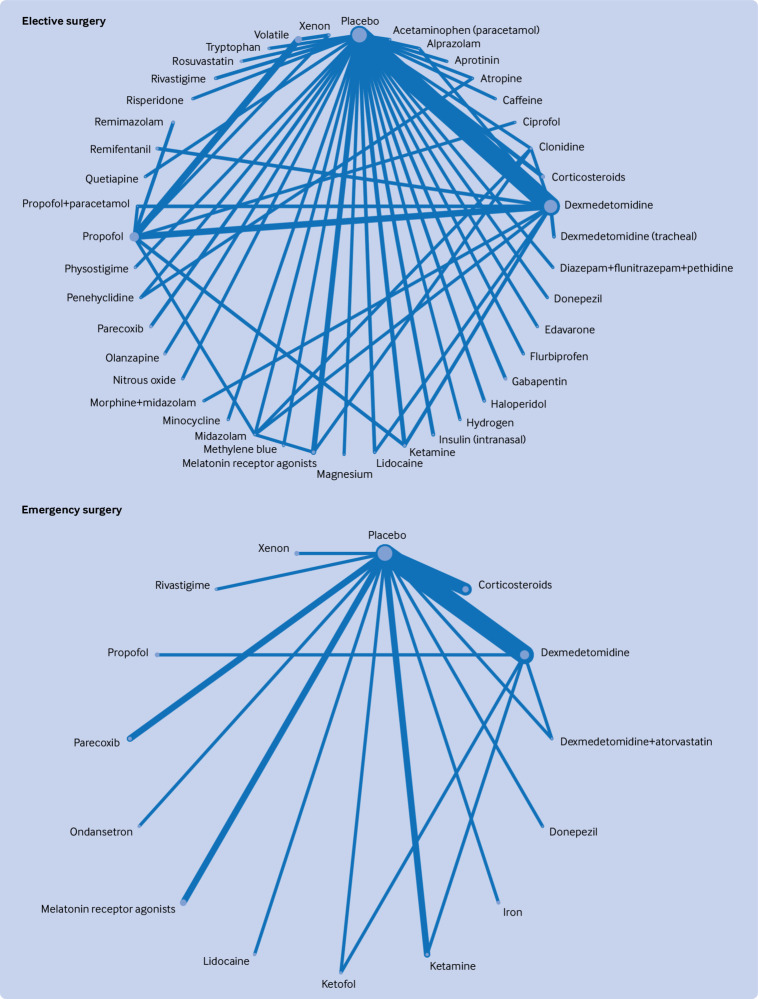
Network graphs of randomised controlled trials of drug prophylaxis against postoperative delirium depicting connectedness of networks by urgency of surgery. Node sizes correspond to number of participants in comparison arm; thickness of edges correspond to number of studies within that pair of direct comparisons

**Fig 6:**
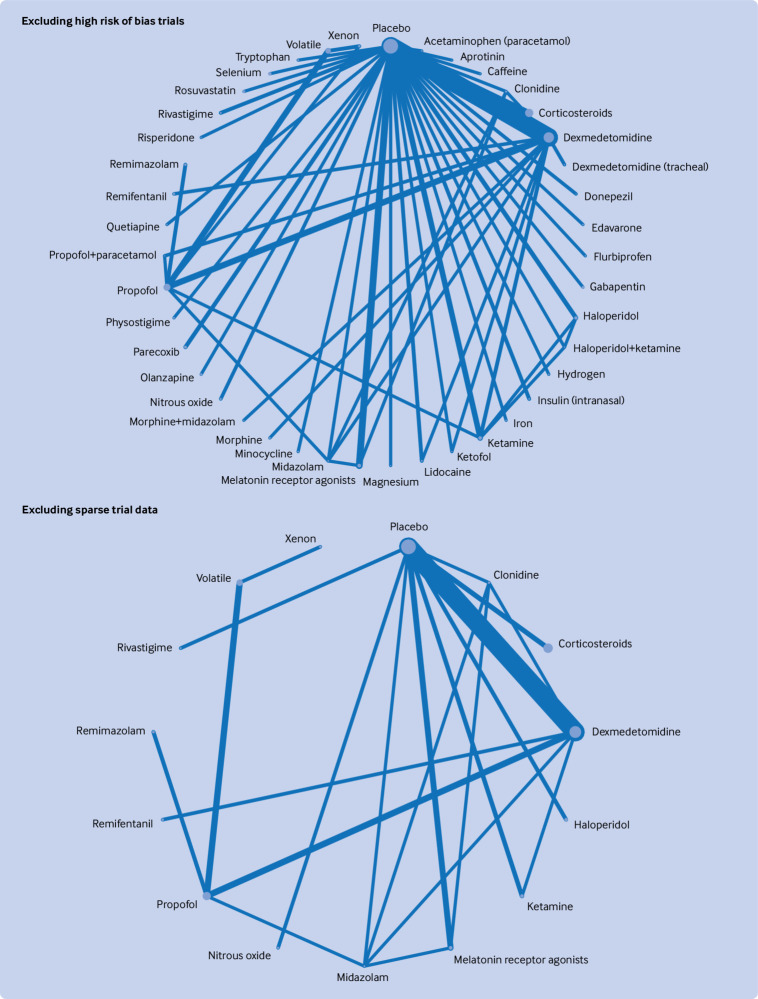
Network graphs of randomised controlled trials of drug prophylaxis against postoperative delirium depicting connectedness of networks excluding high risk of bias trials and sensitivity analysis excluding small/single trial interventions. Node sizes correspond to number of participants in comparison arm; thickness of edges correspond to number of studies within that pair of direct comparisons

Figure 2, figure 3, figure 4, figure 5, and figure 6 show the network plots with nodes representing the drugs, drug combinations, and placebo arms. Additional network plots for subgroups and secondary outcomes are reported in supplementary file 1 figures S5, S7, and S9. The assumption of the inconsistency model held for the global assessment (random effects model DIC 363.1 versus unrelated mean effects model 636.3, and posterior mean residual deviances 358.0 versus 355.9, respectively), in which a difference of >5 points in DIC would suggest inconsistency.^34^ We did not find evidence of loop inconsistency (P>0.0025, correcting for multiple comparisons) (supplementary file 1 section 8). We explored between study variation arising from different types of surgery and different types of anaesthesia in the pre-specified subgroups, with consistency in findings between groups further supporting the transitivity assumption holding.

### Risk of bias

**Fig 7:**
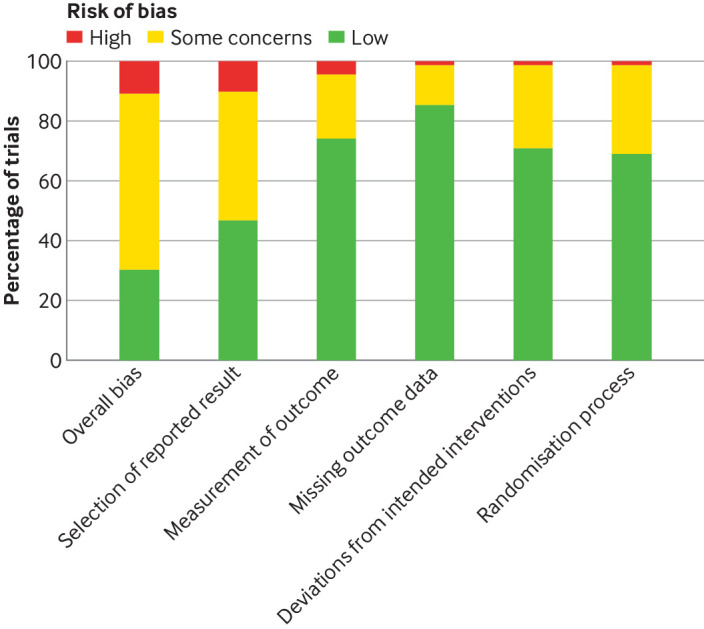
Summary plot of risk of bias assessments for included randomised controlled trials, using Cochrane Risk of Bias tool (version 2)

Overall, we rated 17 studies as being at high risk of bias and 48 studies had some concerns, predominantly selective reporting bias and poor trial registration practices (fig 7). The risk of bias for individual studies is available in supplementary file 3. Masking of participants and outcome assessors to group allocation was near universal. Masking to allocation for those administering the intervention was more variable (masked 58% (n=91/158), unmasked 28% (n=45/158), unreported 14% (n=22/158)). However, in all studies in which those administering the intervention were aware of the allocation, none of these staff participated in outcome assessment.

### Synthesis of results

**Fig 8:**
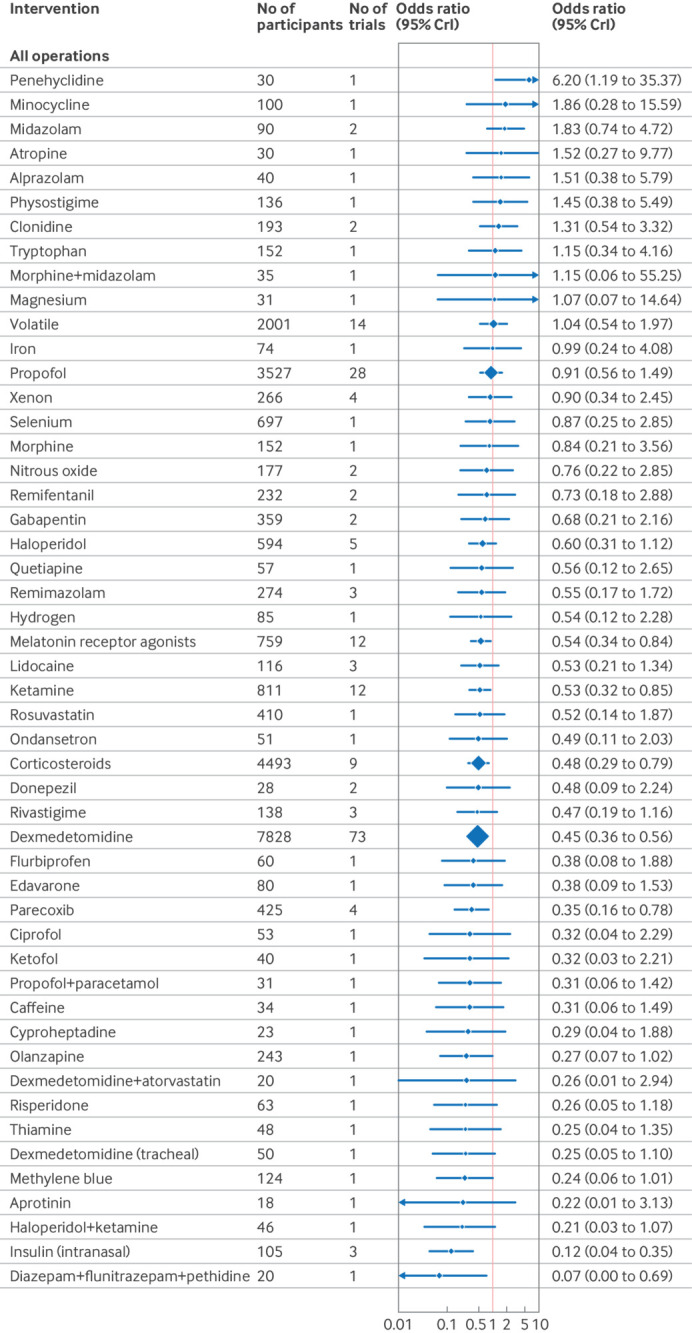
Forest plot of network meta-analysis results for randomised controlled trials of drugs to prevent delirium after surgery (all operations). Reference intervention is placebo. Box sizes correspond to number of participants. CrI=credible interval

**Fig 9:**
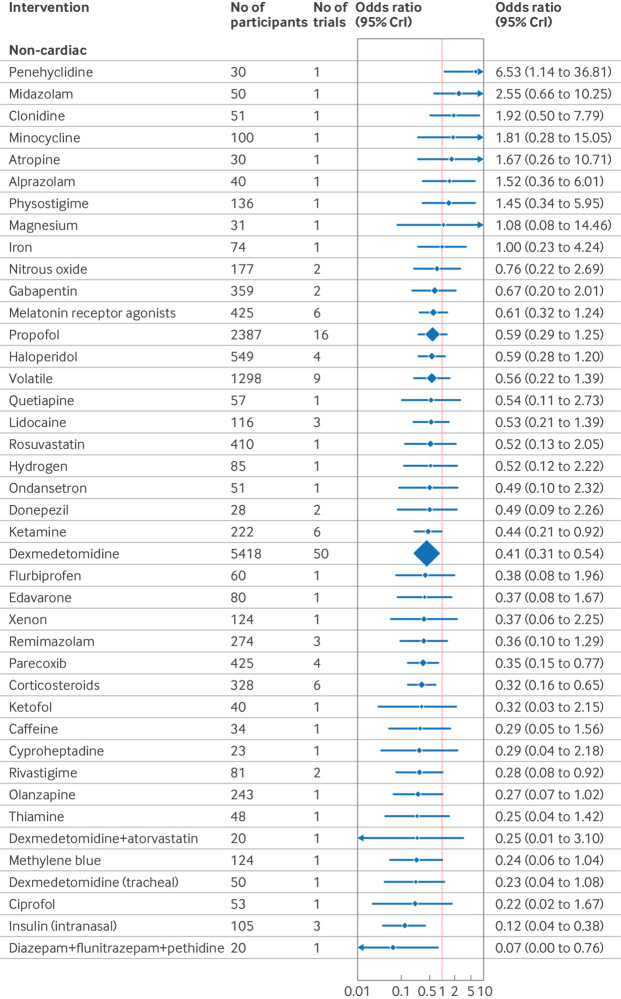
Forest plot of network meta-analysis results for randomised controlled trials of drugs to prevent delirium after non-cardiac surgery. Reference intervention is placebo. Box sizes correspond to number of participants. CrI=credible interval

**Fig 10:**
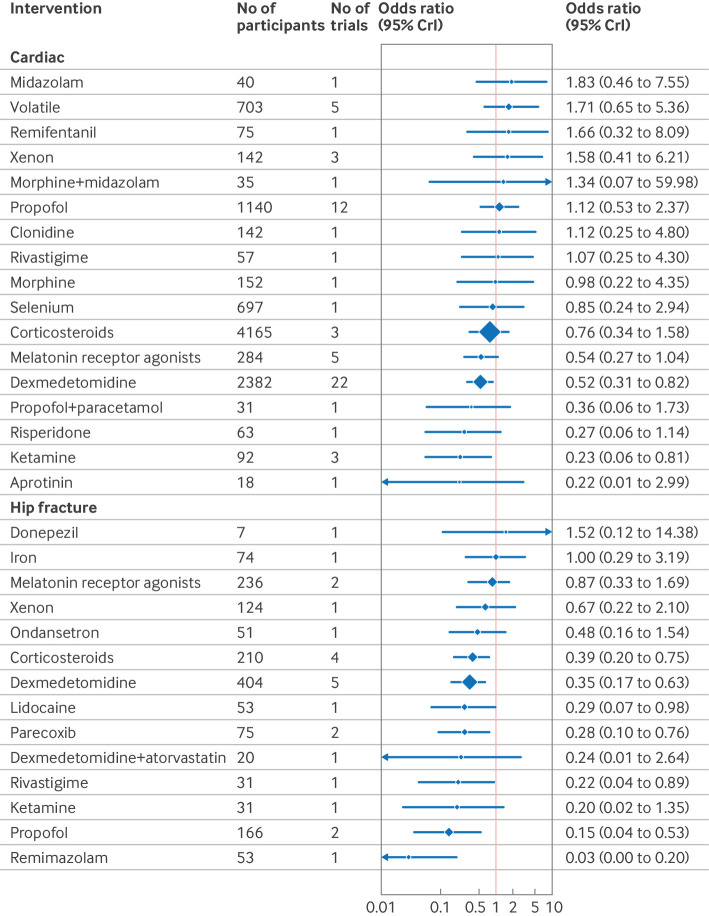
Forest plot of network meta-analysis results for randomised controlled trials of drugs to prevent delirium after cardiac and hip fracture surgery. Reference intervention is placebo. Box sizes correspond to number of participants. CrI=credible interval

**Fig 11:**
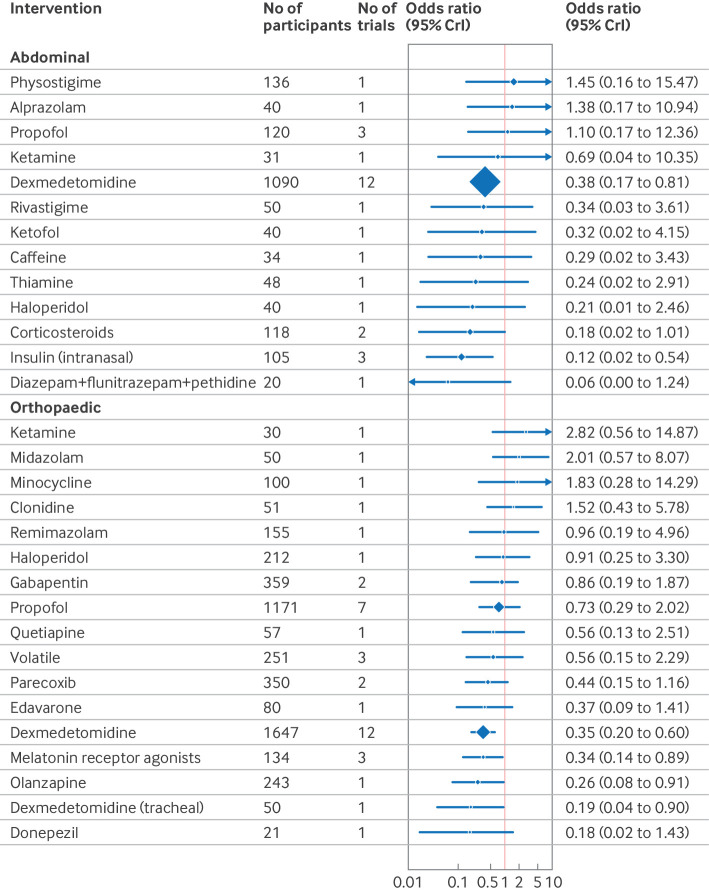
Forest plot of network meta-analysis results for randomised controlled trials of drugs to prevent delirium after orthopaedic and abdominal surgery. Reference intervention is placebo. Box sizes correspond to number of participants. CrI=credible interval

**Fig 12:**
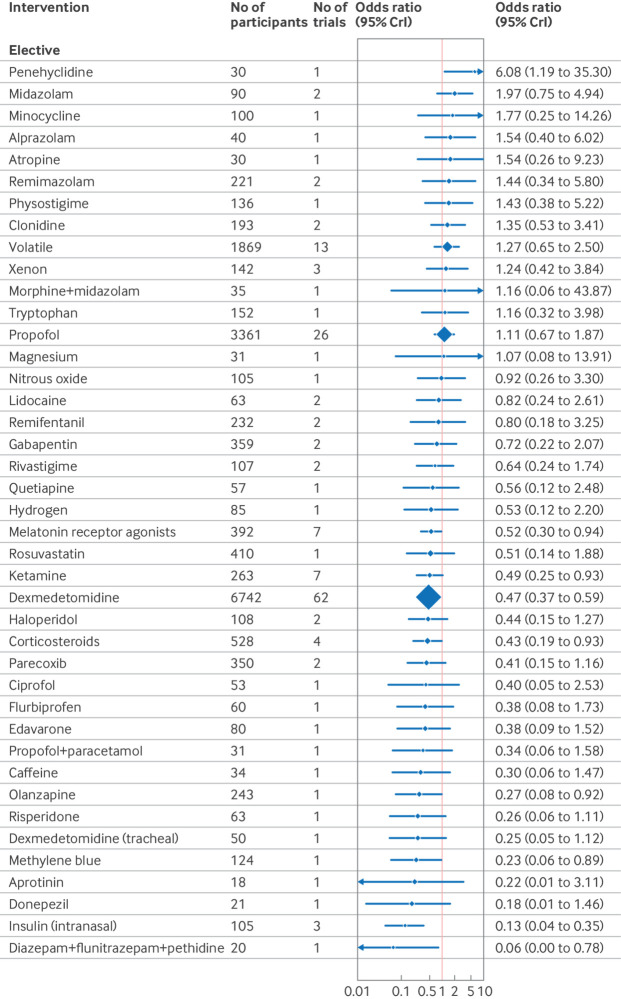
Forest plot of network meta-analysis results for randomised controlled trials of drugs to prevent delirium after elective surgery. Reference intervention is placebo. Box sizes correspond to number of participants. CrI=credible interval

**Fig 13:**
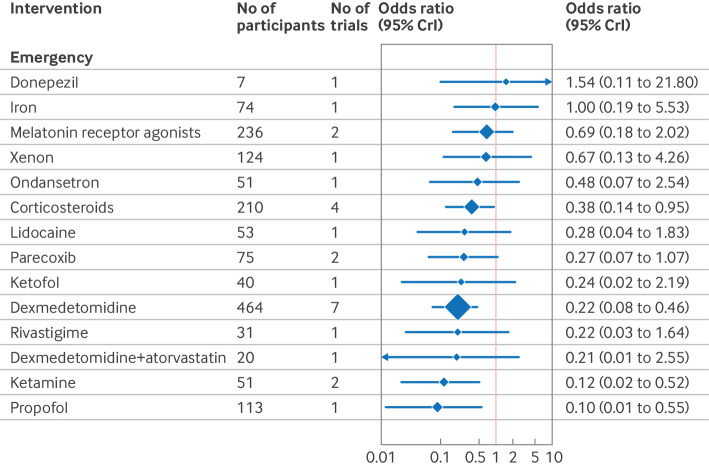
Forest plot of network meta-analysis results for randomised controlled trials of drugs to prevent delirium after emergency surgery. Reference intervention is placebo. Box sizes correspond to number of participants. CrI=credible interval

The confusion assessment method (CAM) including variants (CAM-ICU and 3D-CAM), were the most used delirium assessment tools (82% of included studies; n=130/158). Forest plots shows the effect estimates of drug interventions to prevent postoperative delirium for the global model (all surgical specialties) (fig 8), pre-specified individual specialties (subgroup analysis) (fig 9, fig 10, fig 11), and the estimated treatment effects for trials in elective (fig 12) and emergency settings (fig 13) separately. Additional forest plots by specialty and secondary outcomes are reported in supplementary file 1 sections 5 and 12 and supplementary file 4. Dexmedetomidine was consistently found to be effective in prevention of delirium (odds ratio 0.45, 95% credible interval (CrI) 0.36 to 0.56; n=7828; studies=73) in the global model, across most surgical specialties (not thoracic surgery) and in both elective (odds ratio 0.47, 0.37 to 0.59; n=6742; studies=62) and emergency settings (odds ratio 0.22, 0.08 to 0.46; n=464; studies=7).

Corticosteroids were effective across many settings (odds ratio 0.48, 95% CrI 0.29 to 0.79; n=4493; studies=9; global model), including the pre-specified subgroups of elective surgery, emergency surgery, hip fracture, and other major non-cardiac operations. Unlike dexmedetomidine, corticosteroids were not effective at preventing delirium after cardiac surgery (odds ratio 0.76, 0.34 to 1.58; n=4165; studies=3). Melatonin receptor agonists (odds ratio 0.34, 0.14 to 0.89) and olanzapine (0.26, 0.08 to 0.91) were effective in elective orthopaedic surgery. Intranasal insulin was effective for elective abdominal surgery (odds ratio 0.12, 0.02 to 0.54), and parecoxib was effective for hip fracture (0.28, 0.10 to 0.76) and other major non-cardiac surgery (0.35, 0.15 to 0.77).

We found potential effectiveness for some interventions in specific subgroup analyses; however, none of these was effective when trials at high risk of bias were excluded. These interventions were ketamine (elective and emergency surgery, cardiac and non-cardiac surgery); lidocaine, propofol, rivastigmine, and remimazolam (hip fracture repair); methylene blue (elective surgery); diazepam+flunitrazepam+pethidine (elective abdominal surgery); and intratracheal dexmedetomidine (elective orthopaedic surgery).

We found no interventions to be effective in preventing delirium following thoracic surgery. Penehyclidine was the only intervention to perform significantly worse than placebo (odds ratio 6.2, 95% CrI 1.19 to 35.37).

### Credibility assessment

Confidence in the effectiveness of drug prophylaxis, assessed using the CINeMA implementation of the GRADE framework, was moderate to very low (supplementary file 1 section 10). This was predominantly driven by within study bias arising from concerns about selective reporting in trials without registration. Publication bias was difficult to assess as few comparisons were studied in 10 or more trials (as quantitative methods for assessing publication bias with fewer studies are unreliable).^37^ The magnitude and direction of effect were consistent across many interventions, with heterogeneity generally being low. The interventions with no concerns for imprecision were dexmedetomidine, corticosteroids, insulin, ketamine, olanzapine, parecoxib, penehyclidine, methylene blue, and melatonin receptor agonists. No studies had major concerns for incoherence. Two trials were downgraded owing to poor masking of outcome assessors to the allocated interventions: methylene blue and diazepam+flunitrazepam+pethidine.^38^ ^39^

### Sensitivity analysis

**Fig 14:**
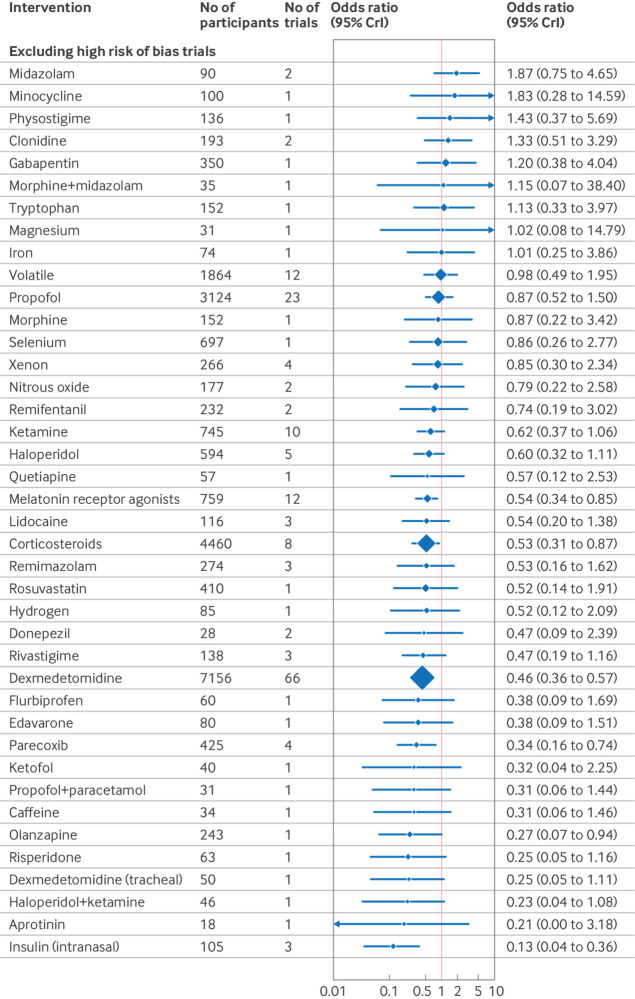
Sensitivity analysis forest plot of network meta-analysis results for randomised controlled trials of drugs to prevent delirium after surgery, excluding trials at high risk of bias. Reference intervention is placebo. Box sizes correspond to number of participants. CrI=credible interval

**Fig 15:**
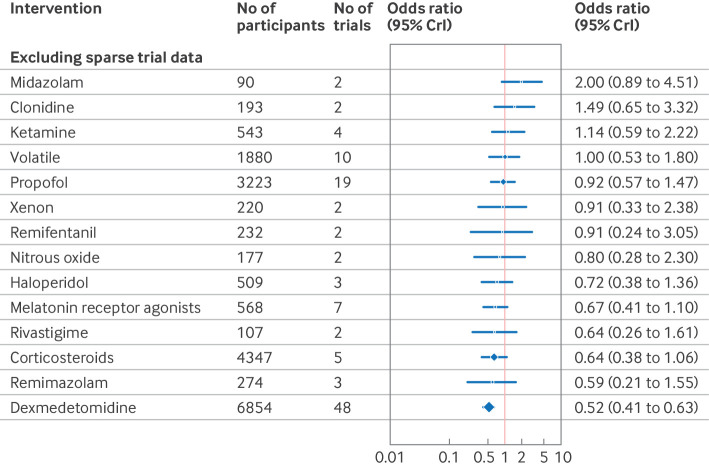
Sensitivity analysis forest plot of network meta-analysis results for randomised controlled trials of drugs to prevent delirium after surgery, excluding comparisons with <100 participants or studied in only one trial. Reference intervention is placebo. Box sizes correspond to number of participants. CrI=credible interval

In sensitivity analysis excluding studies at high risk of bias, dexmedetomidine (odds ratio 0.46, 95% CrI 0.36 to 0.57) and corticosteroids (0.53, 0.31 to 0.87) remained effective (fig 14). The surgical specialty specific findings for melatonin receptor agonists, parecoxib, olanzapine, and intranasal insulin also remained effective. Ketamine and combination diazepam+flunitrazepam+pethidine were not shown to be effective in this sensitivity analysis. To account for potential bias from small study effects, analysis excluding intervention studied in only one trial and trials of fewer than 100 participants showed efficacy for dexmedetomidine (odds ratio 0.52, 0.41 to 0.63), as shown in figure 15.

### Effect modification

We studied whether the results of our primary analysis were affected by the main type of anaesthesia the participants received for their surgery, either general or regional (including spinal) anaesthesia. Data on anaesthesia type were available for 95% (150/158) of trials. Overall, the type of anaesthesia used (general or regional) was not an important moderator of the treatment effect estimates. No interventions showed important between group differences in magnitude or direction of treatment effect between general anaesthesia and regional anaesthesia groups, although assessing all interventions was not possible. For instance, insulin and lidocaine were studied only in trials using general anaesthesia. Dexmedetomidine was effective irrespective of anaesthesia group: odds ratio 0.45 (95% CrI 0.35 to 0.57; n=6050; studies=62) for general anaesthesia and 0.28 (0.11 to 0.64; n=1306; studies=9) for regional anaesthesia trials (supplementary file 1 figures S6 and S8). Steroids were studied in all three subgroups; however, efficacy over placebo was not demonstrated in these subgroups. The mean age of participants was over 80 years in 6.3% (n=10) of trials. Among very old participants (mean trial age >80 years), the odds ratio for the effectiveness of dexmedetomidine was 0.34 (0.02 to 2.44; n=213; studies=2) and that for corticosteroids was 0.36 (0.08 to 1.06; n=150; studies=3) (supplementary file 1 figure S14).

### Secondary outcomes

We also analysed the effectiveness for each intervention on severity of delirium, length of hospital admission, mortality, complication rates, quality of life, and postoperative cognitive dysfunction. Insufficient data were reported on level of independence after hospital discharge to allow synthesis of evidence. Similarly, reporting of health economics assessments for any of the interventions studied was very limited. Although we recognise that economic evaluations are sometimes reported separately from clinical results, the trial registration entries did not imply that the data were available elsewhere.

We found a modest reduction in severity of delirium with corticosteroids: mean difference −2.42 (95% CrI −4.72 to −0.12) MDAS points compared with placebo. No other interventions reduced the severity of delirium compared with placebo. Bradycardia (odds ratio 1.60, 95% CrI 1.32 to 2.00) and hypotension (1.40, 1.08 to 1.81) were increased with dexmedetomidine, but postoperative nausea and vomiting was decreased (0.67, 0.49 to 0.87). Infection related complications were not increased by steroids (odds ratio 0.97, 0.66 to 1.65), and no evidence of local anaesthetic systemic toxicity was reported for lidocaine (zero events). The remaining secondary outcomes are reported in supplementary file 1 section 12.

## Discussion

In this systematic review and network meta-analysis, we found dexmedetomidine to be effective at preventing delirium. This finding was consistent across a broad range of pre-specified surgical specialties and emergency and elective surgery; it remained after removal of trials at high risk of bias and after we accounted for small study effects. Dexmedetomidine was also effective irrespective of whether the main type of anaesthesia used was general or regional anaesthesia. Drugs that showed effectiveness in specific settings included corticosteroids, melatonin receptor agonists, parecoxib, insulin, and olanzapine. The evidence for effectiveness of other interventions was less certain owing to trials at high risk of bias. The benefits for insulin, olanzapine, and melatonin receptor agonists were demonstrated in specific elective, non-cardiac surgical subgroups, and those for parecoxib were demonstrated for hip fracture surgery. Secondary outcome measures were not improved by drug prophylaxis against delirium except for a reduction in the severity of delirium with corticosteroids. The interventions studied were not associated with a significant increase in serious complications.

### Comparison with existing literature

Our review is the largest study of the evidence for drug interventions to prevent delirium after surgery, in terms of the number of participants and drugs assessed and the application of the core outcome set for delirium trials.^22^ Previous meta-analyses and network meta-analyses have been conducted with more limited search strategies as evidenced by the small sample sizes,^14^ ^40^ ^41^ ^42^ ^43^ included multiple non-masked randomised trials,^40^ ^42^ or included studies of participants not undergoing surgery.^42^

We found dexmedetomidine to be effective across the spectrum of surgical specialties, and in both elective and emergency surgical settings. This aligns with previous reviews focused specifically on dexmedetomidine.^12^ ^44^ ^45^ ^46^ However, our analysis adds to the existing data by including surgical specialty specific analyses, and crucially our analyses allow comparison with other active interventions. We were also able to show that although dexmedetomidine reduced the incidence of delirium, this did not translate into other perioperative benefits; we found no reductions in mortality, length of stay, or severity of delirium.

A substantial body of evidence shows that neuroinflammation is important in the development of postoperative delirium.^11^ ^20^ ^47^ This also explains the increased vulnerability to delirium seen with ageing and in people with existing cognitive impairment.^48^ ^49^ People with an already chronically disrupted or poorly functioning blood-brain barrier will have less reserve to cope with the pro-inflammatory state arising from a surgical insult, especially complex (long) and emergency operations.^50^ ^51^ ^52^ Many of the drugs we found to be effective in preventing delirium after surgery have anti-inflammatory properties: dexmedetomidine, corticosteroids, ketamine, lidocaine, and parecoxib.^53^ ^54^ ^55^ ^56^ ^57^ ^58^ Corticosteroids were also the only intervention to reduce the severity of delirium. These interventions also all have analgesic properties, offering a plausible opioid sparing mechanism of action too.^59^ ^60^ ^61^ ^62^

Melatonin receptor agonists act differently, promoting sleep via the suprachiasmatic nucleus of the hypothalamus.^63^ The benefit of circadian rhythm promotion in this context is consistent with evidence that natural sleep is disrupted in the postoperative period and with delirium.^20^ ^64^ However, similar research in prevention of delirium in the intensive care setting did not find effectiveness.^65^ The benefit in orthopaedic surgery, particularly under regional anaesthesia rather than general anaesthesia, needs further study as this finding may be confounded by patients’ comorbidities influencing the choice of anaesthetic in orthopaedic surgery.

Olanzapine, an atypical antipsychotic with dopamine antagonism effects, was found to be effective in one trial of elective non-cardiac surgery.^66^ Across five trials, we found no benefit to using haloperidol to prevent delirium. This is in agreement with previous work that antipsychotics do not have a role in delirium prevention and refutes the suggestion made in recent but much smaller reviews that haloperidol may be effective.^14^ ^42^ ^67^ ^68^ Further trials would be needed before antipsychotic use could be recommended for prevention of delirium.

Intranasal insulin acts to improve intracerebral metabolism and has been shown to improve performance on cognitive tasks.^69^ In contrast to intravenous and subcutaneous insulin, it does not affect blood glucose homoeostasis.^70^ The potential role for intranasal insulin for the prevention of delirium fits the hypothesis that impaired cerebral metabolism plays a crucial role in the development of delirium.^20^ ^71^ Related work investigating intranasal insulin as a potential treatment for delirium is ongoing.^72^

Our findings challenge the narrative that evidence for drug prevention of delirium is lacking,^73^ providing evidence of effectiveness for dexmedetomidine, corticosteroids, parecoxib, melatonin receptor agonists, insulin, and olanzapine. However, they are in agreement with previous reviews that the overall quality of evidence remains generally low, driven predominantly by reporting bias from poor trial registration practices or some cases no registration at all.^14^ ^41^

Considering the effect of anaesthesia, we found that the magnitude and direction of the effectiveness of interventions were consistent with the primary analyses for dexmedetomidine irrespective of anaesthetic type, suggesting mechanisms independent from anaesthesia. This is consistent with recent evidence from large trials showing that the choice of anaesthetic does not affect the incidence of delirium.^74^ ^75^

### Strengths, limitations, and generalisability

Our comprehensive search terms identified a large body of literature from which to appraise evidence for potentially effective interventions. When assessing the transitivity and consistency, we found that our models held, both for the global model of delirium prevention among all operations studied and for our pre-specified subgroups of individual surgical specialties and by urgency of surgery. To mitigate against potential intransitivity, we clearly defined our study population to be randomised controlled trials recruiting adults aged over 60 years, who on the day of surgery received one or more drugs as prophylaxis against delirium, and we mandated use of a validated tool for the outcome assessment. As is common to all network meta-analyses of drug interventions, we cannot exclude the possibility of co-intervention use. Given the size and geometry of the networks, the probability of this affecting the results is very low. The network geometry for the comparisons did not show concerning patterns such as avoidance of specific comparisons or problems with respect to connectedness. The large number of trials identified in our review has allowed us to appraise effectiveness across a wide range of surgical specialties and thus show a consistent treatment effect across different settings, particularly for dexmedetomidine.

Although heterogeneity is inevitable in an evidence synthesis of this size, statistical heterogeneity for between study variance and comparison between the credible intervals and prediction intervals for treatment effect did not show high levels of heterogeneity. Most trials administered interventions intravenously during or immediately after surgery, minimising potential variation arising from this. We cannot exclude the potential for heterogeneity in the estimation of treatment effect arising from variation in sensitivity of delirium assessment tools or differing incidence of polypharmacy between studies.^76^ However, most trials used the Confusion Assessment Method, which has been shown to have high inter-rater reliability, and the exact relation between polypharmacy and delirium prevention remains contentious.^77^ ^78^

We identified 17 trials with high risk of bias, mainly driven by poor trial reporting practices such as failure to register the trial or pre-specify an analysis plan. Reassuringly, our findings for dexmedetomidine, corticosteroids, melatonin receptor agonists, olanzapine, and intranasal insulin were not sensitive to exclusion of high trials with risk of bias, although this did affect the evidence for ketamine, lidocaine, rivastigmine, methylene blue, remimazolam, and diazepam+flunitrazepam+pethidine. Reporting (publication) bias was more difficult to detect owing to the small number of trials for certain interventions, and this risk of bias was accounted for in the CINeMA rating. High risk of bias commonly resulted in the quality of evidence CINeMA rating being low to very low.

Interpretation of the secondary outcomes was limited by a lack of consistency in measures used between trials, particularly for quality of life and longer term cognitive dysfunction. We identified five different tools used to assess severity of delirium, six tools for health related quality of life, and at least eight tools for postoperative cognitive dysfunction (not including different combinations of subcomponents of assessment batteries). Work has been done to harmonise nomenclature, but no consensus yet exists on the most appropriate assessment tools.^79^ Consistency in tools to measure the presence of delirium was greater, with the Confusion Assessment Method (or CAM-ICU) used by most studies. Despite these challenges, the large number of identified studies meant that we were still able to analyse most of our pre-specified secondary outcomes, thus showing the safety of the effective interventions and limitations of their benefits (lack of effect on length of stay, delirium severity, and mortality).

Our review excluded studies of delirium prevention for participants under 60 years of age. Delirium is considerably less common in younger than in older adults,^80^ and including these data would have impaired our ability to detect important findings in the population most at risk. Caution should be exercised when generalising these findings to delirium prevention outside of older adult surgical settings. Common to many perioperative randomised controlled trials, we found that adults over 80 years of age were underrepresented, which limited the data on the effectiveness of interventions in the oldest surgical patients.^81^

Our secondary outcomes also provide reassurance regarding safety and tolerability of the interventions. The complication rates in intervention arms were comparable to those for placebo. Consistent with previous work, the only complications of relevance that were increased with intervention were intraoperative bradycardia and hypotension with dexmedetomidine.^44^ However, this was not associated with any increase in renal or cardiac complications, suggesting that these effects were not severe.^12^ We also found an additional benefit of reduced postoperative nausea and vomiting compared with placebo. Data on emotional distress and institutional care needs after discharge were scarce.

An obvious gap in the literature exists with regard to the economic evaluation of interventions to reduce delirium. In the context of the overall costs of the provision of health and social care for surgical patients, none of the interventions studied is expensive.^82^ The cost of some interventions such as corticosteroids is negligible, and Djaiani and colleagues have shown that the cost of perioperative use of more recently developed drugs such as dexmedetomidine is also low.^83^ ^84^ However, formal evaluations of cost effectiveness are lacking.

We also provide a comprehensive estimate of the incidence of postoperative delirium (14.5%), from a large population of surgical patients (n=41 084), consistent with previous epidemiological work.^85^ The strength of this estimate is that the frequency of delirium assessment in the trial setting is generally more robust, typically at least twice daily, in contrast to routine clinical practice of once daily or less.^11^

### Implications for clinical practice, policy, and research

Cognitive impairment ranks among older patients’ greatest concerns for recovery after surgery,^1^ especially after emergency and trauma surgery.^3^ Despite this priority, most patients do not receive any drug intervention to prevent delirium owing to current multinational clinical guidance and older systematic reviews citing lack of efficacy.^10^ ^11^ ^14^ ^86^ Openness to using drug prophylaxis is increasing in North America, although clinical guidance stops short of recommending specific interventions.^12^

Our findings support revision of existing clinical guidance to recommend judicious use of dexmedetomidine perioperatively to prevent postoperative delirium in older adults requiring surgery irrespective of surgical urgency or type of anaesthesia, except thoracic surgery for which benefit was not demonstrated.^10^ ^11^ ^12^ The benefits of a reduced incidence of delirium should be weighed against the increased risk of bradycardia and hypotension. However, no increase in renal, cardiac, or pulmonary complications was seen; furthermore, postoperative nausea and vomiting were also reduced by dexmedetomidine.

For patients, our review provides information on the potential benefits of drug interventions (dexmedetomidine, corticosteroids, melatonin receptor agonists, parecoxib, intranasal insulin, and olanzapine) to reduce the risk of developing postoperative delirium. We also describe the limits to these benefits (little to no effect on severity of delirium, length of stay, mortality, or postoperative complications). Shared decision making conversations held before surgery between clinicians, patients, and carers remain paramount, encompassing individual patients’ preferences and current health as well as this latest evidence.^87^ ^88^ ^89^

Our review identified areas for potential improvement in the design and conduct of trials testing interventions to reduce postoperative delirium. Data are severely lacking on the impact these interventions have on the rate of discharge to institutional care, time to return to original residence, and cost effectiveness. We also found that emotional burden is rarely reported despite this being highly valued by patients.^22^ Although some trials were conducted before the publication of the core outcome set for delirium trials, our findings show that subsequent uptake remains low.^22^

A clear need exists for rigorously conducted trials to appraise the effects of these interventions on severity of delirium, emotional distress, quality of life, cognitive function, level of independence, and resource use to inform health economic analysis. A need also exists for increased harmonisation of delirium trial outcomes for more effective evidence appraisal.^90^

### Conclusion

In our network meta-analysis of randomised controlled trials, dexmedetomidine was found to be clinically effective for preventing delirium after surgery in older adults. This finding was consistent across elective and emergency surgery and almost every surgical specialty. Corticosteroids, melatonin receptor agonists, parecoxib, insulin, and olanzapine were effective in specific subgroup settings. However, more rigorously conducted, randomised studies are needed to reduce the frequent high risk of bias in the existing literature.

## What is already known on this topic

Consensus is lacking in multinational guidance regarding the use of drug interventions to prevent delirium
Whether any drugs are effective for preventing delirium after surgery is uncertain
Dexmedetomidine is the most likely intervention to be effective, with previous reviews focused on use in specific settings such as cardiac surgery and compared only with placebo

## What this study adds

Moderate certainty evidence indicates that dexmedetomidine is effective at preventing delirium across surgical specialties and urgency of surgery and irrespective of type of anaesthesia
Corticosteroids, melatonin receptor agonists, insulin, parecoxib, and olanzapine are potentially effective, but more studies are needed to overcome low quality evidence
Reporting of emotional distress and data availability for health economic analysis are very poor among delirium prevention trials

## Data Availability

Analysis code available on OSF (https://osf.io/a63d7/?view_only=59ee035ad2854313b282292246e194b4)

## Web extra

Extra material supplied by authors

Web appendix: Supplementary file 1

Web appendix: Supplementary file 2

Web appendix: Supplementary file 3

Web appendix: Supplementary file 4

Web appendix: Supplementary file 5

## Notes

Contributors: ML, JB, JN, and MC conceived the study. ML, JB, JN, GSC, and MC contributed to the protocol and methods for the study. ML, JB, JN, and MC designed the search strategy. ML, LH, AH, VK, LQ, JB, KP, JN, GSC, and MC helped with data acquisition or interpretation. ML wrote the analysis code and the first draft of the manuscript. All authors critically reviewed and contributed to the intellectual content of the manuscript. ML is the guarantor. The corresponding author attests that all listed authors meet authorship criteria and that no others meeting the criteria have been omitted.

Transparency: The lead author affirms that this manuscript is an honest, accurate, and transparent account of the study being reported; that no important aspects of the study have been omitted; and that any discrepancies from the study as planned (and, if relevant, registered) have been explained.

Dissemination to participants and related patient and public communities: The results of this systematic review and network meta-analysis will be shared through presentations to patients and public involvement groups, through Oxford University Hospitals and University of Oxford networks including the NIHR Biomedical Research Centre Oxford, on publicly accessible web pages and social media, at clinical and academic conferences, in presentations to national and international guideline development groups, and in educational sessions for healthcare providers.

Provenance and peer review: Not commissioned; externally peer reviewed.
