# Supplementary material for: Effectiveness of drug interventions to prevent delirium after surgery for older adults: systematic review and network meta-analysis of randomised controlled trials
Source: BMJ. 2026 Feb 12;392:e085539. doi: 10.1136/bmj-2025-085539 (PMC12895303; doi:10.1136/bmj-2025-085539)
Supplement: Supplementary file 1 — Web appendix: Supplementary file 1 [file lunm085539.ww1.pdf]

Supplementary file 1

Table of Contents

Figures ***3***

Tables ***5***

Section 1. Search strategies ***7***

Search Strategy – Medline **7**

Search strategy – EMBASE **10**

Search strategy – CENTRAL (Cochrane Database of Trials) **14**

Section 2. Plain English Summary ***17***

Section 3. Trial exclusion decisions ***18***

Section 4. Global model effect estimates ***19***
Sensitivity analysis on primary outcome with informative priors 21

Section 5. Additional subgroup results ***23***

Network plots **23**
Thoracic surgery 23
Trials enrolling elective and emergency cases 23

Forest plots **24**
Thoracic surgery 24
Trials enrolling elective and emergency cases 25

Effect estimates **26**
Excluding trials at high risk of bias 26
Excluding sparse trial data 28

Section 6. Effect modification by type of anaesthesia ***29***
General anaesthesia only 30
Regional anaesthesia only 34
Trials using general or regional anaesthesia 37

Section 7. Rankograms and SUCRA values ***45***
All operations 45
Hip fracture surgery 48
Cardiac surgery 50
Non cardiac surgery 52
Abdominal surgery 55
Orthopaedic surgery 57
Thoracic Surgery 59
Elective Surgery 60
Emergency Surgery 63
Mixed urgency of surgery (elective and emergency) 65
Excluding high risk of bias trials 66
Excluding sparse trial data 68

Section 8. Inconsistency checking ***70***

Global Model - Residual deviance **70**

Node splitting **70**

Section 9. Individual Risk of Bias assessments ***72***

Section 10. CINeMA quality assessments ... 73
Implementation of CINeMA framework ... 73
Credibility assessments (CINeMA framework) ... 74
Section 11. Pairwise comparisons ... 81
League table of all pairwise comparisons for primary outcome ... 81
Section 12. Secondary Outcomes ... 82
Mortality ... 82
Delirium severity ... 85
Length of stay ... 88
Quality of life ... 91
Postoperative neurocognitive dysfunction (≥3 months) ... 92
Postoperative complications ... 93
Postoperative complications reported in trials of dexmedetomidine, corticosteroids, melatonin receptor agonists, parecoxib, olanzapine or insulin ... 96
Adverse events NOS / Unspecified adverse event(s) ... 97
Arrhythmias ... 98
Bradycardia ... 99
Cardiac complications ... 100
Hypotension ... 101
Hypoxia ... 102
Infection ... 103
Neurological ... 104
Other complications ... 105
Postoperative nausea and vomiting (PONV) ... 106
Renal ... 107
Respiratory ... 108
Serious adverse event(s) ... 109
Sedation ... 110
Tachycardia ... 111
Extrapyramidal side effects (EPSE) ... 112
Hypoglycaemia ... 112
Local anaesthetic systemic toxicity (LAST) ... 112
Suspected unexpected severe adverse reaction(s) (SUSAR) ... 112
Outcomes with limited data ... 113
Cost effectiveness of pharmacological prophylaxis ... 113
Transfer to institutional care ... 113
Time to return to original place of residence ... 113
References for supplementary file 1 ... 114

Figures

Figure 1 Network graph depicting the connectedness of the network for thoracic surgery 23
Figure 2 Network graph depicting the connectedness of the network for trials in elective and emergency surgical settings 24
Figure 3 Forest plot of network meta-analysis results for prevention of delirium following thoracic surgery 24
Figure 4 Forest plot of network meta-analysis results for prevention of delirium in trials which included participants from both elective and emergency settings 25
Figure 5 Network graph depicting the connectedness of the network for trials where all participants received general anaesthesia 30
Figure 6 Forest plot of network meta-analysis results for prevention of delirium in trials where all participants received general anaesthesia 31
Figure 7 Network graph depicting the connectedness of the network for trials in which no participant received general anaesthesia 34
Figure 8 Forest plot of network meta-analysis results for prevention of delirium in trials where no participant received general anaesthesia 35
Figure 9 Network graph depicting the connectedness of the network for trials which included participants receiving general or regional anaesthesia 37
Figure 10 Forest plot of network meta-analysis results for prevention of delirium in trials which included participants receiving general or regional anaesthesia 37
Figure 11 Network graph depicting the connectedness of the network for trials with a mean age of participants under 80 years of age 39
Figure 12 Forest plot of network meta-analysis results for prevention of delirium in trials with a mean age of participants under 80 years of age 40
Figure 13 Network graph depicting the connectedness of the network for trials with a mean age of participants over 80 years of age 43
Figure 14 Forest plot of network meta-analysis results for prevention of delirium in trials with a mean age of participants over 80 years of age 44
Figure 15 Cumulative ranking probabilities for all interventions across all trials 45
Figure 16 Cumulative ranking probabilities for interventions in hip fracture surgery 48
Figure 17 Cumulative ranking probabilities for interventions in cardiac surgery 50
Figure 18 Cumulative ranking probabilities for interventions in non-cardiac surgery 52
Figure 19 Cumulative ranking probabilities for interventions in abdominal surgery .. 55
Figure 20 Cumulative ranking probabilities for interventions in orthopaedic surgery 57
Figure 21 Cumulative ranking probabilities for interventions in thoracic surgery 59
Figure 22 Cumulative ranking probabilities for interventions studied in trials in the emergency surgical setting 60
Figure 23 Cumulative ranking probabilities for interventions studied in trials in the emergency surgical setting 63
Figure 24 Cumulative ranking probabilities for interventions studied in trials of both elective and emergency surgical settings 65
Figure 25 Cumulative ranking probabilities for interventions excluding trials at high risk of bias 66
Figure 26 Cumulative ranking probabilities for interventions studied in more than one trial and trials of at least 100 participants 68
Figure 27 Posterior distributions for direct, indirect and network estimates assessing loop inconsistency 70
Figure 28 Posterior distributions for the inconsistency factors 71

Figure 29 Network graph depicting the connectedness of the network for delirium prevention trials reporting mortality within 1 month of surgery 82
Figure 30 Forest plot of network meta-analysis results for mortality following surgery in delirium prevention trials 83
Figure 31 Network graph depicting the connectedness of the network for trials reporting delirium severity 85
Figure 32 Forest plot of network meta-analysis results for reduction in delirium severity following surgery 86
Figure 33 Network graph depicting the connectedness of the network for trials reporting length of stay in delirium prevention trials 88
Figure 34 Forest plot of network meta-analysis results for postoperative length of stay in delirium prevention trials 89
Figure 35 Network graph for the secondary outcome: Complications (Adverse events, not otherwise specified) 97
Figure 36 Forest plot of network meta-analysis results for secondary outcome: Complications (adverse events not otherwise specified) 97
Figure 37 Network graph for the secondary outcome: Complications (arrhythmia) .. 98
Figure 38 Forest plot of network meta-analysis results for secondary outcome: Complications (arrhythmia) 98
Figure 39 Network graph for the secondary outcome: Complications (bradycardia) 99
Figure 40 Forest plot of network meta-analysis results for secondary outcome: Complications (arrhythmias) 99
Figure 41 Network graph for the secondary outcome: Complications (Cardiac) 100
Figure 42 Forest plot of network meta-analysis results for secondary outcome: Complications (arrhythmias) 100
Figure 43 Network graph for the secondary outcome: Complications (hypotension) 101
Figure 44 Forest plot of network meta-analysis results for secondary outcome: Complications (arrhythmias) 101
Figure 45 Network graph for the secondary outcome: Complications (hypoxia) 102
Figure 46 Forest plot of network meta-analysis results for secondary outcome: Complications (arrhythmias) 102
Figure 47 Network graph for the secondary outcome: Complications (infection) ... 103
Figure 48 Forest plot of network meta-analysis results for secondary outcome: Complications (arrhythmias) 103
Figure 49 Network graph for the secondary outcome: Complications (neurological) 104
Figure 50 Forest plot of network meta-analysis results for secondary outcome: Complications (arrhythmias) 104
Figure 51 Network graph for the secondary outcome: Complications (other) 105
Figure 52 Forest plot of network meta-analysis results for secondary outcome: Complications (arrhythmias) 105
Figure 53 Network graph for the secondary outcome: Complications (postoperative nausea and vomiting) 106
Figure 54 Forest plot of network meta-analysis results for secondary outcome: Complications (arrhythmias) 106
Figure 55 Network graph for the secondary outcome: Complications (renal) 107
Figure 56 Forest plot of network meta-analysis results for secondary outcome: Complications (arrhythmias) 107
Figure 57 Network graph for the secondary outcome: Complications (respiratory) 108

Figure 58 Forest plot of network meta-analysis results for secondary outcome: Complications (arrhythmias)........ 108
Figure 59 Network graph for the secondary outcome: Complications (serious adverse events)........ 109
Figure 60 Forest plot of network meta-analysis results for secondary outcome: Complications (arrhythmias)........ 109
Figure 61 Network graph for the secondary outcome: Complications (sedation) ... 110
Figure 62 Forest plot of network meta-analysis results for secondary outcome: Complications (arrhythmias)........ 110
Figure 63 Network graph for the secondary outcome: Complications (tachycardiac) 111
Figure 64 Forest plot of network meta-analysis results for secondary outcome: Complications (arrhythmias)........ 111

Tables

Table 1 Effect estimates from global model including all operations & all interventions studied for delirium prevention. 19
Table 2 Effect estimates from global model including all operations & all interventions studied for delirium prevention using informative prior distribution for heterogeneity 21
Table 3 Effect estimates for interventions to prevent delirium in trials not at high risk of bias........ 26
Table 4 Effect estimates for interventions to prevent delirium after excluding studies with fewer than 100 participants and interventions studied in only one trial. 28
Table 5 Effect estimates for interventions in trials where all participants received general anaesthesia........ 32
Table 6 Effect estimates for interventions in trials where no participant received general anaesthesia........ 36
Table 7 Effect estimates for interventions in trials which included participants receiving general or regional anaesthesia........ 38
Table 8 Effect estimates for interventions in trials with a mean age of participants under 80 years of age 40
Table 9 Effect estimates for interventions in trials with a mean age of participants over 80 years of age........ 44
Table 10 Surface under the cumulative ranking values for global model (all interventions in all trials)........ 45
Table 11 Surface under the cumulative ranking values for hip fracture surgery....... 48
Table 12 Surface under the cumulative ranking values for cardiac surgery 50
Table 13 Surface under the cumulative ranking values for non-cardiac surgery...... 52
Table 14 Surface under the cumulative ranking values for abdominal surgery 55
Table 15 Surface under the cumulative ranking values for orthopaedic surgery..... 57
Table 16 Surface under the cumulative ranking values for thoracic surgery 59
Table 17 Surface under the cumulative ranking values for trials of both elective surgical setting 60
Table 18 Surface under the cumulative ranking values for trials in the emergency surgical setting 63
Table 19 Surface under the cumulative ranking values for trials of both elective and emergency surgical settings........ 65

Table 20 Surface under the cumulative ranking values excluding trials at high risk of bias.. 66
Table 21 Surface under the cumulative ranking values excluding sparse trial data . 68
Table 22 Confidence in the quality of the evidence using the CINeMA framework .. 74
Table 23 Effect estimates including all interventions reporting mortality within 1 month of surgery 83
Table 24 Effect estimates for delirium severity (all interventions studied) 86
Table 25 Effect estimates for length of stay in delirium prevention trials.................. 89
Table 26 Summary of quality of life measure results in delirium prevention trials 91
Table 27 Secondary outcome: Postoperative cognitive dysfunction measured at timepoints later than discharge from hospital 92
Table 28 Secondary outcome: Postoperative complications by number of trials reporting each complication 93
Table 29 Studies reporting discharge destination of participants by treatment allocation 113

Section 1. Search strategies

Search Strategy – Medline

[RCT filter]

1. exp randomized controlled trial/
2. controlled clinical trial.pt.
3. randomized.ab.
4. placebo.ab.
5. drug therapy.fs.
6. randomly.ab.
7. trial.ab.
8. groups.ab.
9. or/1-8
10. exp animals/ not humans.sh.
11. 9 not 10

[Delirium condition]

12. delirium.mp.
13. delirious.mp.
14. confus*.mp.
15. exp Confusion/
16. cognitive dysfunction*.mp.
17. cognitive dysfunction/ or postoperative cognitive complications/
18. cognitive disorder*.mp.
19. cognitive impairment.mp.
20. neurocognitive disorder*.mp.
21. or/12-20

[Setting/Time]

22. perioperative.mp.
23. peri-operative.mp.
24. exp Perioperative Care/
25. intraoperative.mp.
26. intra-operative.mp.
27. postoperative.mp.
28. post-operative.mp.
29. An?esthe*.mp.
30. exp "Anesthesia and Analgesia"/
31. Post surgical.mp.
32. operat*.mp.
33. Surgery.mp.
34. Post surgical.mp.
35. Postoperative Complications/
36. or/22-35

[Preventative steps]

37. (pharmacologic* adj10 delirium).mp.
38. (pre-emptive* adj10 delirium).mp.
39. Prophylaxis.mp.
40. prophylactic*.mp.
41. (drug* adj10 delirium).mp.
42. (protect* adj10 delirium).mp.
43. (prevent* adj10 delirium).mp.
44. (prior adj7 surgery).mp.

[Drug classes/anaesthesia methods]

45. exp Adrenergic alpha-2 Receptor Agonists/
46. exp Methyl Ethers/
47. Inhalational.mp.
48. volatile.mp.
49. volatile an?esthe*.mp.
50. inhalational an?esthe*.mp.
51. TIVA.mp.
52. Total intravenous an?esthe*.mp.
53. cox inhibitor.mp.
54. cholinesterase inhibitor.mp.
55. acetylcholinesterase inhibitor.mp.
56. saline.mp.
57. nsaid*.mp.
58. non-steroidal anti-inflammatory drug*.mp.
59. anti-inflammatory.mp.
60. monoclonal antibod*.mp.
61. steroid.mp.
62. glucocorticoid.mp.
63. antipsychotic.mp.
64. neuropathic.mp.
65. opioid.mp.
66. benzodiazepine.mp.
67. cannabinoid.mp.

[Specific drug names]

68. propofol.mp.
69. Etomidate.mp.
70. ketamine.mp.
71. dexmedetomidine.mp.
72. sevoflurane.mp.
73. desflurane.mp.
74. isoflurane.mp.
75. lidocaine.mp.
76. midazolam.mp.
77. remimazolam.mp.

78. diazepam.mp.
79. lorazepam.mp.
80. paracetamol.mp.
81. acetaminophen.mp.
82. celecoxib.mp.
83. paracoxib.mp.
84. naproxen.mp.
85. diclofenac.mp.
86. ketorolac.mp.
87. aspirin.mp.
88. ibuprofen.mp.
89. dexamethasone.mp.
90. hydrocortisone.mp.
91. haloperidol.mp.
92. olanzapine.mp.
93. aripiprazole.mp.
94. risperidone.mp.
95. quetiapine.mp.
96. gabapentin.mp.
97. pregabalin.mp.
98. morphine.mp.
99. fentanyl.mp.
100. oxycodone.mp.
101. alfentanil.mp.
102. remifentanil.mp.
103. zopiclone.mp.
104. melatonin.mp.
105. ramelteon.mp.
106. rivastigmine.mp.
107. donepezil.mp.
108. or/37-107

[Exclusions]

109. (case reports or editorial or letter or "review" or "systematic review").pt.
110. child*.ti.
111. p?ediatric*.ti.
112. or/109-111

[Final search]

113. 11 and 21 and 36 and 108
114. 113 not 112

Search strategy – EMBASE

[Delirium condition]

1. delirium.mp.
2. delirious.mp.
3. confus*.mp.
4. exp Confusion/
5. "cognitive dysfunction*".mp.
6. "cognitive dysfunction"/ or "postoperative cognitive complications"/
7. "cognitive disorder*".mp.
8. "cognitive impairment".mp.
9. "neurocognitive disorder*".mp.
10. or/1-9

[Setting/Time]

11. perioperative.mp.
12. peri-operative.mp.
13. exp "Perioperative Care"/
14. intraoperative.mp.
15. intra-operative.mp.
16. postoperative.mp.
17. post-operative.mp.
18. An?esthe*.mp.
19. exp "Anesthesia and Analgesia"/
20. "Post surgical".mp.
21. operat*.mp.
22. Surgery.mp.
23. "postoperative Complications"/
24. postoperative complication/
25. exp surgery/
26. exp anesthesia complication/
27. exp perioperative complication/ or exp peroperative complication/ or exp postoperative complication/ or exp preoperative complication/
28. or/11-27

[Preventative steps]

29. prophylaxis.mp.
30. prophylactic*.mp.
31. (pre-emptive* adj10 delirium).mp.
32. (preemptive* adj10 delirium).mp.
33. (prior adj7 surgery).mp.
34. (prevent* adj10 delirium).mp.
35. (protect* adj10 delirium).mp.
36. (pharmacologic* adj10 delirium).mp.
37. (drug* adj10 delirium).mp.

[Drug classes/anaesthesia methods]

38. exp alpha 2 adrenergic receptor stimulating agent/
39. exp ether derivative/
40. inhalational drug administration/
41. volatile.mp.
42. volatile an?esthe*.mp.
43. intravenous anesthesia/ or intravenous anesthetic agent/
44. exp cyclooxygenase 1 inhibitor/ or exp cyclooxygenase 2 inhibitor/
45. exp cholinesterase inhibitor/
46. acetylcholinesterase inhibitor.mp.
47. sodium chloride/
48. exp nonsteroid antiinflammatory agent/
49. anti-inflammatory.mp.
50. exp monoclonal antibody/
51. exp steroid/
52. exp glucocorticoid/
53. exp neuroleptic agent/
54. neuropathic.mp.
55. exp opiate agonist/
56. exp benzodiazepine/
57. exp cannabinoid/

[Specific drug names]

58. propofol.mp.
59. etomidate.mp.
60. ketamine.mp.
61. dexmedetomidine.mp.
62. clonidine.mp.
63. sevoflurane.mp.
64. desflurane.mp.
65. isoflurane.mp.
66. lidocaine.mp.
67. midazolam.mp.
68. remimazolam.mp.
69. diazepam.mp.
70. lorazepam.mp.
71. temazepam.mp.
72. paracetamol.mp.
73. acetaminophen.mp.
74. celecoxib.mp.
75. paracoxib.mp.
76. naproxen.mp.
77. diclofenac.mp.
78. ketorolac.mp.
79. aspirin.mp.
80. ibuprofen.mp.
81. dexamethasone.mp.
82. hydrocortisone.mp.
83. methylprednisolone.mp.

84. haloperidol.mp.
85. olanzapine.mp.
86. aripiprazole.mp.
87. risperidone.mp.
88. quetiapine.mp.
89. gabapentin.mp.
90. pregabalin.mp.
91. morphine.mp.
92. fentanyl.mp.
93. oxycodone.mp.
94. alfentanil.mp.
95. remifentanil.mp.
96. zopiclone.mp.
97. melatonin.mp.
98. ramelteon.mp.
99. donepezil.mp.
100. rivastigmine.mp.
101. or/29-100

[RCT filter]

102. exp randomized controlled trial/
103. controlled clinical trial/
104. random$.ti,ab.
105. randomization/
106. intermethod comparison/
107. placebo.ti,ab.
108. (compare or compared or comparison).ti,ab.
109. ((evaluated or evaluate or evaluating or assessed or assess) and (compare or compared or comparing or comparison)).ab.
110. (open adj label).ti,ab.
111. ((double or single or doubly or singly) adj (blind or blinded or blindly)).ti,ab.
112. double blind procedure/
113. parallel group$1.ti,ab.
114. (crossover or cross over).ti,ab.
115. ((assign$ or match or matched or allocation) adj5 (alternate or group$1 or intervention$1 or patient$1 or subject$1 or participant$1)).ti,ab.
116. (assigned or allocated).ti,ab.
117. (controlled adj7 (study or design or trial)).ti,ab.
118. (volunteer or volunteers).ti,ab.
119. human experiment/
120. trial.ti.
121. or/102-120
122. (random$ adj sampl$ adj7 ("cross section$" or questionnaire$1 or survey$ or database$1)).ti,ab. not (comparative study/ or controlled study/ or randomi?ed controlled.ti,ab. or randomly assigned.ti,ab.)
123. cross-sectional study/ not (exp randomized controlled trial/ or controlled clinical trial/ or controlled study/ or randomi?ed controlled.ti,ab. or control group$1.ti,ab.)
124. (((case adj control$) and random$) not randomi?ed controlled).ti,ab.

125. systematic review.ti,ab. not (trial or study).ti.
126. (nonrandom$ not random$).ti,ab.
127. "random field$".ti,ab.
128. (random cluster adj3 sampl$).ti,ab.
129. (review.ab. and review.pt.) not trial.ti.
130. "we searched".ab. and (review.ti. or review.pt.)
131. "update review".ab.
132. (databases adj4 searched).ab.
133. (rat or rats or mouse or mice or swine or porcine or murine or sheep or lambs or pigs or piglets or rabbit or rabbits or cat or cats or dog or dogs or cattle or bovine or monkey or monkeys or trout or marmoset$1).ti. and animal experiment/
134. animal experiment/ not (human experiment/ or human/)
135. or/122-134
136. 121 not 135

[Filter]

137. article.pt.

[Combine]

138. 10 and 28 and 101 and 136 and 137

[Exclusions]

139. (child* or p?ediatric*).ti.
140. 138 not 139

Search strategy – CENTRAL (Cochrane Database of Trials)

[Delirium condition]

#1 delirium:ti,ab,kw 5613
#2 delirious:ti,ab,kw 196
#3 confus*:ti,ab,kw 4390
#4 [mh Delirium] 1477
#5 [mh Confusion] 1727
#6 ("cognitive" NEXT dysfunction*):ti,ab,kw 5710
#7 [mh ^"cognitive Dysfunction"] 3121
#8 [mh ^"Postoperative Cognitive Complications"] 79
#9 ("cognitive" NEXT disorder*):ti,ab,kw 860
#10 "cognitive impairment":ti,ab,kw 12123
#11 ("neurocognitive" NEXT disorder*):ti,ab,kw 615
#12 [mh "Neurocognitive Disorders"] 17100
#13 [mh "Cognitive Dysfunction"] 3175
#14 OR #1-#13 34878

[Setting/Time]

#15 perioperative:ti,ab,kw 25566
#16 peri-operative:ti,ab,kw 2515
#17 [mh "Perioperative Care"] 14526
#18 intraoperative:ti,ab,kw 34326
#19 intra-operative:ti,ab,kw 4254
#20 postoperative:ti,ab,kw 152530
#21 post-operative:ti,ab,kw 26335
#22 An?esthe*:ti,ab,kw 98278
#23 [mh "Anesthesia and Analgesia"] 35677
#24 "Post surgical":ti,ab,kw 2597
#25 operat*:ti,ab,kw 130495
#26 Surgery:ti,ab,kw 276898
#27 [mh ^"postoperative Complications"] 20860
#28 OR #15-#27 390455

[Preventative steps]

#29 prophylaxis:ti,ab,kw 29574
#30 prophylactic*:ti,ab,kw 19395
#31 (pre-emptive*:ti,ab,kw NEAR/10 delirium:ti,ab,kw) 2
#32 (preemptive*:ti,ab,kw NEAR/10 delirium:ti,ab,kw) 5
#33 (prior:ti,ab,kw NEAR/7 surgery:ti,ab,kw) 8094
#34 (prevent*:ti,ab,kw NEAR/10 delirium:ti,ab,kw) 1131
#35 (protect*:ti,ab,kw NEAR/10 delirium:ti,ab,kw) 35
#36 (pharmacologic*:ti,ab,kw NEAR/10 delirium:ti,ab,kw) 179
#37 (drug*:ti,ab,kw NEAR/10 delirium:ti,ab,kw) 1023

[Specific drug names]

#38 propofol:ti,ab,kw 18339
#39 etomidate:ti,ab,kw 1363
#40 ketamine:ti,ab,kw 7054
#41 dexmedetomidine:ti,ab,kw 8826
#42 sevoflurane:ti,ab,kw 6981
#43 desflurane:ti,ab,kw 1939
#44 isoflurane:ti,ab,kw 4431
#45 lidocaine:ti,ab,kw 15633
#46 midazolam:ti,ab,kw 10268
#47 remimazolam:ti,ab,kw 746
#48 diazepam:ti,ab,kw 4252
#49 lorazepam:ti,ab,kw 2054
#50 temazepam:ti,ab,kw 446
#51 paracetamol:ti,ab,kw 7823
#52 acetaminophen:ti,ab,kw 7342
#53 celecoxib:ti,ab,kw 2611
#54 paracoxib:ti,ab,kw 10
#55 naproxen:ti,ab,kw 2577
#56 diclofenac:ti,ab,kw 5954
#57 ketorolac:ti,ab,kw 3296
#58 aspirin:ti,ab,kw 16040
#59 ibuprofen:ti,ab,kw 5719
#60 dexamethasone:ti,ab,kw 15366
#61 hydrocortisone:ti,ab,kw 10400
#62 methylprednisolone:ti,ab,kw 6131
#63 haloperidol:ti,ab,kw 3331
#64 olanzapine:ti,ab,kw 3845
#65 aripiprazole:ti,ab,kw 1796
#66 risperidone:ti,ab,kw 3782
#67 quetiapine:ti,ab,kw 2058
#68 gabapentin:ti,ab,kw 2843
#69 pregabalin:ti,ab,kw 2621
#70 morphine:ti,ab,kw 17044
#71 fentanyl:ti,ab,kw 17804
#72 oxycodone:ti,ab,kw 2940
#73 alfentanil:ti,ab,kw 1603
#74 remifentanil:ti,ab,kw 6025
#75 zopiclone:ti,ab,kw 426
#76 melatonin:ti,ab,kw 3833
#77 ramelteon:ti,ab,kw 233
#78 donepezil:ti,ab,kw 1871
#79 rivastigmine:ti,ab,kw 750
#80 clonidine:ti,ab,kw 4680

[Drug classes/anaesthesia methods]

#81 [mh ^"adrenergic alpha-2 Receptor Agonists"] 328
#82 [mh "Anesthesia and Analgesia"] 35677
#83 volatile:ti,ab,kw 2878
#84 (volatile NEXT/2 an?esthe*):ti,ab,kw 711
#85 [mh ^"intravenous anesthesia"] 2036
#86 [mh "cholinesterase inhibitor"] 1130
#87 "acetylcholinesterase inhibitor":ti,ab,kw 338
#88 [mh ^"sodium chloride"] 2397
#89 [mh "Anti-Inflammatory Agents, Non-Steroidal"] 8784
#90 anti-inflammatory:ti,ab,kw 32172
#91 [mh "Antibodies, Monoclonal"] 19082
#92 [mh steroid] 69108
#93 [mh glucocorticoid] 5364
#94 neuropathic:ti,ab,kw 5502
#95 [mh "Analgesics, Opioid"] 9760
#96 [mh benzodiazepine] 10672
#97 [mh cannabinoid] 1210

All interventions
#98 OR #29-#97 324235

Condition + Timing + intervention
#99 #14 AND #28 AND #98 3881

[Exclusions]

#100 p?ediatric*:ti 17866
#101 child*:ti 84347
#102 #100 OR #101 100992

Final search
#103 #99 not #102 3284

Section 2. Plain English Summary

What is this summary about?
Among older adults confusion and memory problems after surgery are common. This is called delirium. People who get delirium spend longer in hospital, are more likely to die in hospital or develop dementia later in life. Delirium is often unpleasant and distressing. Doctors are unsure which medicines work best to stop people getting delirium after surgery.

How was the study done?
The researchers analysed medicines to prevent delirium after surgery in studies of people over 60 years old. They used results from all these studies with a method called network meta-analysis to compare the medicines. This allowed the researchers to work out which medicines are likely to work and by how much compared to each other.

What did the study find and what does that mean?
They found 158 studies involving 41084 people. The medicine dexmedetomidine, a painkiller and sedative that also reduces inflammation was found to prevent delirium in almost every type of surgery. It reduced the number of people with delirium after surgery from 14 to 7 per 100 people.

Several other medicines worked in specific types of surgery but often only in smaller studies. The type of anaesthetic had minimal effect on whether delirium was prevented. Time spent in hospital and deaths after surgery were not different whether people had treatment to prevent delirium or not.

Complications were generally no different with treatment compared to placebo. Lower blood pressure or slower heart rate were more common with dexmedetomidine but this did not result in more serious problems such as heart attacks or strokes. Dexmedetomidine treatment led to less nausea and vomiting after surgery. People given steroids had slightly less severe delirium.

Who should read this summary?
This summary was written in partnership between researchers, doctors, patient and public involvement partners with lived experience of surgery. The researchers hope these findings can help people expecting surgery and healthcare providers with ways to reduce delirium after surgery and inform policy makers to improve clinical guidelines.

Section 3. Trial exclusion decisions

Although ostensibly meeting eligibility criteria the following studies or individual arms were not included in the final analysis. Amin and colleagues' study of lidocaine versus fentanyl had zero events in all comparison arm (no episodes of delirium).[1] In Ma and colleagues multi-arm study of dexmedetomidine and ketamine the comparison of combined dexmedetomidine+ketamine was excluded as zero events occurred and this was the only study with that particular intervention.[2]

Within speciality specific networks zero event comparisons remifentanil, remimazolam and acetaminophen were excluded from non-cardiac, abdominal and elective surgery analyses respectively.[3-5] The Cochrane handbook recommends exclusion of both arm zero event studies.[6] Whilst work by Xu and colleagues showed this can alter conclusions of meta-analysis similar work has not been done for network meta-analysis.[7] Simulation experiments have shown either route is reasonable.[8] Spence and colleagues cluster randomized crossover single blind pilot trial of two strategies of benzodiazepine administration during cardiac surgery was excluded.[9] Having carefully considered the potential statistical methods available,[10] [11] absent robust data on cluster and period correlations we excluded this trial from the analysis. The outcome assessors were also not masked to group allocation in that trial.

Section 4. Global model effect estimates

Lower values favour intervention over placebo. Placebo is reference value.

Table 1 Effect estimates from global model including all operations & all interventions studied for delirium prevention.

Intervention	OR (95%CrI)	Trials	Participants
Diazepam+flunitrazepam+pethidine	0.07 (0.00-0.69)	1	20
Insulin (intranasal)	0.12 (0.04-0.35)	3	105
Haloperidol+ketamine	0.21 (0.03-1.07)	1	46
Aprotinin	0.22 (0.01-3.13)	1	18
Methylene blue	0.24 (0.06-1.01)	1	124
Dexmedetomidine (tracheal)	0.25 (0.05-1.10)	1	50
Thiamine	0.25 (0.04-1.35)	1	48
Risperidone	0.26 (0.05-1.18)	1	63
Dexmedetomidine+atorvastatin	0.26 (0.01-2.94)	1	20
Olanzapine	0.27 (0.07-1.02)	1	243
Cyproheptadine	0.29 (0.04-1.88)	1	23
Caffeine	0.31 (0.06-1.49)	1	34
Propofol+acetaminophen	0.31 (0.06-1.42)	1	31
Ketofol	0.32 (0.03-2.21)	1	40
Ciprofol	0.32 (0.04-2.29)	1	53
Parecoxib	0.35 (0.16-0.78)	4	425
Edavarone	0.38 (0.09-1.53)	1	80
Flurbiprofen	0.38 (0.08-1.88)	1	60
Dexmedetomidine	0.45 (0.36-0.56)	73	7,828
Rivastigime	0.47 (0.19-1.16)	3	138
Donepezil	0.48 (0.09-2.24)	2	28
Corticosteroids	0.48 (0.29-0.79)	9	4,493
Ondansetron	0.49 (0.11-2.03)	1	51
Rosuvastatin	0.52 (0.14-1.87)	1	410

Intervention	OR (95%CrI)	Trials	Participants
Ketamine	0.53 (0.32-0.85)	12	811
Lidocaine	0.53 (0.21-1.34)	3	116
Melatonin receptor agonists	0.54 (0.34-0.84)	12	759
Hydrogen	0.54 (0.12-2.28)	1	85
Remimazolam	0.55 (0.17-1.72)	3	274
Quetiapine	0.56 (0.12-2.65)	1	57
Haloperidol	0.60 (0.31-1.12)	5	594
Gabapentin	0.68 (0.21-2.16)	2	359
Remifentanil	0.73 (0.18-2.88)	2	232
Nitrous oxide	0.76 (0.22-2.85)	2	177
Morphine	0.84 (0.21-3.56)	1	152
Selenium	0.87 (0.25-2.85)	1	697
Xenon	0.90 (0.34-2.45)	4	266
Propofol	0.91 (0.56-1.49)	28	3,527
Iron	0.99 (0.24-4.08)	1	74
Volatile	1.04 (0.54-1.97)	14	2,001
Magnesium	1.07 (0.07-14.64)	1	31
Morphine+midazolam	1.15 (0.06-55.25)	1	35
Tryptophan	1.15 (0.34-4.16)	1	152
Clonidine	1.31 (0.54-3.32)	2	193
Physostigime	1.45 (0.38-5.49)	1	136
Alprazolam	1.51 (0.38-5.79)	1	40
Atropine	1.52 (0.27-9.77)	1	30
Midazolam	1.83 (0.74-4.72)	2	90
Minocycline	1.86 (0.28-15.59)	1	100
Penehyclidine	6.20 (1.19-35.37)	1	30

OR, Odds Ratio; 95%CrI, 95% Credible Interval

Sensitivity analysis on primary outcome with informative priors

Table 2 Effect estimates from global model including all operations & all interventions studied for delirium prevention using informative prior distribution for heterogeneity

Intervention	OR (95%CrI)	Trials	Participants
Diazepam+flunitrazepam+pethidine	0.07 (0.00-0.75)	1	20
Insulin (intranasal)	0.13 (0.04-0.35)	3	105
Haloperidol+ketamine	0.21 (0.04-0.95)	1	46
Aprotinin	0.22 (0.01-3.03)	1	18
Methylene blue	0.24 (0.06-0.95)	1	124
Dexmedetomidine (tracheal)	0.25 (0.05-1.04)	1	50
Risperidone	0.26 (0.05-1.12)	1	63
Thiamine	0.26 (0.05-1.33)	1	48
Dexmedetomidine+atorvastatin	0.26 (0.01-3.03)	1	20
Olanzapine	0.27 (0.08-0.89)	1	243
Cyproheptadine	0.29 (0.04-2.07)	1	23
Caffeine	0.30 (0.06-1.43)	1	34
Ciprofol	0.31 (0.05-2.26)	1	53
Propofol+acetaminophen	0.32 (0.06-1.45)	1	31
Ketofol	0.32 (0.04-2.25)	1	40
Parecoxib	0.35 (0.16-0.77)	4	425
Edavarone	0.38 (0.10-1.52)	1	80
Flurbiprofen	0.38 (0.08-1.66)	1	60
Dexmedetomidine	0.45 (0.36-0.56)	73	7,828
Donepezil	0.46 (0.09-2.23)	2	28
Rivastigime	0.47 (0.20-1.15)	3	138
Ondansetron	0.48 (0.12-1.86)	1	51
Corticosteroids	0.48 (0.28-0.78)	9	4,493
Rosuvastatin	0.53 (0.15-1.90)	1	410
Hydrogen	0.53 (0.13-2.27)	1	85

Intervention	OR (95%CrI)	Trials	Participants
Ketamine	0.53 (0.32-0.84)	12	811
Lidocaine	0.54 (0.21-1.36)	3	116
Melatonin receptor agonists	0.54 (0.34-0.84)	12	759
Quetiapine	0.55 (0.12-2.64)	1	57
Remimazolam	0.55 (0.19-1.72)	3	274
Haloperidol	0.60 (0.32-1.09)	5	594
Gabapentin	0.69 (0.23-2.02)	2	359
Remifentanil	0.74 (0.19-3.03)	2	232
Nitrous oxide	0.75 (0.22-2.59)	2	177
Morphine	0.84 (0.21-3.35)	1	152
Selenium	0.86 (0.28-2.80)	1	697
Xenon	0.90 (0.35-2.47)	4	266
Propofol	0.91 (0.57-1.47)	28	3,527
Iron	1.01 (0.24-3.89)	1	74
Volatile	1.04 (0.55-1.98)	14	2,001
Magnesium	1.05 (0.08-12.40)	1	31
Morphine+midazolam	1.14 (0.07-39.70)	1	35
Tryptophan	1.16 (0.34-4.16)	1	152
Clonidine	1.32 (0.54-3.40)	2	193
Physostigime	1.43 (0.38-5.28)	1	136
Alprazolam	1.50 (0.40-5.61)	1	40
Atropine	1.57 (0.27-9.38)	1	30
Minocycline	1.85 (0.28-13.58)	1	100
Midazolam	1.87 (0.76-4.52)	2	90
Penehyclidine	6.22 (1.20-36.35)	1	30

OR, Odds Ratio; 95%CrI, 95% Credible Interval, prior for Tau^2 log normal(-3.02,1.85) from Turner and colleagues (2012).[12]

Section 5. Additional subgroup results

Network plots

Thoracic surgery

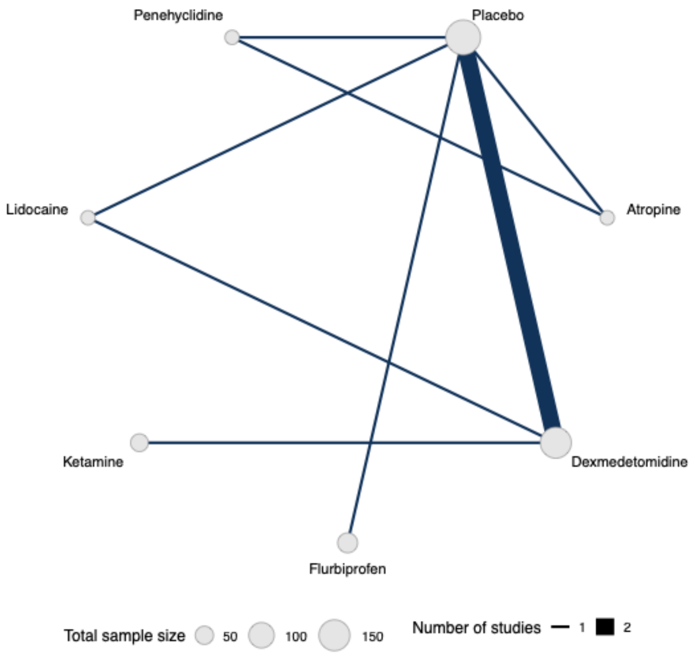

Figure 1 Network graph depicting the connectedness of the network for thoracic surgery

Trials enrolling elective and emergency cases

In these trials some participants had elective surgery and others had emergency surgery.

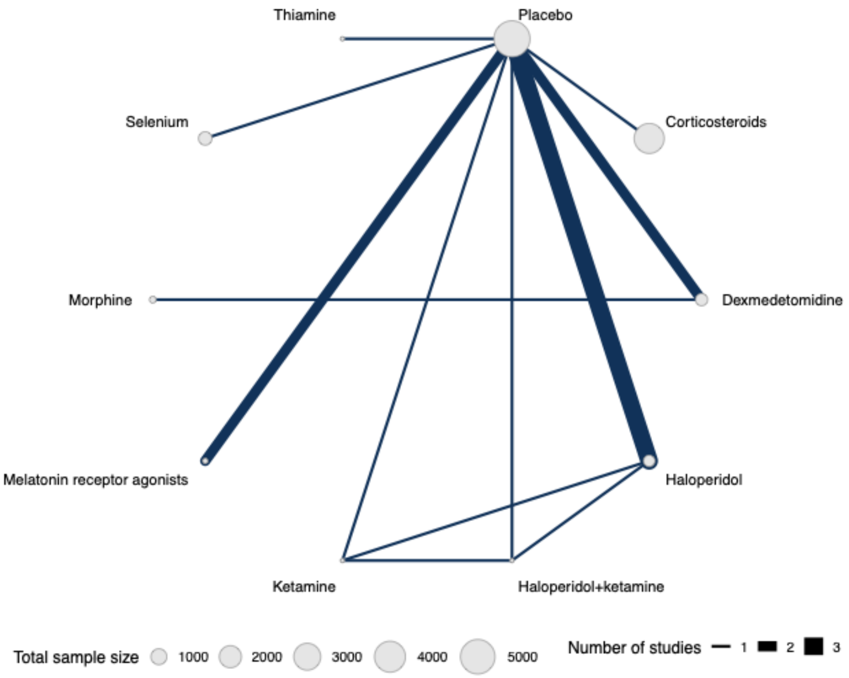

Figure 2 Network graph depicting the connectedness of the network for trials in elective and emergency surgical settings

Forest plots

Treatment effect less than 1 favours intervention. Reference is placebo. Box sizes correspond to number of participants.

Thoracic surgery

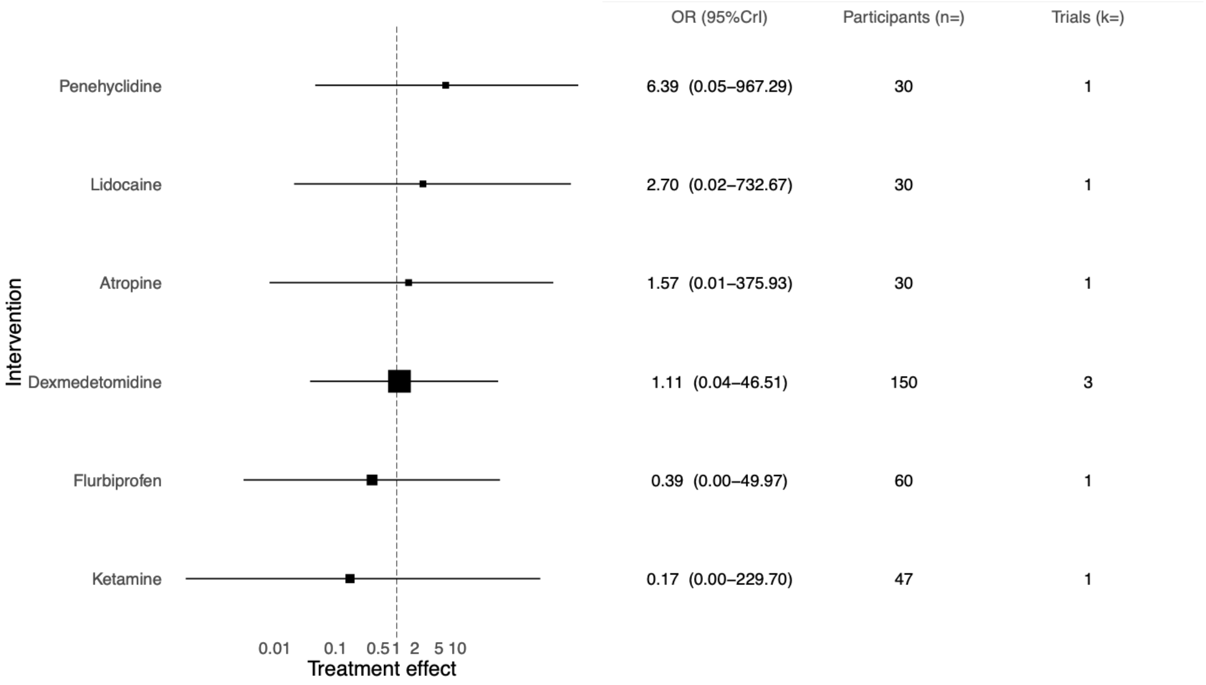

Figure 3 Forest plot of network meta-analysis results for prevention of delirium following thoracic surgery

Trials enrolling elective and emergency cases

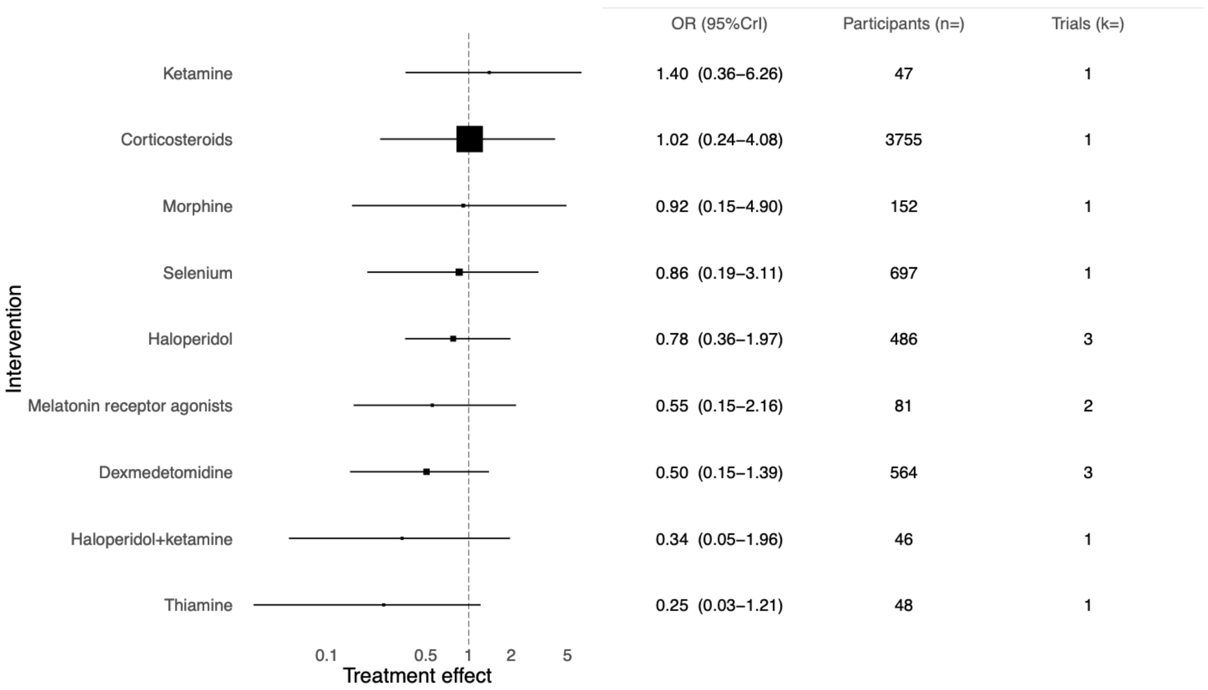

Figure 4 Forest plot of network meta-analysis results for prevention of delirium in trials which included participants from both elective and emergency settings

Effect estimates

Lower values favour intervention over placebo. Placebo is the reference value.

Excluding trials at high risk of bias

Table 3 Effect estimates for interventions to prevent delirium in trials not at high risk of bias

Intervention	OR (95%CrI)	Trials	Participants
Insulin (intranasal)	0.13 (0.04-0.36)	3	105
Aprotinin	0.21 (0.00-3.18)	1	18
Haloperidol+ketamine	0.23 (0.04-1.08)	1	46
Dexmedetomidine (tracheal)	0.25 (0.05-1.11)	1	50
Risperidone	0.25 (0.05-1.16)	1	63
Olanzapine	0.27 (0.07-0.94)	1	243
Caffeine	0.31 (0.06-1.46)	1	34
Propofol+acetaminophen	0.31 (0.06-1.44)	1	31
Ketofol	0.32 (0.04-2.25)	1	40
Parecoxib	0.34 (0.16-0.74)	4	425
Edavarone	0.38 (0.09-1.51)	1	80
Flurbiprofen	0.38 (0.09-1.69)	1	60
Dexmedetomidine	0.46 (0.36-0.57)	66	7,156
Rivastigime	0.47 (0.19-1.16)	3	138
Donepezil	0.47 (0.09-2.39)	2	28
Hydrogen	0.52 (0.12-2.09)	1	85
Rosuvastatin	0.52 (0.14-1.91)	1	410
Remimazolam	0.53 (0.16-1.62)	3	274
Corticosteroids	0.53 (0.31-0.87)	8	4,460
Lidocaine	0.54 (0.20-1.38)	3	116
Melatonin receptor agonists	0.54 (0.34-0.85)	12	759
Quetiapine	0.57 (0.12-2.53)	1	57
Haloperidol	0.60 (0.32-1.11)	5	594
Ketamine	0.62 (0.37-1.06)	10	745

Intervention	OR (95%CrI)	Trials	Participants
Remifentanil	0.74 (0.19-3.02)	2	232
Nitrous oxide	0.79 (0.22-2.58)	2	177
Xenon	0.85 (0.30-2.34)	4	266
Selenium	0.86 (0.26-2.77)	1	697
Morphine	0.87 (0.22-3.42)	1	152
Propofol	0.87 (0.52-1.50)	23	3,124
Volatile	0.98 (0.49-1.95)	12	1,864
Iron	1.01 (0.25-3.86)	1	74
Magnesium	1.02 (0.08-14.79)	1	31
Tryptophan	1.13 (0.33-3.97)	1	152
Morphine+midazolam	1.15 (0.07-38.40)	1	35
Gabapentin	1.20 (0.38-4.04)	1	350
Clonidine	1.33 (0.51-3.29)	2	193
Physostigime	1.43 (0.37-5.69)	1	136
Minocycline	1.83 (0.28-14.59)	1	100
Midazolam	1.87 (0.75-4.65)	2	90

OR, Odds Ratio; 95%CrI, 95% Credible Interval

Excluding sparse trial data

Table 4 Effect estimates for interventions to prevent delirium after excluding studies with fewer than 100 participants and interventions studied in only one trial.

Intervention	OR (95%CrI)	Trials	Participants
Dexmedetomidine	0.52 (0.41-0.63)	48	6,854
Remimazolam	0.59 (0.21-1.55)	3	274
Corticosteroids	0.64 (0.38-1.06)	5	4,347
Rivastigime	0.64 (0.26-1.61)	2	107
Melatonin receptor agonists	0.67 (0.41-1.10)	7	568
Haloperidol	0.72 (0.38-1.36)	3	509
Nitrous oxide	0.80 (0.28-2.30)	2	177
Remifentanil	0.91 (0.24-3.05)	2	232
Xenon	0.91 (0.33-2.38)	2	220
Propofol	0.92 (0.57-1.47)	19	3,223
Volatile	1.00 (0.53-1.80)	10	1,880
Ketamine	1.14 (0.59-2.22)	4	543
Clonidine	1.49 (0.65-3.32)	2	193
Midazolam	2.00 (0.89-4.51)	2	90

OR, Odds Ratio; 95%CrI, 95% Credible Interval

Section 6. Effect modification

Type of anaesthesia

For 127 trials all participants had general anaesthesia (GA) and in 16 trials all participants had regional anaesthesia, primarily spinal anaesthesia (RA). There were seven trials with a mixture of participants receiving either general or regional anaesthesia (‘mixed’). All ‘mixed’ trials were orthopaedic surgery (five fracture neck of femur trials[13-17] and two elective lower limb arthroplasty trials).[18 19] As we report in the main text, we did not find anaesthesia to be an important moderator with the magnitude and direction of treatment effects largely preserved across the interventions for which between-group comparisons could be made (dexmedetomidine, corticosteroids, parecoxib, midazolam).

There were some interventions whose credible intervals were non-significant between-groups (described below), although estimates of treatment effect were comparable and the direction of estimated treatment effect was not different between-groups. Ketamine was effective in general anaesthesia trials OR 0.57 (0.33 to 0.97, n=780, studies=11) but not RA only trials OR 0.21 (0.02 to 1.80). Melatonin receptor agonists were not effective in mixed GA and RA trials; however, there was benefit in the RA only subgroup of trials OR 0.28 (0.09 to 0.80, n= 143, studies=3) and effectiveness in GA trials was OR 0.59 (0.31 to 0.99, n=379, studies=7).

General anaesthesia only

In these trials all participants had general anaesthesia

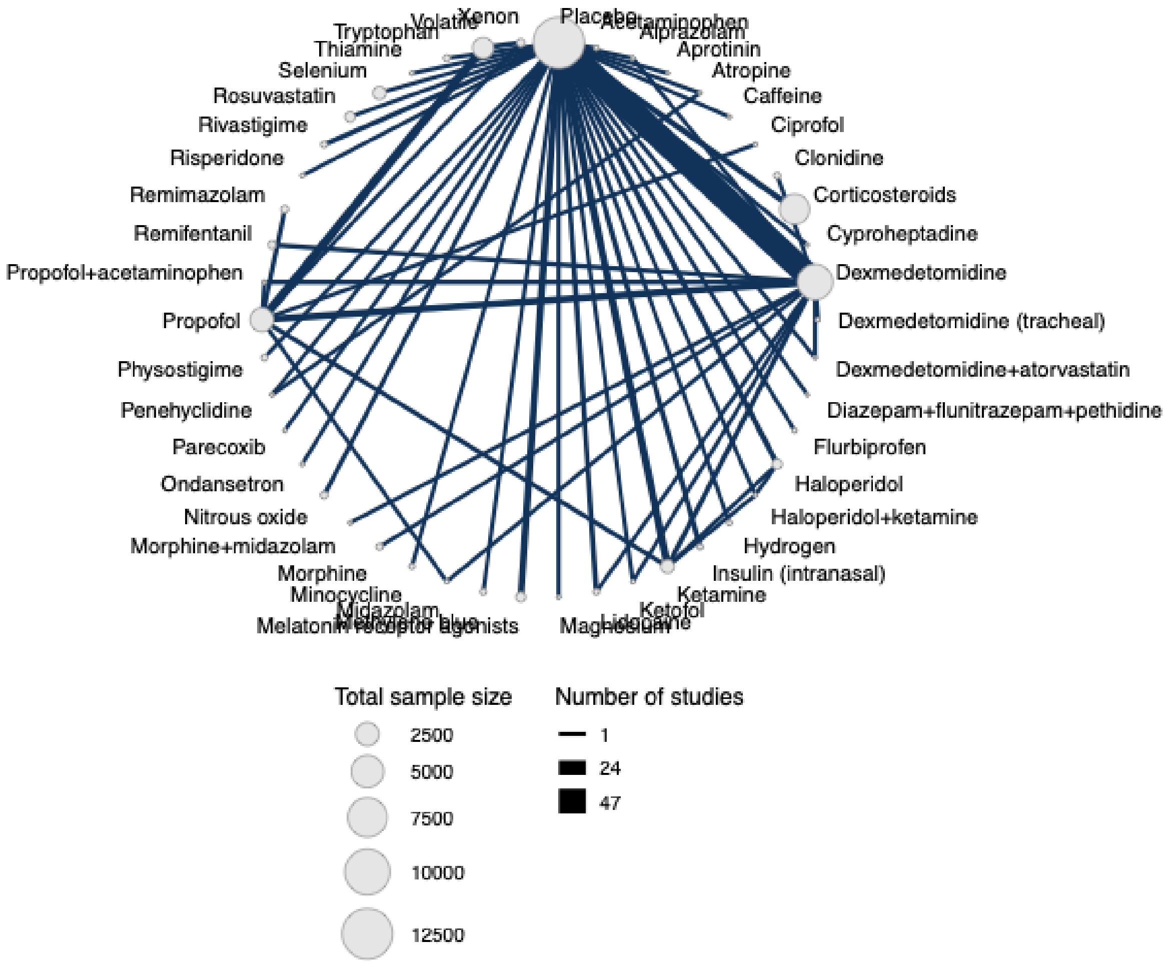

Figure 5 Network graph depicting the connectedness of the network for trials where all participants received general anaesthesia

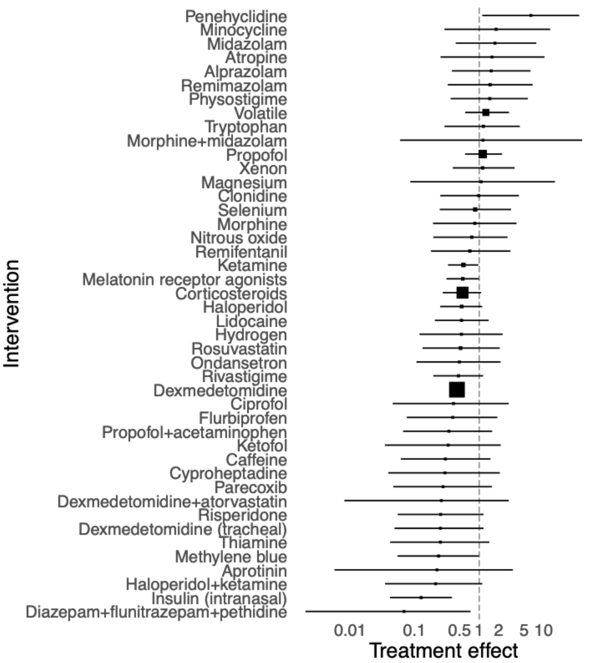

Intervention	OR (95%CrI)	Participants (n=)	Trials (k=)
Penehyclidine	6.32 (1.11–35.48)	30	1
Minocycline	1.83 (0.29–12.59)	100	1
Midazolam	1.75 (0.43–7.67)	40	1
Atropine	1.57 (0.25–10.28)	30	1
Alprazolam	1.53 (0.38–6.25)	40	1
Remimazolam	1.47 (0.32–6.74)	221	2
Physostigime	1.45 (0.36–5.67)	136	1
Volatile	1.27 (0.60–2.90)	2001	14
Tryptophan	1.16 (0.29–4.26)	152	1
Morphine+midazolam	1.14 (0.06–39.31)	35	1
Propofol	1.13 (0.60–2.26)	2610	23
Xenon	1.13 (0.39–3.53)	266	4
Magnesium	1.07 (0.09–14.93)	31	1
Clonidine	0.98 (0.25–4.13)	142	1
Selenium	0.86 (0.24–3.11)	697	1
Morphine	0.85 (0.19–3.79)	152	1
Nitrous oxide	0.77 (0.19–2.75)	177	2
Remifentanil	0.71 (0.18–3.05)	232	2
Ketamine	0.57 (0.33–0.97)	780	11
Melatonin receptor agonists	0.55 (0.31–0.99)	379	7
Corticosteroids	0.55 (0.27–1.07)	4283	5
Haloperidol	0.53 (0.25–1.11)	382	4
Lidocaine	0.53 (0.20–1.40)	116	3
Hydrogen	0.53 (0.12–2.30)	85	1
Rosuvastatin	0.51 (0.13–2.08)	410	1
Ondansetron	0.49 (0.11–2.14)	51	1
Rivastigime	0.48 (0.19–1.15)	138	3
Dexmedetomidine	0.45 (0.35–0.57)	6050	62
Ciprofol	0.40 (0.05–2.86)	53	1
Flurbiprofen	0.39 (0.08–1.91)	60	1
Propofol+acetaminophen	0.34 (0.07–1.58)	31	1
Ketofol	0.33 (0.03–2.15)	40	1
Caffeine	0.30 (0.06–1.49)	34	1
Cyproheptadine	0.29 (0.04–2.09)	23	1
Parecoxib	0.27 (0.05–1.57)	40	1
Dexmedetomidine+atorvastatin	0.26 (0.01–2.87)	20	1
Risperidone	0.25 (0.05–1.17)	63	1
Dexmedetomidine (tracheal)	0.25 (0.05–1.16)	50	1
Thiamine	0.25 (0.04–1.43)	48	1
Methylene blue	0.23 (0.05–0.99)	124	1
Aprotinin	0.22 (0.01–3.33)	18	1
Haloperidol+ketamine	0.21 (0.03–1.11)	46	1
Insulin (intranasal)	0.13 (0.04–0.38)	105	3
Diazepam+flunitrazepam+pethidine	0.07 (0.00–0.73)	20	1

Figure 6 Forest plot of network meta-analysis results for prevention of delirium in trials where all participants received general anaesthesia

Table 5 Effect estimates for interventions in trials where all participants received general anaesthesia

Intervention	OR (95%CrI)	Trials	Participants
Diazepam+flunitrazepam+pethidine	0.07 (0.00-0.73)	1	20
Insulin (intranasal)	0.13 (0.04-0.38)	3	105
Haloperidol+ketamine	0.21 (0.03-1.11)	1	46
Aprotinin	0.22 (0.01-3.33)	1	18
Methylene blue	0.23 (0.05-0.99)	1	124
Thiamine	0.25 (0.04-1.43)	1	48
Dexmedetomidine (tracheal)	0.25 (0.05-1.16)	1	50
Risperidone	0.25 (0.05-1.17)	1	63
Dexmedetomidine+atorvastatin	0.26 (0.01-2.87)	1	20
Parecoxib	0.27 (0.05-1.57)	1	40
Cyproheptadine	0.29 (0.04-2.09)	1	23
Caffeine	0.30 (0.06-1.49)	1	34
Ketofol	0.33 (0.03-2.15)	1	40
Propofol+acetaminophen	0.34 (0.07-1.58)	1	31
Flurbiprofen	0.39 (0.08-1.91)	1	60
Ciprofol	0.40 (0.05-2.86)	1	53
Dexmedetomidine	0.45 (0.35-0.57)	62	6,050
Rivastigime	0.48 (0.19-1.15)	3	138
Ondansetron	0.49 (0.11-2.14)	1	51
Rosuvastatin	0.51 (0.13-2.08)	1	410
Hydrogen	0.53 (0.12-2.30)	1	85
Lidocaine	0.53 (0.20-1.40)	3	116
Haloperidol	0.53 (0.25-1.11)	4	382
Corticosteroids	0.55 (0.27-1.07)	5	4,283
Melatonin receptor agonists	0.55 (0.31-0.99)	7	379
Ketamine	0.57 (0.33-0.97)	11	780
Remifentanil	0.71 (0.18-3.05)	2	232

Intervention	OR (95%CrI)	Trials	Participants
Nitrous oxide	0.77 (0.19-2.75)	2	177
Morphine	0.85 (0.19-3.79)	1	152
Selenium	0.86 (0.24-3.11)	1	697
Clonidine	0.98 (0.25-4.13)	1	142
Magnesium	1.07 (0.09-14.93)	1	31
Xenon	1.13 (0.39-3.53)	4	266
Propofol	1.13 (0.60-2.26)	23	2,610
Morphine+midazolam	1.14 (0.06-39.31)	1	35
Tryptophan	1.16 (0.29-4.26)	1	152
Volatile	1.27 (0.60-2.90)	14	2,001
Physostigime	1.45 (0.36-5.67)	1	136
Remimazolam	1.47 (0.32-6.74)	2	221
Alprazolam	1.53 (0.38-6.25)	1	40
Atropine	1.57 (0.25-10.28)	1	30
Midazolam	1.75 (0.43-7.67)	1	40
Minocycline	1.83 (0.29-12.59)	1	100
Penehyclidine	6.32 (1.11-35.48)	1	30

OR, Odds Ratio; 95%CrI, 95% Credible Interval

Regional anaesthesia only

In these trials no participants had general anaesthesia.

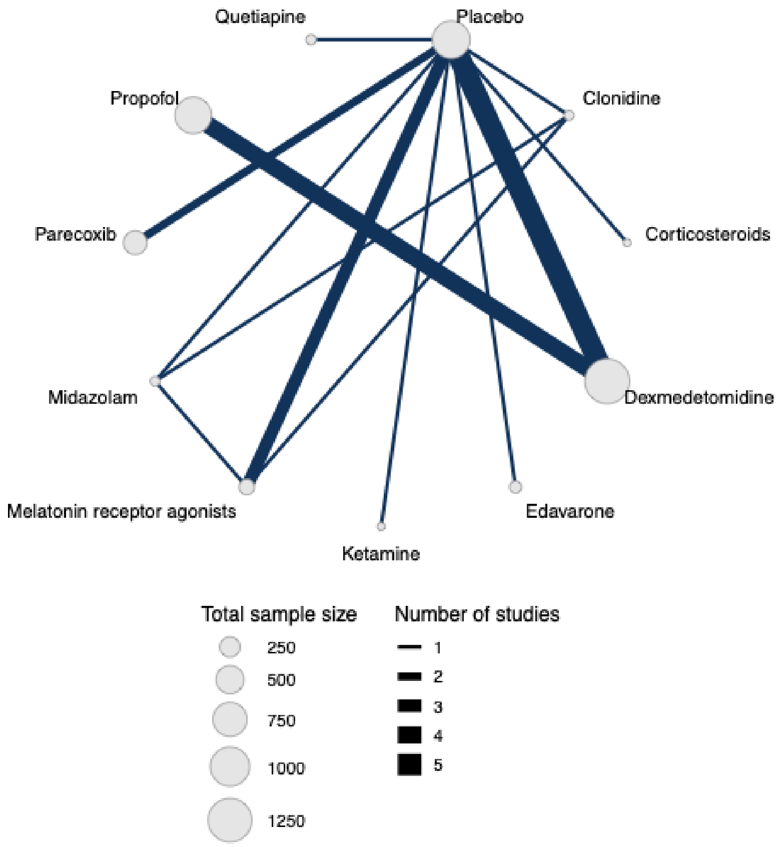

Figure 7 Network graph depicting the connectedness of the network for trials in which no participant received general anaesthesia

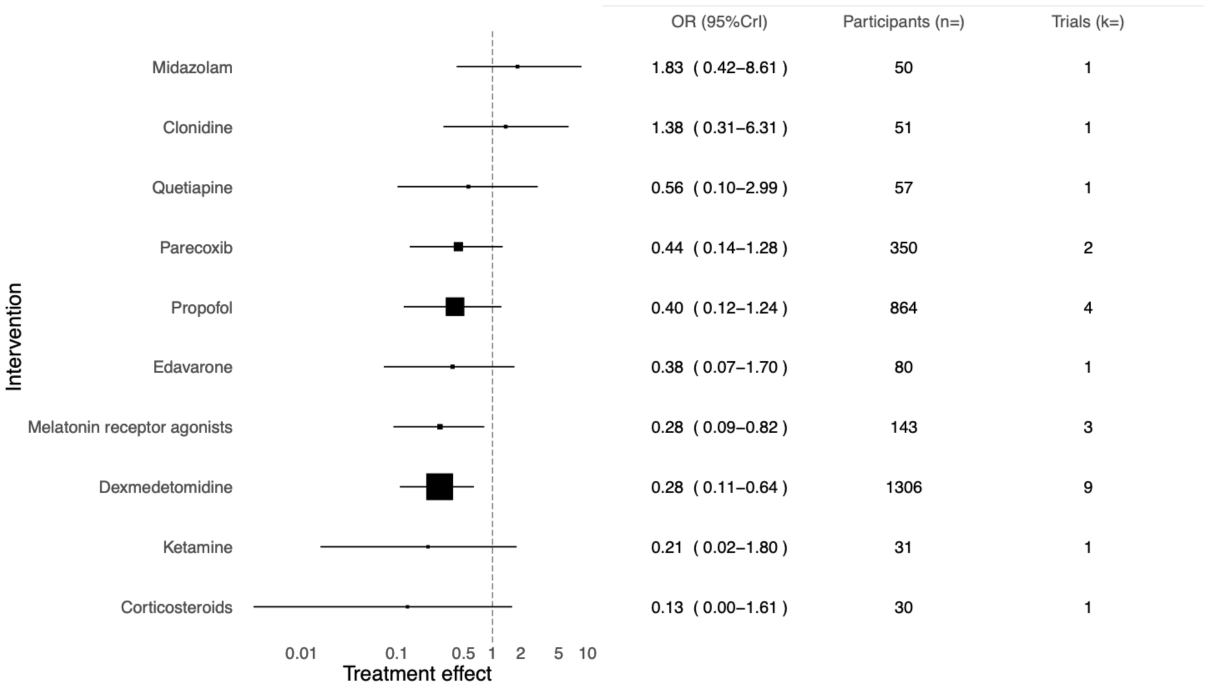

Figure 8 Forest plot of network meta-analysis results for prevention of delirium in trials where no participant received general anaesthesia

Table 6 Effect estimates for interventions in trials where no participant received general anaesthesia

Intervention	OR (95%CrI)	Trials	Participants
Corticosteroids	0.13 (0.00-1.61)	1	30
Ketamine	0.21 (0.02-1.80)	1	31
Dexmedetomidine	0.28 (0.11-0.64)	9	1,306
Melatonin receptor agonists	0.28 (0.09-0.82)	3	143
Edavarone	0.38 (0.07-1.70)	1	80
Propofol	0.40 (0.12-1.24)	4	864
Parecoxib	0.44 (0.14-1.28)	2	350
Quetiapine	0.56 (0.10-2.99)	1	57
Clonidine	1.38 (0.31-6.31)	1	51
Midazolam	1.83 (0.42-8.61)	1	50

OR, Odds Ratio; 95%CrI, 95% Credible Interval

Trials using general or regional anaesthesia

In these trials some participants had general anaesthesia and others had regional anaesthesia

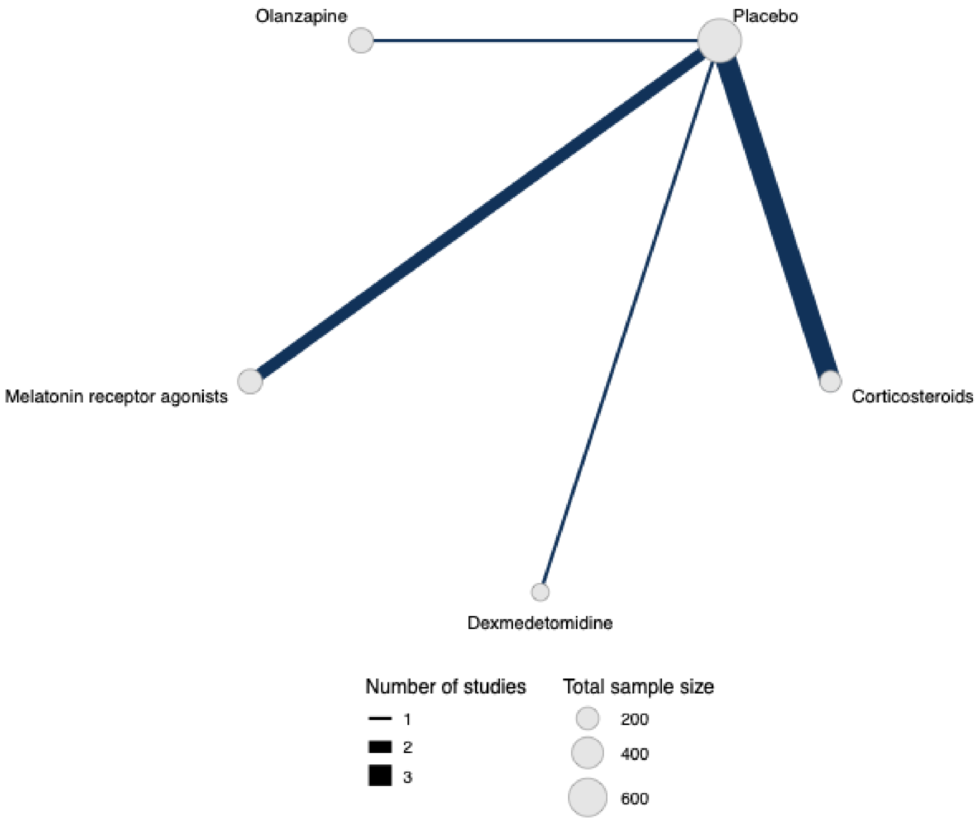

Figure 9 Network graph depicting the connectedness of the network for trials which included participants receiving general or regional anaesthesia

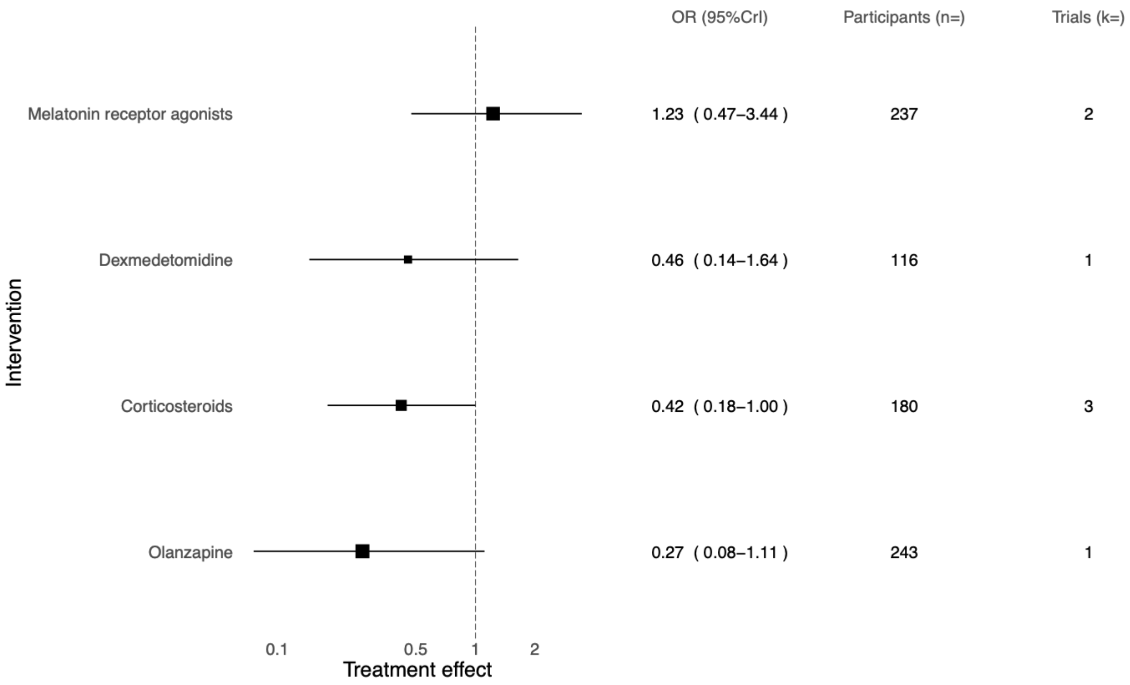

Figure 10 Forest plot of network meta-analysis results for prevention of delirium in trials which included participants receiving general or regional anaesthesia

Table 7 Effect estimates for interventions in trials which included participants receiving general or regional anaesthesia

	mixed anaesthetics subgroup		
Intervention	OR (95%CrI)	Trials	Participants
Olanzapine	0.27 (0.08-1.11)	1	243
Corticosteroids	0.42 (0.18-1.00)	3	180
Dexmedetomidine	0.46 (0.14-1.64)	1	116
Melatonin receptor agonists	1.23 (0.47-3.44)	2	237

OR, Odds Ratio; 95%CrI, 95% Credible Interval

Age of participants

At the request of a reviewer we conducted post hoc analysis of the interventions subgrouping trials into older adults (60-80 years) and very old adults (≥80 years). The mean participant age in 6.3% (n=10) trials was greater than 80 years of age, the remaining 148 trials had a mean participant age < 80 years.

Older adult trials (Age <80 years)

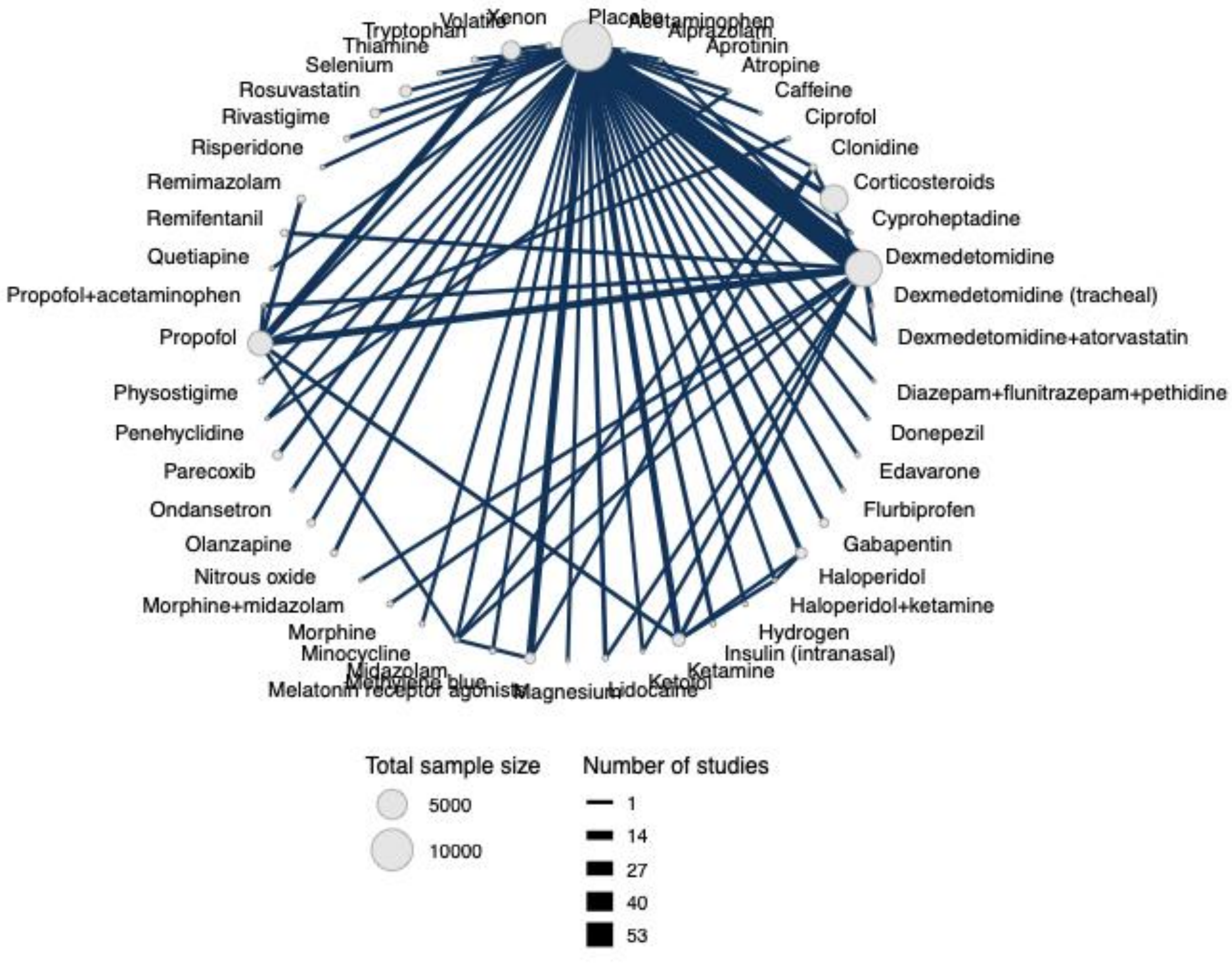

Figure 11 Network graph depicting the connectedness of the network for trials with a mean age of participants under 80 years of age

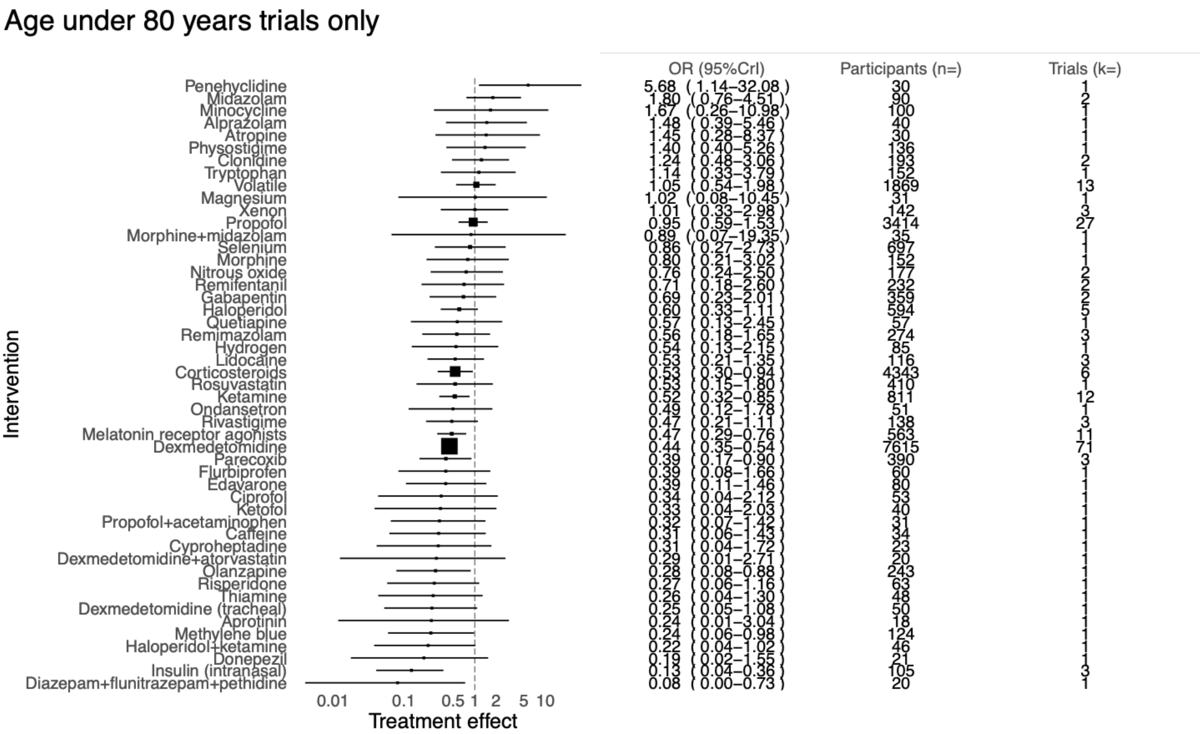

Figure 12 Forest plot of network meta-analysis results for prevention of delirium in trials with a mean age of participants under 80 years of age

Table 8 Effect estimates for interventions in trials with a mean age of participants under 80 years of age

	Age under 80 years only subgroup		
Intervention	OR (95%CrI)	Trials	Participants
Diazepam+flunitrazepam+pethidine	0.08 (0.00-0.73)	1	20
Insulin (intranasal)	0.13 (0.04-0.36)	3	105
Donepezil	0.19 (0.02-1.55)	1	21
Haloperidol+ketamine	0.22 (0.04-1.02)	1	46
Methylene blue	0.24 (0.06-0.98)	1	124
Aprotinin	0.24 (0.01-3.04)	1	18
Dexmedetomidine (tracheal)	0.25 (0.05-1.08)	1	50
Thiamine	0.26 (0.04-1.30)	1	48
Risperidone	0.27 (0.06-1.16)	1	63
Olanzapine	0.28 (0.08-0.88)	1	243
Dexmedetomidine+atorvastatin	0.29 (0.01-2.71)	1	20
Cyproheptadine	0.31 (0.04-1.72)	1	23

	Age under 80 years only subgroup		
Intervention	OR (95%CrI)	Trials	Participants
Caffeine	0.31 (0.06-1.43)	1	34
Propofol+acetaminophen	0.32 (0.07-1.42)	1	31
Ketofol	0.33 (0.04-2.03)	1	40
Ciprofol	0.34 (0.04-2.12)	1	53
Edavarone	0.39 (0.11-1.46)	1	80
Flurbiprofen	0.39 (0.08-1.66)	1	60
Parecoxib	0.39 (0.17-0.90)	3	390
Dexmedetomidine	0.44 (0.35-0.54)	71	7,615
Melatonin receptor agonists	0.47 (0.29-0.76)	11	563
Rivastigime	0.47 (0.21-1.11)	3	138
Ondansetron	0.49 (0.12-1.78)	1	51
Ketamine	0.52 (0.32-0.85)	12	811
Rosuvastatin	0.53 (0.15-1.80)	1	410
Corticosteroids	0.53 (0.30-0.94)	6	4,343
Lidocaine	0.53 (0.21-1.35)	3	116
Hydrogen	0.54 (0.13-2.15)	1	85
Remimazolam	0.56 (0.18-1.65)	3	274
Quetiapine	0.57 (0.13-2.45)	1	57
Haloperidol	0.60 (0.33-1.11)	5	594
Gabapentin	0.69 (0.23-2.01)	2	359
Remifentanil	0.71 (0.18-2.60)	2	232
Nitrous oxide	0.76 (0.24-2.50)	2	177
Morphine	0.80 (0.21-3.02)	1	152
Selenium	0.86 (0.27-2.73)	1	697
Morphine+midazolam	0.89 (0.07-19.35)	1	35
Propofol	0.95 (0.59-1.53)	27	3,414
Xenon	1.01 (0.33-2.98)	3	142
Magnesium	1.02 (0.08-10.45)	1	31

	Age under 80 years only subgroup		
Intervention	OR (95%CrI)	Trials	Participants
Volatile	1.05 (0.54-1.98)	13	1,869
Tryptophan	1.14 (0.33-3.79)	1	152
Clonidine	1.24 (0.48-3.06)	2	193
Physostigime	1.40 (0.40-5.26)	1	136
Atropine	1.45 (0.28-8.37)	1	30
Alprazolam	1.48 (0.39-5.46)	1	40
Minocycline	1.67 (0.26-10.98)	1	100
Midazolam	1.80 (0.76-4.51)	2	90
Penehyclidine	5.68 (1.14-32.08)	1	30

OR, Odds Ratio; 95%CrI, 95% Credible Interval

Very old age participant trials (Age ≥80 years)

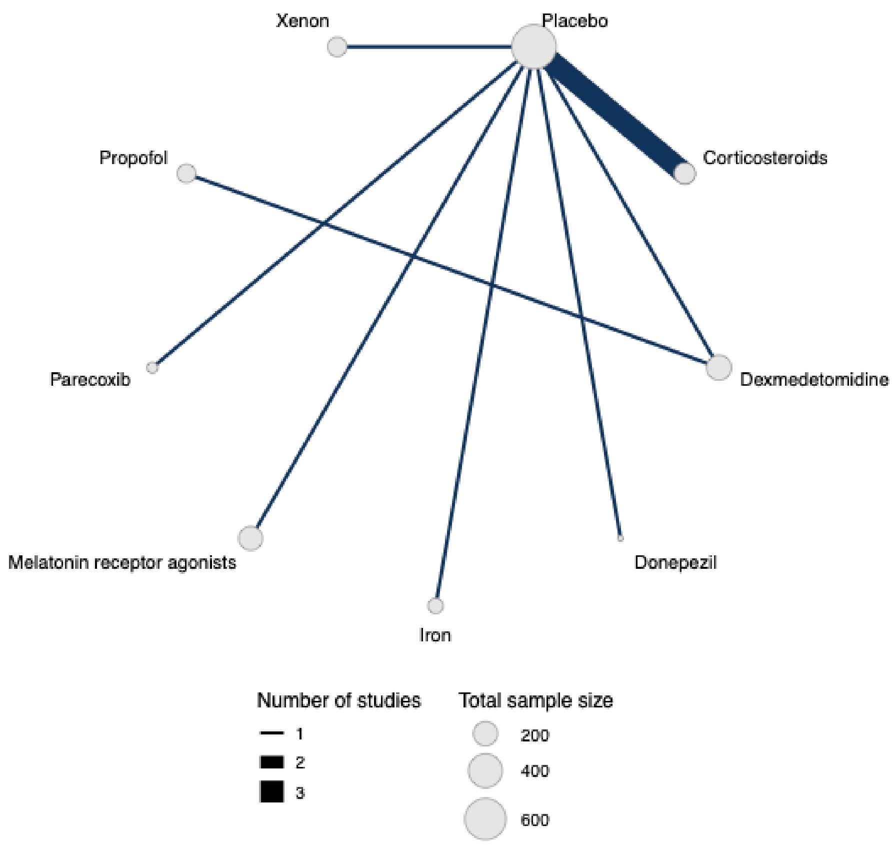

Figure 13 Network graph depicting the connectedness of the network for trials with a mean age of participants over 80 years of age

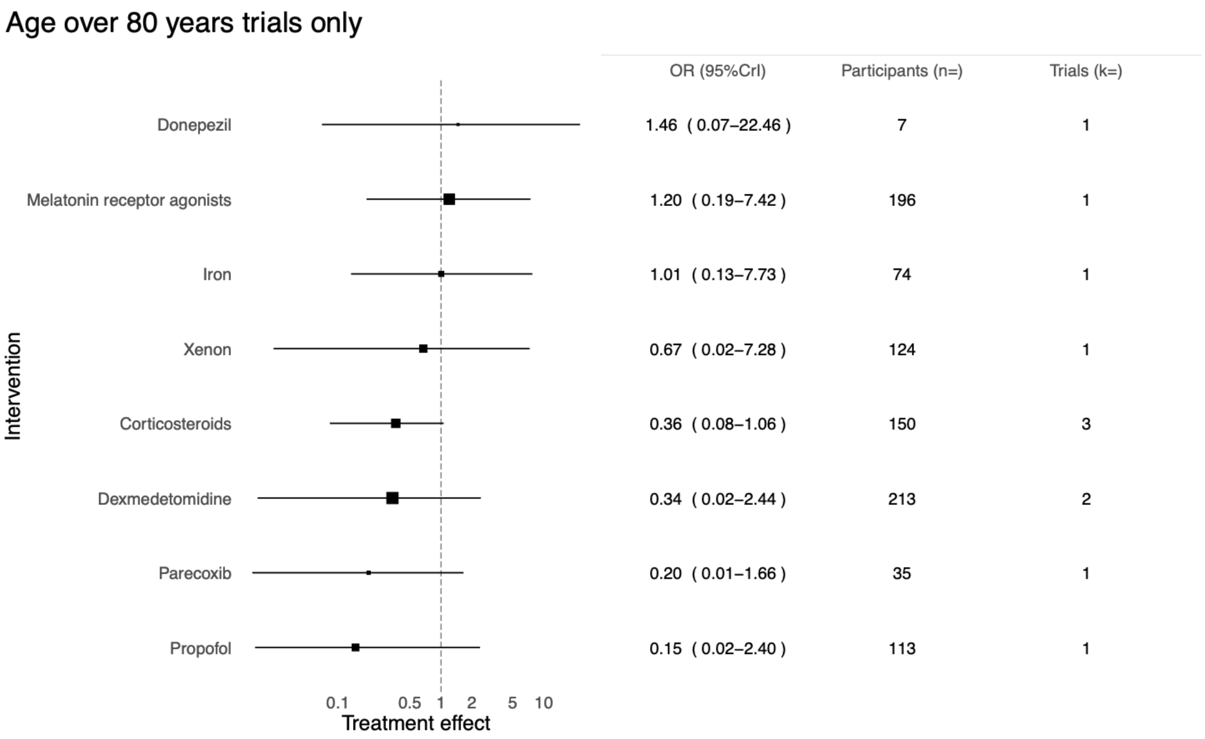

Figure 14 Forest plot of network meta-analysis results for prevention of delirium in trials with a mean age of participants over 80 years of age

Table 9 Effect estimates for interventions in trials with a mean age of participants over 80 years of age

	Age over 80 years only subgroup		
Intervention	OR (95%CrI)	Trials	Participants
Propofol	0.15 (0.02-2.40)	1	113
Parecoxib	0.20 (0.01-1.66)	1	35
Dexmedetomidine	0.34 (0.02-2.44)	2	213
Corticosteroids	0.36 (0.08-1.06)	3	150
Xenon	0.67 (0.02-7.28)	1	124
Iron	1.01 (0.13-7.73)	1	74
Melatonin receptor agonists	1.20 (0.19-7.42)	1	196
Donepezil	1.46 (0.07-22.46)	1	7

OR, Odds Ratio; 95%CrI, 95% Credible Interval

Section 7. Rankograms and SUCRA values

Cumulative ranking probabilities (‘Rankograms’) and Surface under the cumulative ranking area (SUCRA) values for prevention of delirium by prespecified subgroups. Surface under the cumulative ranking area (SUCRA) is the summary value of the ranking estimates of treatments compared across the network, ranging from 0 (worst) to 1 (best). It is reported with the median rank and 95% Credible Interval ranks.[20 21] It is the Bayesian equivalent to P-scores used in frequentist network meta-analyses.

95%CrI is the 95% credible interval.

All operations

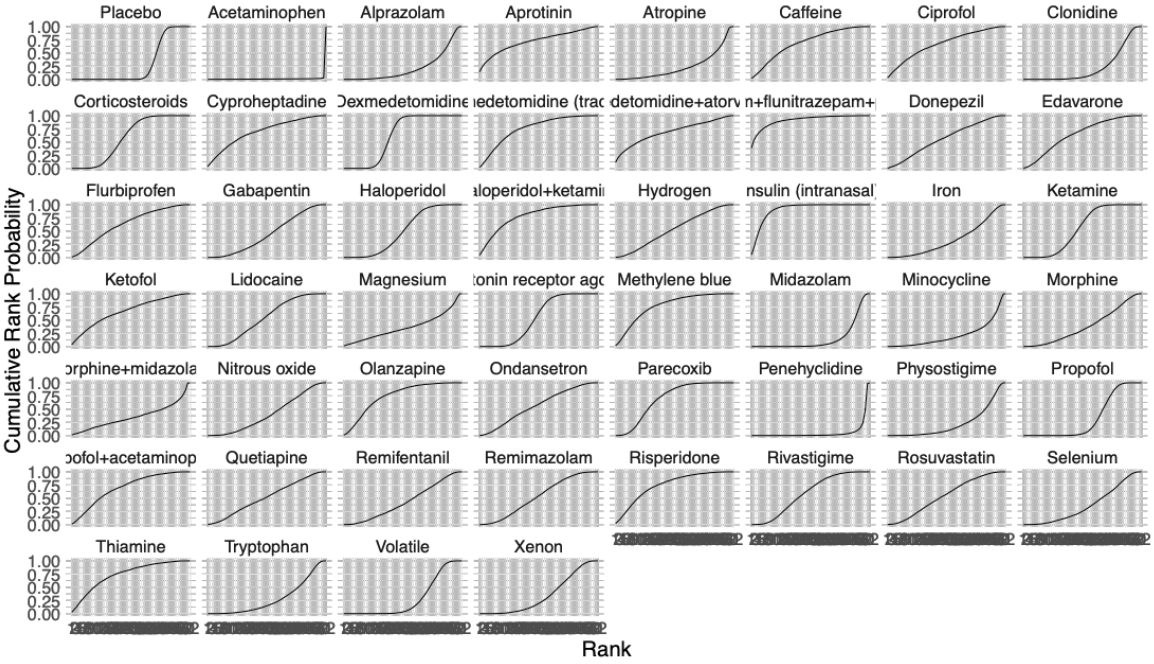

Figure 15 Cumulative ranking probabilities for all interventions across all trials

Table 10 Surface under the cumulative ranking values for global model (all interventions in all trials)

Intervention	SUCRA	Mean Rank (95% CrI)
Diazepam+flunitrazepam+ pethidine	0.912	5.51 (1-30)
Insulin (intranasal)	0.907	5.74 (1-16)
Haloperidol+ketamine	0.780	12.22 (1-39)
Methylene blue	0.765	12.99 (2-38)
Dexmedetomidine (tracheal)	0.751	13.68 (2-39)
Risperidone	0.744	14.08 (2-40.03)

Intervention	SUCRA	Mean Rank (95% CrI)
Olanzapine	0.742	14.13 (2-38)
Thiamine	0.735	14.52 (1-43)
Aprotinin	0.716	15.48 (1-49)
Propofol+acetaminophen	0.693	16.65 (2-43)
Cyproheptadine	0.692	16.70 (1-46)
Caffeine	0.690	16.82 (2-43)
Parecoxib	0.690	16.84 (6-33)
Dexmedetomidine+atorvas tatin	0.688	16.91 (1-48)
Ciprofol	0.666	18.02 (1-47)
Ketofol	0.665	18.10 (1-47)
Edavarone	0.640	19.37 (3-44)
Flurbiprofen	0.630	19.86 (3-46)
Dexmedetomidine	0.614	20.66 (14-27)
Rivastigime	0.581	22.35 (8-41)
Corticosteroids	0.577	22.59 (12-35)
Donepezil	0.558	23.52 (3-47)
Ondansetron	0.556	23.63 (4-47)
Ketamine	0.541	24.41 (13-35)
Rosuvastatin	0.539	24.53 (6-46)
Lidocaine	0.534	24.78 (8-43)
Melatonin receptor agonists	0.533	24.83 (14-36)
Hydrogen	0.524	25.25 (5-47)
Remimazolam	0.520	25.50 (7-45)
Quetiapine	0.511	25.92 (4-48)
Haloperidol	0.490	26.99 (13-40)
Gabapentin	0.437	29.72 (9-47)
Remifentanil	0.424	30.36 (7-49)
Nitrous oxide	0.404	31.41 (9-49)

Intervention	SUCRA	Mean Rank (95% CrI)
Morphine	0.378	32.71 (9-49)
Magnesium	0.360	33.65 (3-51)
Selenium	0.358	33.77 (11-49)
Morphine+midazolam	0.349	34.18 (2-51)
Xenon	0.340	34.64 (15-48)
Iron	0.329	35.21 (10-50)
Propofol	0.319	35.71 (26-44)
Placebo	0.276	37.91 (33-43)
Tryptophan	0.275	37.98 (14.98-50)
Volatile	0.274	38.01 (26-47)
Atropine	0.234	40.09 (12-50)
Physostigime	0.220	40.77 (17.98-51)
Clonidine	0.218	40.89 (25-49)
Alprazolam	0.214	41.08 (17-51)
Minocycline	0.207	41.42 (12-51)
Midazolam	0.144	44.65 (32-50)
Penehyclidine	0.047	49.61 (41-51)
Acetaminophen	0.007	51.66 (51-52)

Hip fracture surgery

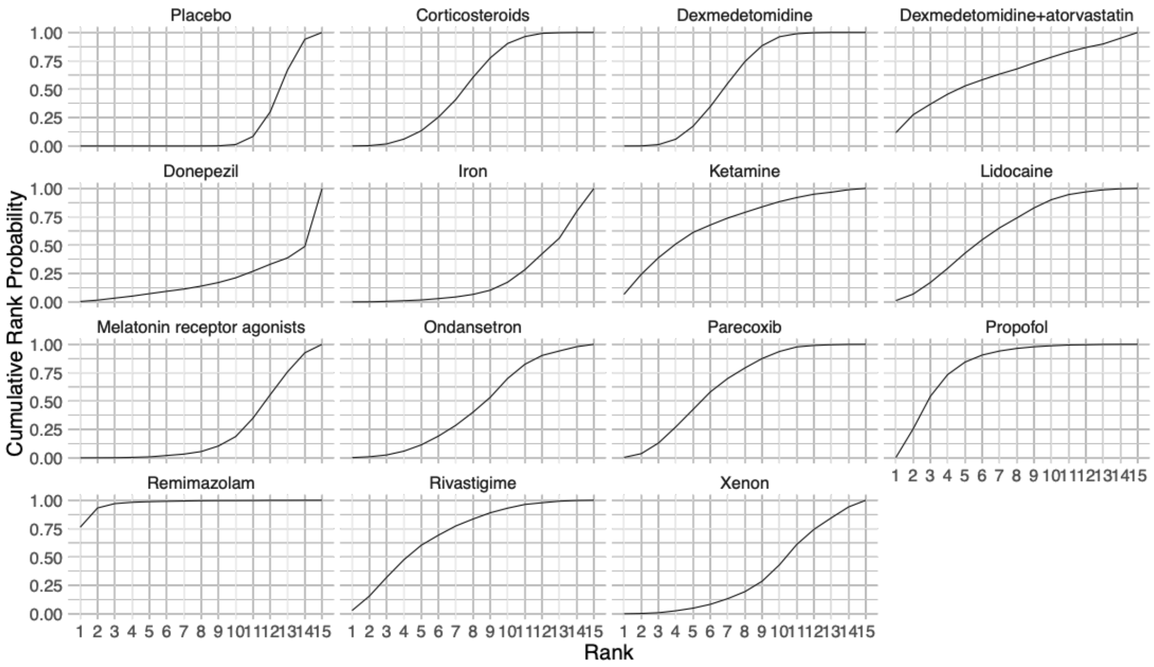

Figure 16 Cumulative ranking probabilities for interventions in hip fracture surgery

Table 11 Surface under the cumulative ranking values for hip fracture surgery

Intervention	SUCRA	Mean Rank (95% CrI)
Remimazolam	0.972	1.40 (1-4)
Propofol	0.796	3.86 (2-9)
Rivastigime	0.688	5.36 (1-12)
Ketamine	0.684	5.43 (1-14)
Parecoxib	0.623	6.28 (2-11)
Dexmedetomidine+atorvastatin	0.621	6.30 (1-15)
Lidocaine	0.610	6.46 (2-13)
Dexmedetomidine	0.552	7.28 (4-11)
Corticosteroids	0.508	7.88 (4-12)
Ondansetron	0.427	9.03 (3-14)
Xenon	0.311	10.64 (4-15)
Melatonin receptor agonists	0.215	11.99 (7-15)
Iron	0.180	12.48 (6-15)

Intervention	SUCRA	Mean Rank (95% CrI)
Donepezil	0.170	12.62 (3-15)
Placebo	0.144	12.99 (11-15)

Cardiac surgery

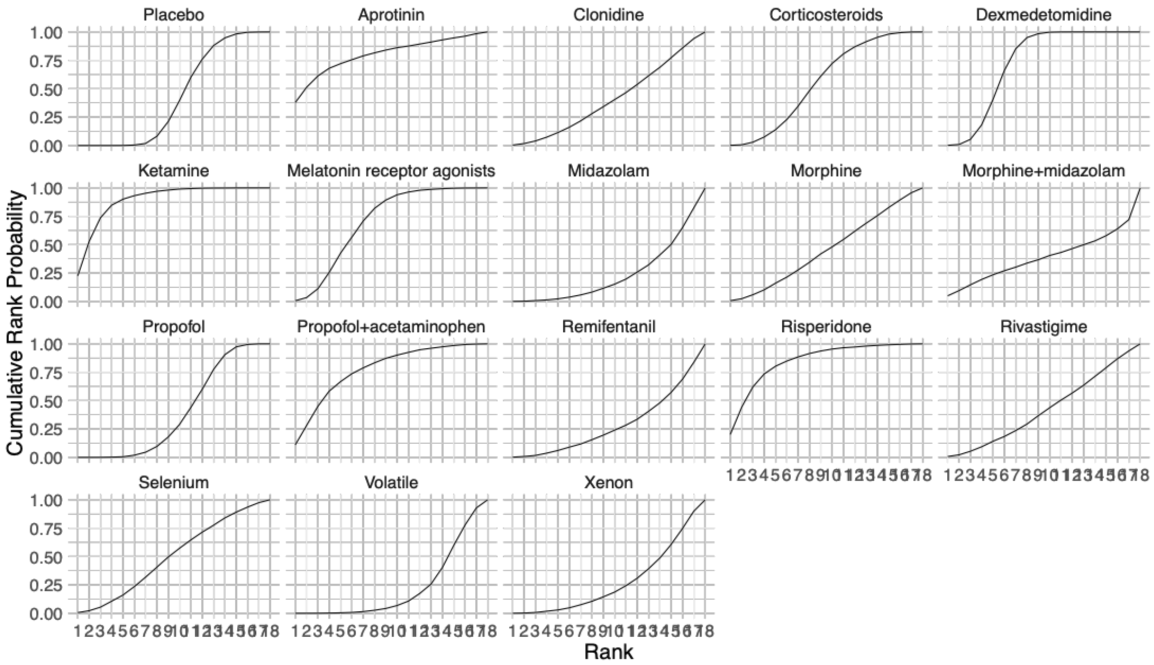

Figure 17 Cumulative ranking probabilities for interventions in cardiac surgery

Table 12 Surface under the cumulative ranking values for cardiac surgery

Intervention	SUCRA	Mean Rank (95% CrI)
Ketamine	0.885	2.95 (1-9)
Risperidone	0.837	3.77 (1-13)
Aprotinin	0.792	4.53 (1-17)
Propofol+acetaminophen	0.765	5.00 (1-15)
Dexmedetomidine	0.712	5.90 (3-9)
Melatonin receptor agonists	0.687	6.33 (2-12)
Corticosteroids	0.539	8.84 (3-15)
Selenium	0.480	9.84 (3-18)
Morphine	0.434	10.62 (3-18)
Placebo	0.405	11.12 (8-15)
Rivastigime	0.403	11.15 (3-18)
Clonidine	0.384	11.46 (3-18)
Propofol	0.372	11.67 (7-16)
Morphine+midazolam	0.369	11.73 (1-18)

Intervention	SUCRA	Mean Rank (95% CrI)
Remifentanil	0.266	13.47 (4-18)
Xenon	0.253	13.69 (5-18)
Midazolam	0.215	14.34 (6-18)
Volatile	0.201	14.59 (8-18)

Non cardiac surgery

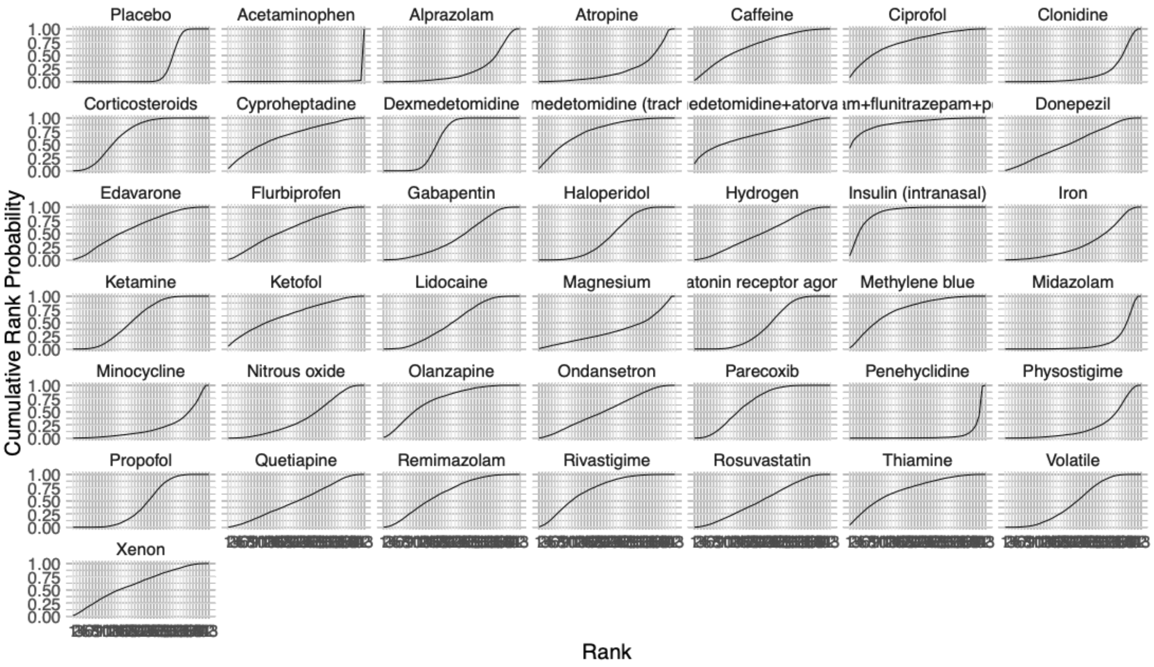

Figure 18 Cumulative ranking probabilities for interventions in non-cardiac surgery

Table 13 Surface under the cumulative ranking values for non-cardiac surgery

Intervention	SUCRA	Mean Rank (95% CrI)
Diazepam+flunitrazepam+ pethidine	0.896	5.36 (1-29)
Insulin (intranasal)	0.895	5.39 (1-17)
Dexmedetomidine (tracheal)	0.749	11.54 (1-33)
Ciprofol	0.741	11.86 (1-37)
Methylene blue	0.741	11.89 (2-33)
Olanzapine	0.714	12.99 (2-33)
Thiamine	0.712	13.09 (1-36)
Rivastigime	0.709	13.21 (2-31)
Corticosteroids	0.682	14.36 (5-27)
Dexmedetomidine+atorvas tatin	0.679	14.49 (1-40)
Caffeine	0.675	14.65 (2-37)
Cyproheptadine	0.667	15.00 (1-38)
Parecoxib	0.651	15.65 (5-29)

Intervention	SUCRA	Mean Rank (95% CrI)
Ketofol	0.649	15.74 (1-38)
Remimazolam	0.630	16.55 (4-35)
Xenon	0.608	17.47 (2-38)
Edavarone	0.607	17.49 (3-37)
Dexmedetomidine	0.597	17.92 (12-24)
Flurbiprofen	0.595	18.02 (2-38)
Ketamine	0.561	19.43 (8-31)
Donepezil	0.530	20.73 (3-39)
Ondansetron	0.522	21.07 (3-39)
Rosuvastatin	0.506	21.75 (5-38)
Hydrogen	0.505	21.79 (4-39)
Lidocaine	0.494	22.27 (8-36)
Quetiapine	0.493	22.28 (3-39)
Volatile	0.469	23.31 (9.98-35)
Haloperidol	0.451	24.07 (11-35)
Propofol	0.442	24.44 (13-34)
Melatonin receptor agonists	0.436	24.68 (13-35)
Gabapentin	0.418	25.43 (7-38)
Nitrous oxide	0.378	27.13 (8-40)
Magnesium	0.341	28.68 (3-42)
Iron	0.310	29.99 (9-41)
Placebo	0.254	32.34 (28-36)
Atropine	0.218	33.83 (11-41)
Physostigime	0.217	33.87 (14-42)
Alprazolam	0.211	34.13 (15-42)
Minocycline	0.205	34.39 (11-42)
Clonidine	0.161	36.24 (22-42)
Midazolam	0.120	37.95 (27-42)
Penehyclidine	0.053	40.77 (34-42)

Intervention	SUCRA	Mean Rank (95% CrI)
Acetaminophen	0.007	42.72 (42-43)

Abdominal surgery

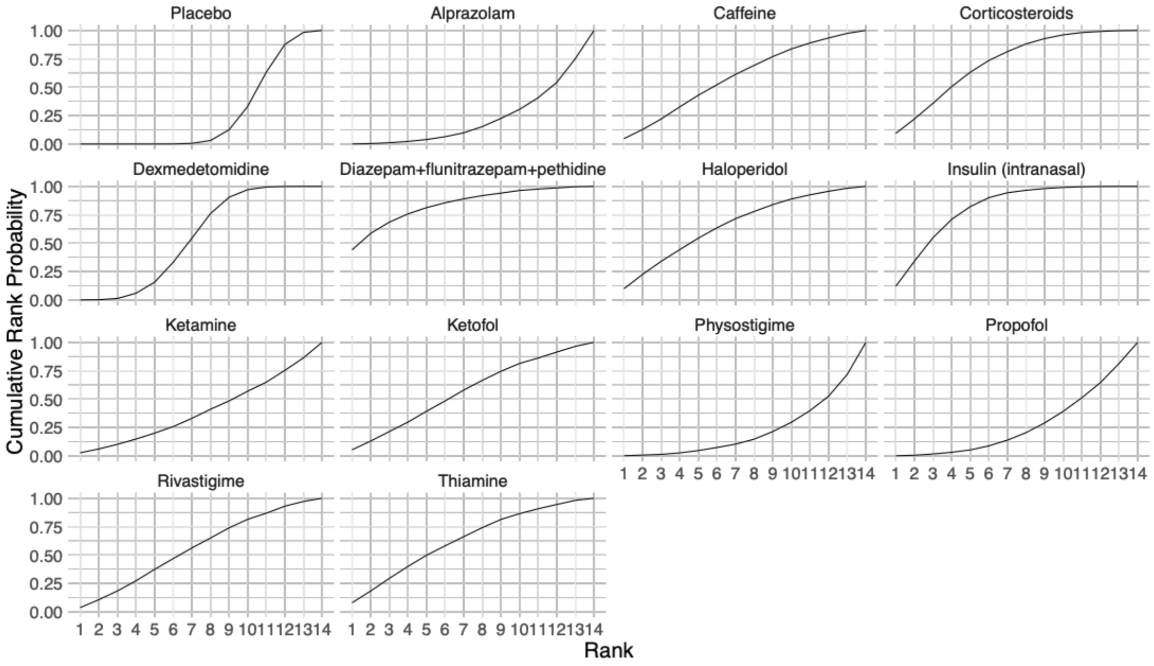

Figure 19 Cumulative ranking probabilities for interventions in abdominal surgery

Table 14 Surface under the cumulative ranking values for abdominal surgery

Intervention	SUCRA	Mean Rank (95% CrI)
Diazepam+flunitrazepam+ pethidine	0.832	3.19 (1-11)
Insulin (intranasal)	0.794	3.68 (1-9)
Corticosteroids	0.700	4.90 (1-11)
Haloperidol	0.644	5.62 (1-13)
Thiamine	0.612	6.05 (1-13)
Caffeine	0.568	6.62 (1-14)
Ketofol	0.547	6.88 (1-14)
Rivastigime	0.537	7.02 (1-14)
Dexmedetomidine	0.518	7.27 (4-11)
Ketamine	0.374	9.14 (1-14)
Propofol	0.246	10.81 (4-14)
Placebo	0.230	11.01 (8-13)
Alprazolam	0.202	11.38 (5-14)
Physostigime	0.198	11.43 (4-14)

Orthopaedic surgery

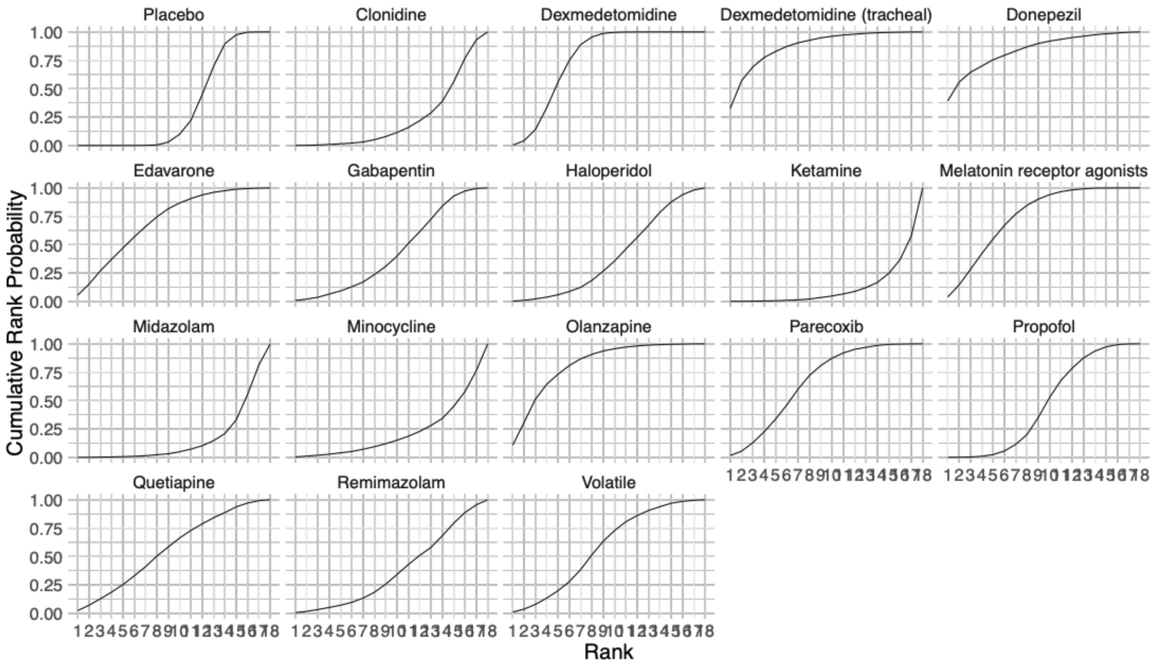

Figure 20 Cumulative ranking probabilities for interventions in orthopaedic surgery

Table 15 Surface under the cumulative ranking values for orthopaedic surgery

Intervention	SUCRA	Mean Rank (95% CrI)
Dexmedetomidine (tracheal)	0.867	3.26 (1-12)
Donepezil	0.833	3.84 (1-14)
Olanzapine	0.806	4.29 (1-12)
Dexmedetomidine	0.744	5.34 (2-9)
Melatonin receptor agonists	0.735	5.50 (1-12)
Edavarone	0.691	6.26 (1-14)
Parecoxib	0.650	6.95 (2-14)
Volatile	0.557	8.53 (2-16)
Quetiapine	0.547	8.69 (2-17)
Propofol	0.443	10.47 (6-16)
Gabapentin	0.415	10.94 (3-17)
Haloperidol	0.379	11.57 (4-17)
Remimazolam	0.355	11.96 (3-18)

Intervention	SUCRA	Mean Rank (95% CrI)
Placebo	0.316	12.62 (9-15.03)
Clonidine	0.215	14.35 (7-18)
Minocycline	0.202	14.57 (4-18)
Midazolam	0.139	15.63 (9-18)
Ketamine	0.105	16.22 (9-18)

Thoracic Surgery

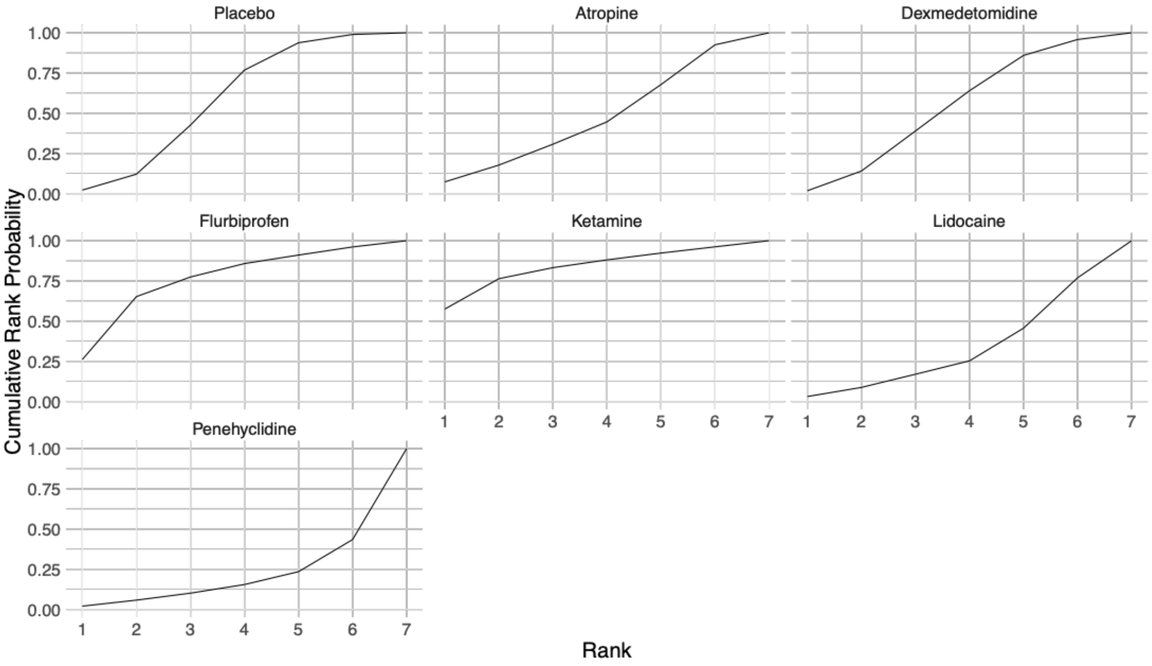

Figure 21 Cumulative ranking probabilities for interventions in thoracic surgery

Table 16 Surface under the cumulative ranking values for thoracic surgery

Intervention	SUCRA	Mean Rank (95% CrI)
Ketamine	0.823	2.06 (1-7)
Flurbiprofen	0.736	2.58 (1-7)
Placebo	0.544	3.74 (2-6)
Dexmedetomidine	0.501	4.00 (2-7)
Atropine	0.434	4.39 (1-7)
Lidocaine	0.294	5.23 (1-7)
Penehyclidine	0.167	6.00 (2-7)

Elective Surgery

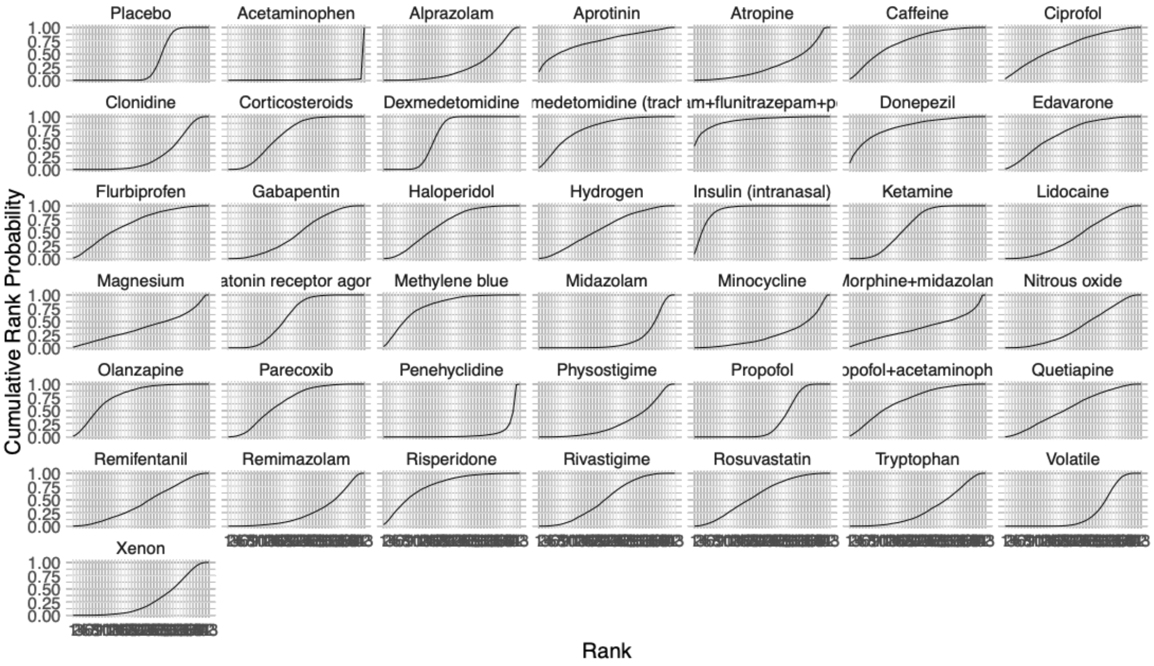

Figure 22 Cumulative ranking probabilities for interventions studied in trials in the emergency surgical setting

Table 17 Surface under the cumulative ranking values for trials of both elective surgical setting

Intervention	SUCRA	Mean Rank (95% CrI)
Diazepam+flunitrazepam+ pethidine	0.916	4.52 (1-25)
Insulin (intranasal)	0.916	4.55 (1-12)
Donepezil	0.800	9.40 (1-35)
Methylene blue	0.790	9.80 (2-27)
Olanzapine	0.770	10.64 (2-27)
Dexmedetomidine (tracheal)	0.769	10.70 (1-30.03)
Risperidone	0.765	10.89 (1-31)
Aprotinin	0.733	12.23 (1-40)
Caffeine	0.719	12.79 (2-34)
Propofol+acetaminophen	0.692	13.95 (2-35)
Edavarone	0.669	14.90 (2-35)
Flurbiprofen	0.658	15.38 (2-36)
Parecoxib	0.651	15.66 (5-32)
Corticosteroids	0.650	15.71 (6-28)

Intervention	SUCRA	Mean Rank (95% CrI)
Ciprofol	0.643	16.00 (2-38)
Haloperidol	0.633	16.42 (4-33)
Dexmedetomidine	0.624	16.78 (12-22)
Ketamine	0.605	17.60 (8-28)
Melatonin receptor agonists	0.582	18.55 (9-29)
Rosuvastatin	0.574	18.88 (4-36)
Hydrogen	0.566	19.24 (4-38)
Quetiapine	0.547	20.04 (4-39)
Rivastigime	0.506	21.75 (8-36)
Gabapentin	0.467	23.40 (7-38)
Remifentanil	0.436	24.68 (6-40)
Lidocaine	0.425	25.17 (8-39)
Nitrous oxide	0.385	26.81 (9-40)
Magnesium	0.385	26.82 (2-42)
Morphine+midazolam	0.376	27.23 (2-42)
Placebo	0.334	28.96 (24-34)
Tryptophan	0.313	29.87 (12-41)
Propofol	0.305	30.21 (23-36)
Xenon	0.286	31.00 (15-41)
Atropine	0.269	31.72 (9-41)
Physostigime	0.262	32.01 (13-41)
Volatile	0.260	32.08 (22.98-39)
Remimazolam	0.258	32.18 (12-42)
Clonidine	0.254	32.32 (19-40)
Minocycline	0.242	32.86 (9-42)
Alprazolam	0.241	32.86 (14-42)
Midazolam	0.162	36.19 (25-42)
Penehyclidine	0.059	40.52 (32-42)
Acetaminophen	0.006	42.74 (43-43)

Emergency Surgery

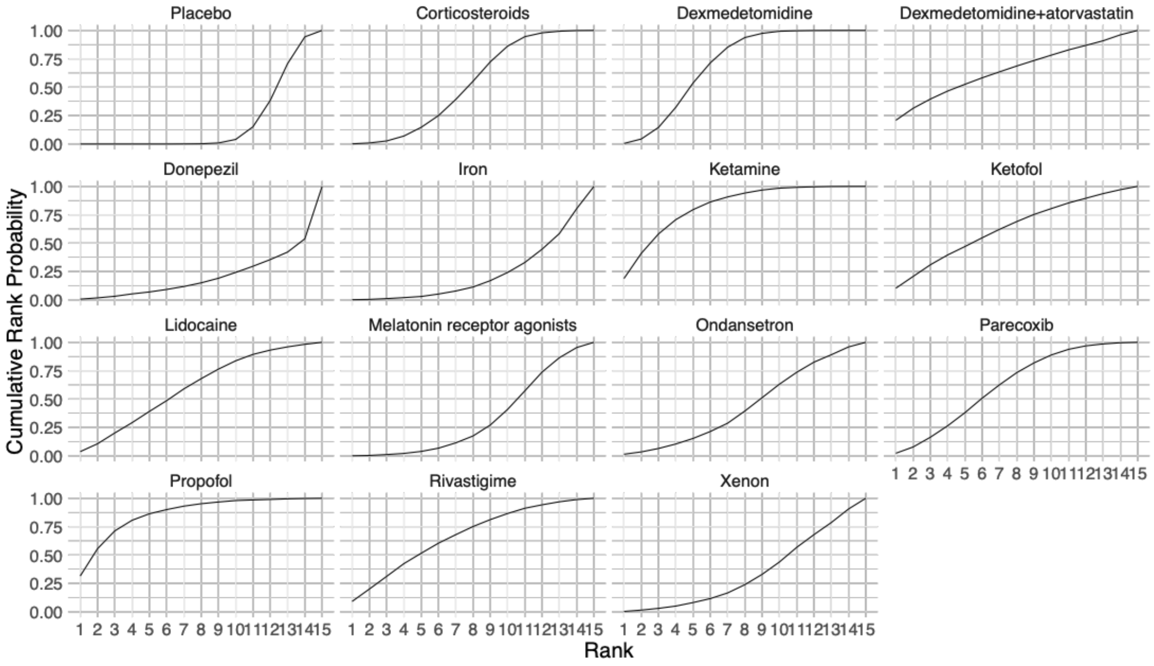

Figure 23 Cumulative ranking probabilities for interventions studied in trials in the emergency surgical setting

Table 18 Surface under the cumulative ranking values for trials in the emergency surgical setting

Intervention	SUCRA	Mean Rank (95% CrI)
Propofol	0.853	3.05 (1-10)
Ketamine	0.809	3.67 (1-10)
Dexmedetomidine	0.680	5.48 (2-10)
Rivastigime	0.648	5.93 (1-14)
Dexmedetomidine+atorvastatin	0.636	6.10 (1-15)
Ketofol	0.611	6.44 (1-15)
Parecoxib	0.598	6.63 (2-13)
Lidocaine	0.582	6.85 (1-14)
Corticosteroids	0.496	8.05 (3-12)
Ondansetron	0.416	9.17 (2-15)
Xenon	0.315	10.59 (3-15)
Melatonin receptor agonists	0.303	10.76 (5-15)
Iron	0.206	12.11 (5-15)

Intervention	SUCRA	Mean Rank (95% CrI)
Donepezil	0.185	12.41 (3-15)
Placebo	0.160	12.76 (10-15)

Mixed urgency of surgery (elective and emergency)

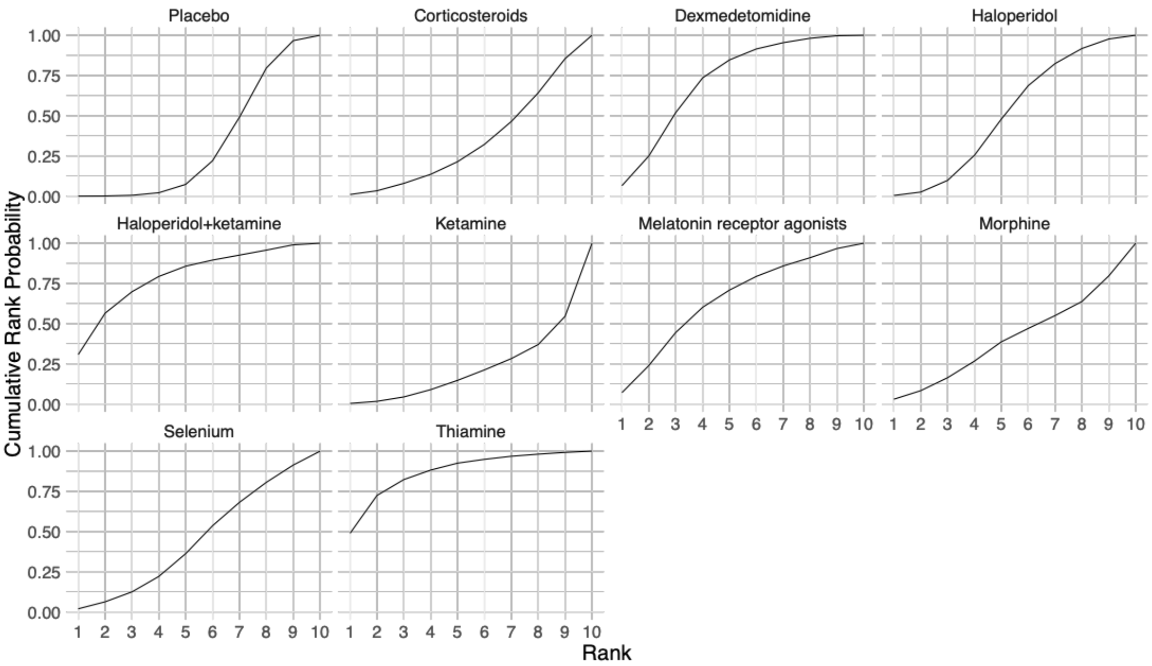

Figure 24 Cumulative ranking probabilities for interventions studied in trials of both elective and emergency surgical settings

Table 19 Surface under the cumulative ranking values for trials of both elective and emergency surgical settings

Intervention	SUCRA	Mean Rank (95% CrI)
Thiamine	0.859	2.26 (1-8)
Haloperidol+ketamine	0.777	3.01 (1-9)
Dexmedetomidine	0.696	3.74 (1-8)
Melatonin receptor agonists	0.622	4.41 (1-10)
Haloperidol	0.474	5.73 (2.98-9)
Selenium	0.414	6.27 (2-10)
Morphine	0.376	6.62 (1-10)
Corticosteroids	0.306	7.25 (2-10)
Placebo	0.286	7.43 (5-10)
Ketamine	0.190	8.29 (3-10)

Excluding high risk of bias trials

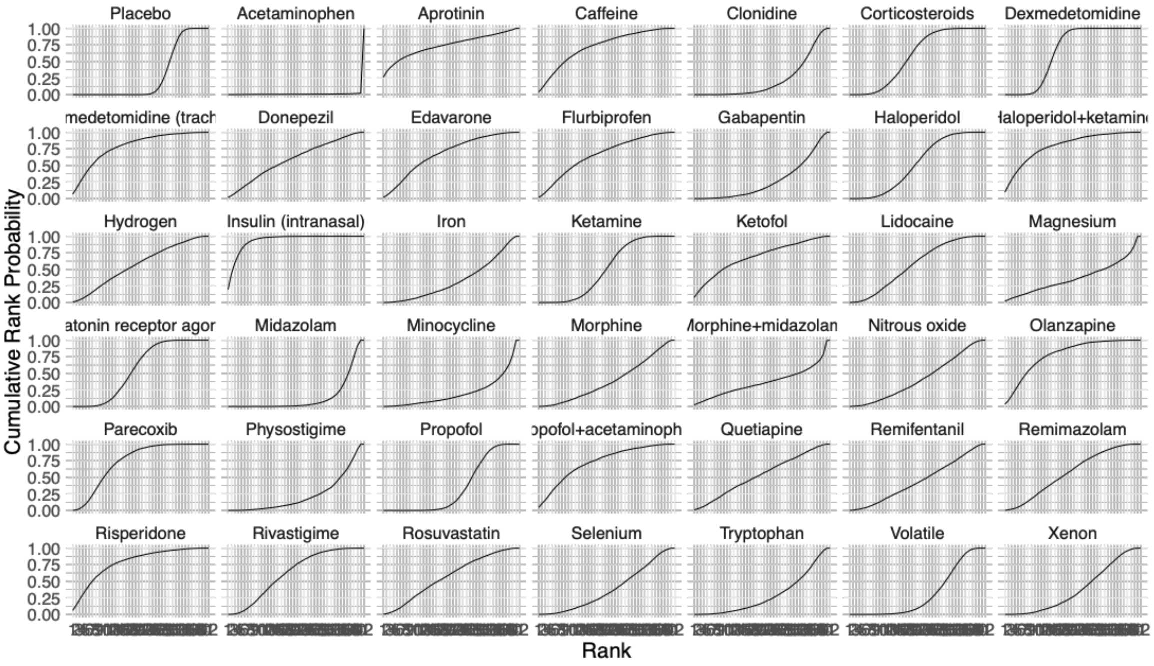

Figure 25 Cumulative ranking probabilities for interventions excluding trials at high risk of bias

Table 20 Surface under the cumulative ranking values excluding trials at high risk of bias

Intervention	SUCRA	Mean Rank (95% CrI)
Insulin (intranasal)	0.932	3.78 (1-12)
Haloperidol+ketamine	0.791	9.56 (1-33)
Dexmedetomidine (tracheal)	0.783	9.89 (1-32)
Risperidone	0.778	10.11 (1-33)
Olanzapine	0.777	10.14 (1-29)
Aprotinin	0.737	11.78 (1-40)
Parecoxib	0.731	12.03 (4-26)
Propofol+acetaminophen	0.723	12.36 (1-35)
Caffeine	0.720	12.47 (1-36)
Ketofol	0.682	14.04 (1-39)
Edavarone	0.671	14.50 (2-36)
Flurbiprofen	0.657	15.05 (2-37)
Dexmedetomidine	0.646	15.52 (10-21)
Rivastigime	0.614	16.84 (5-33)

Intervention	SUCRA	Mean Rank (95% CrI)
Donepezil	0.586	17.99 (2-39)
Corticosteroids	0.570	18.65 (9-29)
Hydrogen	0.564	18.87 (3-38)
Rosuvastatin	0.564	18.89 (3-38)
Remimazolam	0.562	18.95 (4-36)
Melatonin receptor agonists	0.559	19.10 (10-28.03)
Lidocaine	0.558	19.13 (5-35)
Quetiapine	0.528	20.37 (3-39.03)
Haloperidol	0.509	21.12 (9-33)
Ketamine	0.495	21.71 (11-32)
Remifentanil	0.435	24.16 (5-40)
Nitrous oxide	0.409	25.21 (6-40)
Morphine	0.381	26.39 (6-40)
Selenium	0.377	26.56 (7-40)
Xenon	0.371	26.77 (10-39)
Magnesium	0.368	26.93 (2-41)
Morphine+midazolam	0.360	27.24 (2-41)
Propofol	0.341	28.02 (19-36)
Iron	0.330	28.48 (7-41)
Volatile	0.300	29.72 (17-38)
Tryptophan	0.282	30.43 (10-41)
Placebo	0.276	30.67 (26-35)
Gabapentin	0.260	31.33 (12-41)
Physostigime	0.222	32.89 (12-41)
Clonidine	0.216	33.13 (18-41)
Minocycline	0.201	33.76 (9-41)
Midazolam	0.128	36.74 (26-41)
Acetaminophen	0.007	41.73 (42-42)

Excluding sparse trial data

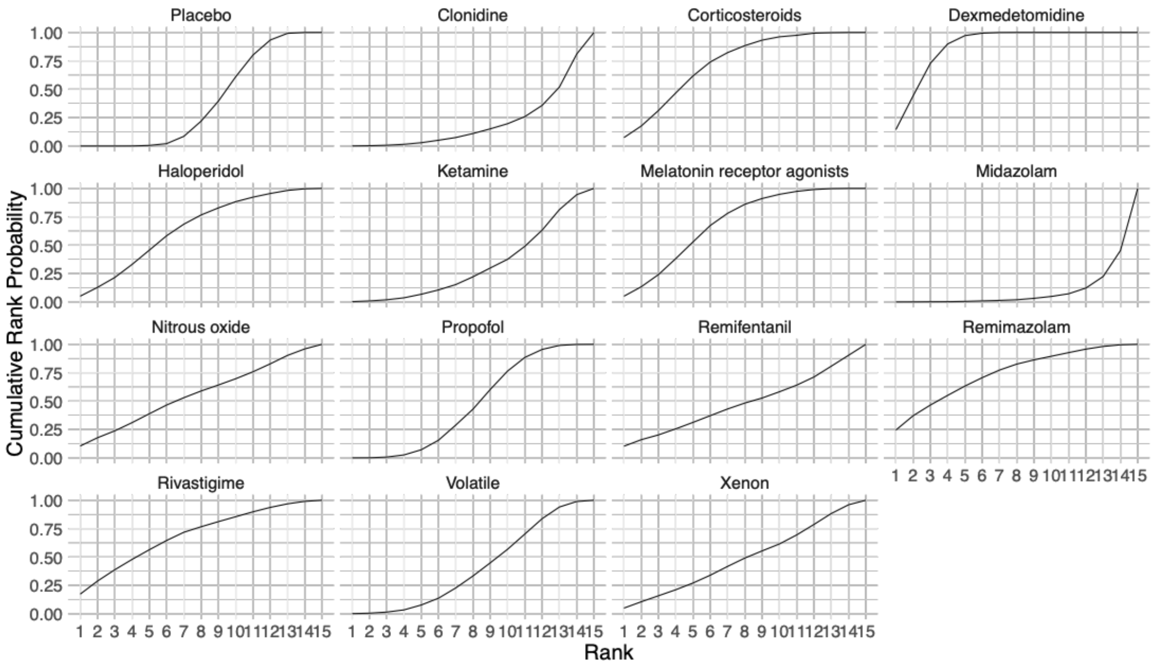

Figure 26 Cumulative ranking probabilities for interventions studied in more than one trial and trials of at least 100 participants

Table 21 Surface under the cumulative ranking values excluding sparse trial data

Intervention	SUCRA	Mean Rank (95% CrI)
Dexmedetomidine	0.870	2.82 (1-6)
Remimazolam	0.728	4.81 (1-13)
Corticosteroids	0.711	5.04 (1-11.03)
Rivastigime	0.677	5.52 (1-14)
Melatonin receptor agonists	0.677	5.53 (1-12)
Haloperidol	0.627	6.22 (1-13)
Nitrous oxide	0.542	7.41 (1-15)
Xenon	0.467	8.46 (1-15)
Remifentanil	0.464	8.51 (1-15)
Propofol	0.441	8.83 (4-13)
Volatile	0.380	9.68 (4-14)
Placebo	0.362	9.94 (7-13)
Ketamine	0.298	10.83 (4-15)

Intervention	SUCRA	Mean Rank (95% CrI)
Clonidine	0.184	12.42 (5-15)
Midazolam	0.072	14.00 (9-15)

Section 8. Inconsistency checking

Global Model - Residual deviance

Fixed effects model
Residual deviance: 565.2 (on 337 data points)
pD: 211.5
DIC: 776.8

Random effects model
Residual deviance: 358.0 (on 337 data points)
pD: 278.0
DIC: 636.1

Unrelated mean effects model
Residual deviance: 355.9 (on 337 data points)
pD: 280.4
DIC: 636.3

Node splitting

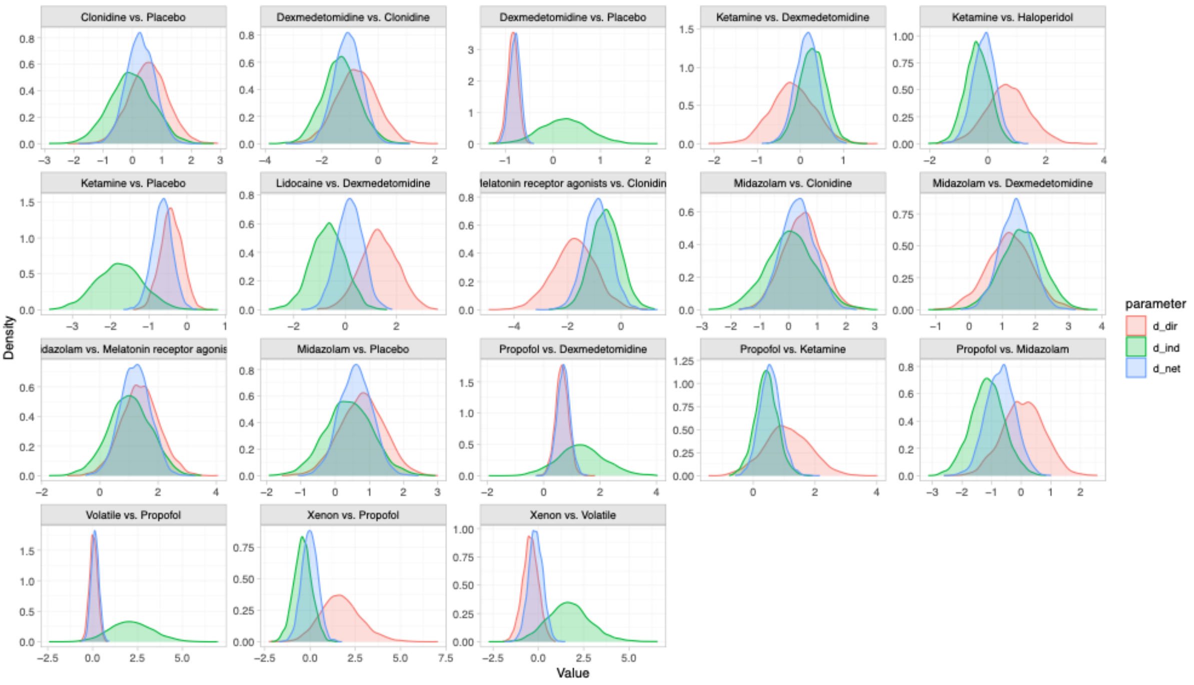

Figure 27 Posterior distributions for direct, indirect and network estimates assessing loop inconsistency

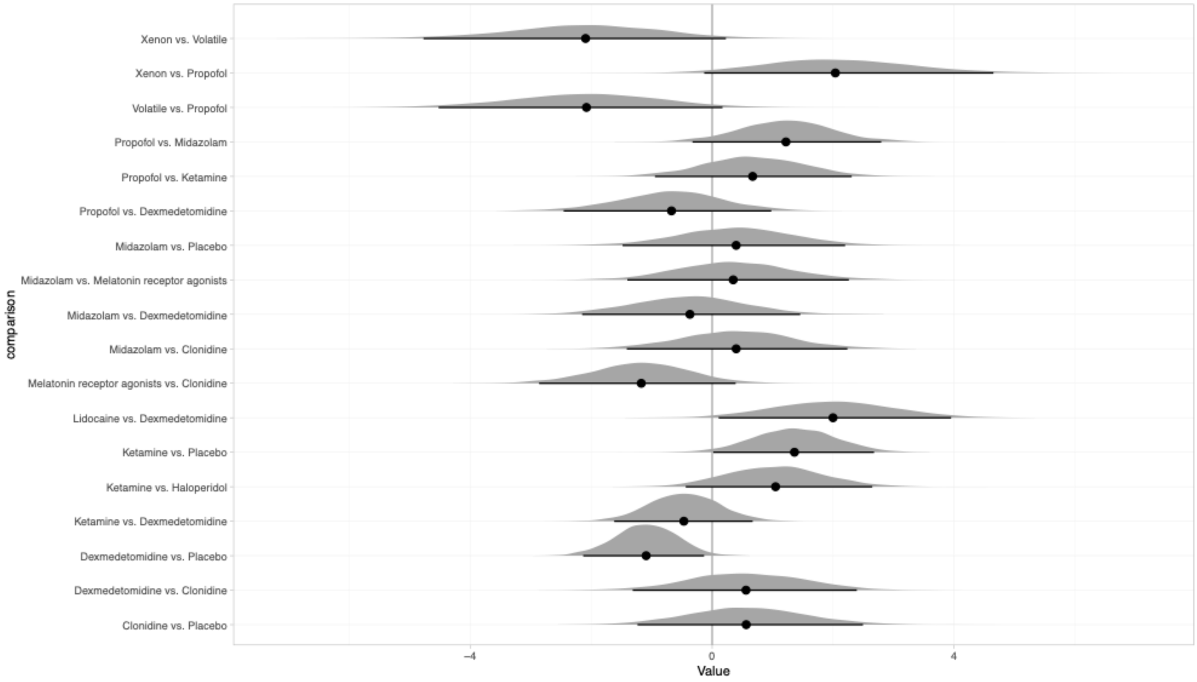

Figure 28 Posterior distributions for the inconsistency factors

Section 9. Individual Risk of Bias assessments

Using Cochrane Risk of Bias tool version 2. Visualization using *robvis* (https://www.riskofbias.info/welcome/robvis-visualization-tool).[22]

See Supplementary file 3

Section 10. CINeMA quality assessments

Implementation of CINeMA framework

The certainty of evidence was assessed using the Confidence in Network Meta-Analysis (CINeMA) framework.[23] This approach has been specifically developed from the GRADE framework for use in network meta-analyses. We assessed confidence in the network estimates for the primary outcome of delirium prevention across all operations.

Within-study bias: Quality of evidence was down rated for interventions where the weighted average across studies contributing evidence were assessed as high risk of bias using the Cochrane Risk of Bias tool version 2.0.
Reporting bias: Publication bias was analysed with visual inspection of funnel plots. The paucity of interventions studied in 10 or more trials.
Indirectness: To mitigate against violation of transitivity assumptions our prespecified inclusion and exclusion criteria explicitly set out to capture studies specifically addressing our primary research question: 'Which pharmacologic interventions are effective at preventing postoperative delirium in adults over 60 years old?'. Secondly, we pre-specified major surgical categories (specialties and urgency) from which heterogeneity of treatment effect may arise. Consequently we *a priori* reduced the indirectness of the evidence we set out to synthesis. Many drugs were subject to only one trial or only ever compared to one intervention. Therefore, interventions forming single connected nodes in the network (only compared to one other intervention across the whole network) were down rated due to the increased reliance of the estimate of treatment effect through comparison via only one other intervention.
Imprecision: We set the threshold for a clinically significant thresholds for the estimated treatment effects to be an odds ratio below 0.952 or above 1.05.
Heterogeneity: per CINeMA standard as described by Papakonstantinou and colleagues[24]
Incoherence: per CINeMA standard as described by Papakonstantinou and colleagues
Overall assessment[24]
The overall confidence assessment of the certainty of evidence is rated high, moderate, low or very low. The ratings are based on the composite of the six CINeMA domains assessed above. Comparisons where there were major concerns in two or more domains and some concerns in at least one other domain were rated very low confidence. Comparisons with major concerns in one domain were rated low confidence. Absent any major concerns confidence was rated moderate. In the event there were no concerns or some concerns in two or fewer domains confidence would be rated high. The closely correlated domains of indirectness and incoherence, and the domains imprecision and heterogeneity were considered jointly when down rating certainty as recommended by Salanti and colleagues.[25]

Credibility assessments (CINeMA framework)

Table 22 Confidence in the quality of the evidence using the CINeMA framework

Comparison	k	Within-study bias	Reporting bias	Indirectness	Imprecision	Heterogeneity	Incoherence	Confidence rating	Reason(s) for downgrading
Mixed (direct and indirect evidence)									
Insulin (intranasal):Placebo	2	Some concerns	Some concerns	Some concerns	No concerns	No concerns	Some concerns	Moderate	["Indirectness"]
Acetaminophen:Placebo	1	Some concerns	Some concerns	No concerns	Major concerns	No concerns	Some concerns	Low	["Within-study bias","Imprecision"]
Aprotinin:Placebo	1	Some concerns	Some concerns	Some concerns	Major concerns	No concerns	Some concerns	Low	["Within-study bias","Imprecision"]
Caffeine:Placebo	1	No concerns	Some concerns	Some concerns	Major concerns	No concerns	Some concerns	Low	["Imprecision"]
Clonidine:Placebo	1	Some concerns	Some concerns	No concerns	Major concerns	No concerns	No concerns	Low	["Within-study bias","Imprecision"]
Corticosteroids:Placebo	9	No concerns	Some concerns	Some concerns	No concerns	Major concerns	Some concerns	Low	["Indirectness","Heterogeneity"]
Dexmedetomidine:Placebo	50	Some concerns	Some concerns	No concerns	No concerns	Major concerns	No concerns	Low	["Reporting bias","Heterogeneity"]

Donepezil:Placebo	2	Some concerns	Some concerns	Some concerns	Major concerns	No concerns	Some concerns	Low	["Indirectness","Imprecision"]
Edavarone:Placebo	1	Some concerns	Some concerns	Some concerns	Major concerns	No concerns	Some concerns	Low	["Indirectness","Imprecision"]
Flurbiprofen:Placebo	1	Some concerns	Some concerns	Some concerns	Major concerns	No concerns	Some concerns	Low	["Indirectness","Imprecision"]
Gabapentin:Placebo	2	No concerns	Some concerns	Some concerns	Major concerns	No concerns	Some concerns	Low	["Indirectness","Imprecision"]
Haloperidol:Placebo	5	Some concerns	Some concerns	No concerns	Major concerns	No concerns	No concerns	Low	["Within-study bias","Imprecision"]
Haloperidol+ketamine:Placebo	1	No concerns	Some concerns	No concerns	Major concerns	No concerns	No concerns	Low	["Reporting bias","Imprecision"]
Iron:Placebo	1	No concerns	Some concerns	Some concerns	Major concerns	No concerns	Some concerns	Low	["Indirectness","Imprecision"]
Ketamine:Placebo	8	Some concerns	Some concerns	No concerns	No concerns	Major concerns	No concerns	Low	["Within-study bias","Heterogeneity"]
Ketofol:Placebo	1	Some concerns	Some concerns	No concerns	Major concerns	No concerns	No concerns	Low	["Imprecision"]
Lidocaine:Placebo	3	Some concerns	Some concerns	No concerns	Major concerns	No concerns	No concerns	Low	["Imprecision"]
Magnesium:Placebo	1	No concerns	Some concerns	Some concerns	Major concerns	No concerns	Some concerns	Low	["Imprecision"]
Melatonin receptor agonists:Placebo	12	Some concerns	Low risk	No concerns	No concerns	Major concerns	No concerns	Low	["Heterogeneity"]
Midazolam:Placebo	1	Some	Some	No	Major	No	No concerns	Low	["Imprecisio

		concerns	concerns	concerns	concerns	concerns			n"]
Minocycline:Placebo	1	No concerns	Some concerns	Some concerns	Major concerns	No concerns	Some concerns	Low	["Indirectness","Imprecision"]
Nitrous oxide:Placebo	2	Some concerns	Some concerns	Some concerns	Major concerns	No concerns	Some concerns	Low	["Indirectness","Imprecision"]
Olanzapine:Placebo	1	Some concerns	Some concerns	Some concerns	No concerns	Major concerns	Some concerns	Low	["Indirectness","Heterogeneity"]
Parecoxib:Placebo	4	Some concerns	Some concerns	Some concerns	No concerns	Major concerns	Some concerns	Low	["Indirectness","Heterogeneity"]
Physostigime:Placebo	1	No concerns	Some concerns	Some concerns	Major concerns	No concerns	Some concerns	Low	["Indirectness","Imprecision"]
Placebo:Quetiapine	1	Some concerns	Some concerns	Some concerns	Major concerns	No concerns	Some concerns	Low	["Indirectness","Imprecision"]
Placebo:Risperidone	1	Some concerns	Some concerns	Some concerns	Some concerns	Some concerns	Some concerns	Low	["Indirectness","Imprecision"]
Placebo:Rivastigime	3	Some concerns	Some concerns	Some concerns	Major concerns	No concerns	Some concerns	Low	["Indirectness","Imprecision"]
Placebo:Rosuvastatin	1	Some concerns	Some concerns	Some concerns	Major concerns	No concerns	Some concerns	Low	["Indirectness","Imprecision"]
Placebo:Selenium	1	Some concerns	Some concerns	Some concerns	Major concerns	No concerns	Some concerns	Low	["Indirectness","Imprecision"]
Placebo:Tryptophan	1	No concerns	Some concerns	Some concerns	Major concerns	No concerns	Some concerns	Low	["Indirectness","Imprecision"]
Alprazolam:Placebo	1	Major	Some	No	Major	No	No concerns	Very low	["Within-

		concerns	concerns	concerns	concerns	concerns			study bias","Imprecision"]
Atropine:Placebo	1	Major concerns	Some concerns	No concerns	Major concerns	No concerns	Some concerns	Very low	["Within-study bias","Imprecision"]
Cyproheptadine:Placebo	1	Major concerns	Some concerns	Some concerns	Major concerns	No concerns	Some concerns	Very low	["Within-study bias","Indirectness","Imprecision"]
Dexmedetomidine+atorvastatin:Placebo	1	Major concerns	Some concerns	No concerns	Major concerns	No concerns	No concerns	Very low	["Within-study bias","Reporting bias","Imprecision"]
Diazepam+flunitrazepam+pethidine:Placebo	1	Major concerns	Some concerns	Some concerns	Major concerns	No concerns	Some concerns	Very low	["Within-study bias","Reporting bias","Imprecision"]
Hydrogen:Placebo	1	Some concerns	Some concerns	Some concerns	Major concerns	No concerns	Some concerns	Very low	["Within-study bias","Reporting bias","Indirectness","Imprecision"]
Methylene blue:Placebo	1	Major concerns	Some concerns	Some concerns	No concerns	Major concerns	Some concerns	Very low	["Within-study bias","Indirectness","Het

									erogeneity"]
Ondansetron:Placebo	1	Major concerns	Some concerns	Some concerns	Major concerns	No concerns	Some concerns	Very low	["Within-study bias","Indirectness","Imprecision"]
Penehyclidine:Placebo	1	Major concerns	Some concerns	No concerns	No concerns	Major concerns	Some concerns	Very low	["Within-study bias","Reporting bias","Heterogeneity"]
Placebo:Thiamine	1	Major concerns	Some concerns	Some concerns	Major concerns	No concerns	Some concerns	Very low	["Within-study bias","Indirectness","Imprecision"]
Indirect evidence									
Ciprofol:Placebo	0	Some concerns	Some concerns	No concerns	Major concerns	No concerns	Some concerns	Low	["Within-study bias","Reporting bias","Imprecision"]
Dexmedetomidine (tracheal):Placebo	0	No concerns	Some concerns	No concerns	Major concerns	No concerns	Some concerns	Low	["Reporting bias","Imprecision"]
Morphine:Placebo	0	Some concerns	Some concerns	No concerns	Major concerns	No concerns	Some concerns	Low	["Within-study bias","Reporting bias","Imprecision"]
Morphine+midazolam:Placebo	0	Some concerns	Some concerns	No concerns	Major concerns	No concerns	Some concerns	Low	["Within-study

									bias","Reporting bias","Imprecision"]
Placebo:Propofol	0	Some concerns	Low risk	No concerns	Major concerns	No concerns	Some concerns	Low	["Imprecision"]
Placebo:Propofol+acetaminophen	0	Some concerns	Some concerns	No concerns	Major concerns	No concerns	Some concerns	Low	["Within-study bias","Reporting bias","Imprecision"]
Placebo:Remifentanil	0	Some concerns	Some concerns	No concerns	Major concerns	No concerns	Some concerns	Low	["Within-study bias","Reporting bias","Imprecision"]
Placebo:Remimazolam	0	Some concerns	Some concerns	No concerns	Major concerns	No concerns	Some concerns	Low	["Within-study bias","Reporting bias","Imprecision"]
Placebo:Volatile	0	Some concerns	Some concerns	No concerns	Major concerns	No concerns	Some concerns	Low	["Within-study bias","Reporting bias","Imprecision"]
Placebo:Xenon	0	Some concerns	Some concerns	No concerns	Major concerns	No concerns	Some concerns	Low	["Within-study bias","Reporting bias","Impre

cision"]

Section 11. Pairwise comparisons

League table of all pairwise comparisons for primary outcome

See Supplementary file 5 for full table.
Odds ratios and their 95% confidence intervals are presented in the table. Odds ratios <1 favour the intervention specified in the row.

Section 12. Secondary Outcomes

Mortality

Mortality was reported for 66 trials. One only reported 12-month mortality, and another only reported 3-month mortality, limiting comparison with other trials. There were 64 trials with comparable mortality outcomes reported. 39 trials reported the in-hospital mortality rate, a further 25 reported mortality between 28 days–1month after surgery.

Nine trials reported zero events across all comparisons in their study for this outcome (mortality) were Cyproheptadine, Flurbiprofen, Hydrogen, Lidocaine, Methylene blue, Midazolam, Parecoxib, Propofol+acetaminophen,and Remimazolam and thus were excluded from mortality analysis. The trials were Maldonado 2009, Mohammadi 2016, Mu 2017, Subramaniam 2019, Deng 2021, Shen 2022, Lin 2022, Jeon 2023, and Gajniak 2023.

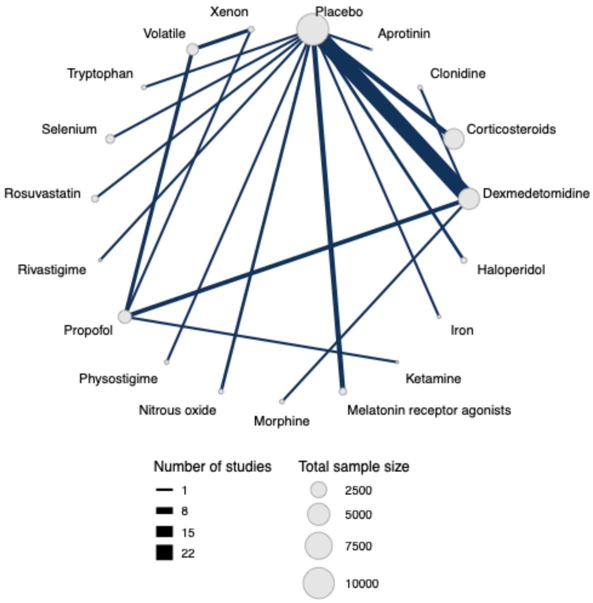

Figure 29 Network graph depicting the connectedness of the network for delirium prevention trials reporting mortality within 1 month of surgery

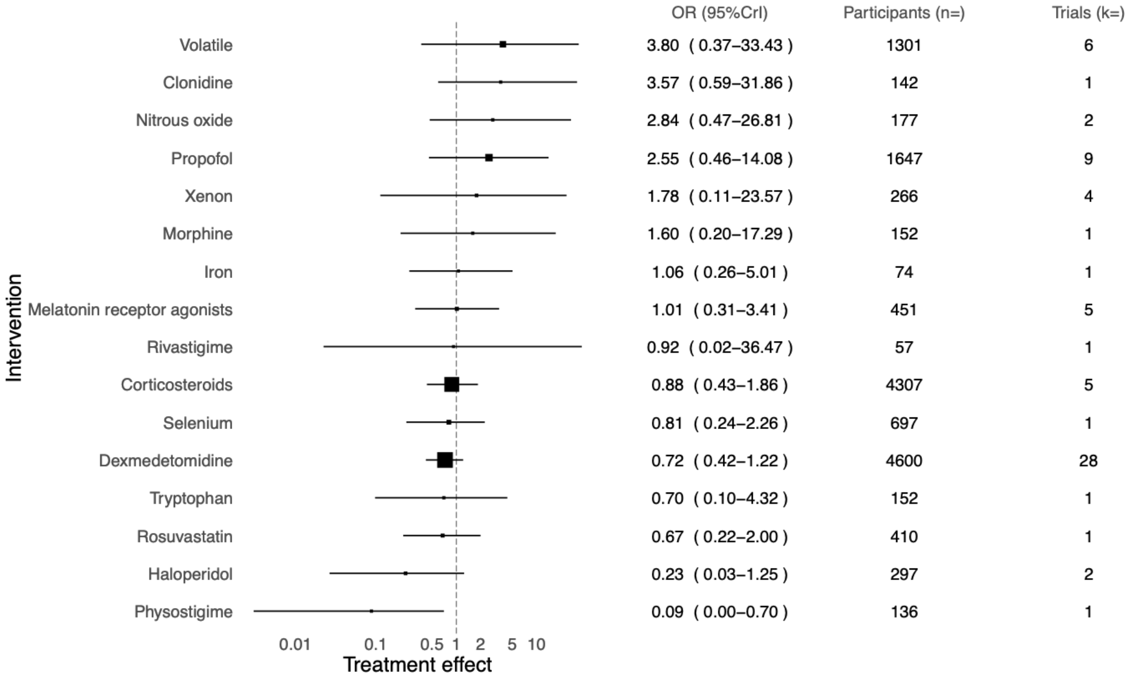

Figure 30 Forest plot of network meta-analysis results for mortality following surgery in delirium prevention trials

Lower values favour intervention. Placebo is the reference value.

Table 23 Effect estimates including all interventions reporting mortality within 1 month of surgery

Intervention	OR (95%CrI)	Trials	Participants
Physostigime	0.09 (0.00-0.70)	1	136
Haloperidol	0.23 (0.03-1.25)	2	297
Rosuvastatin	0.67 (0.22-2.00)	1	410
Tryptophan	0.70 (0.10-4.32)	1	152
Dexmedetomidine	0.72 (0.42-1.22)	28	4,600
Selenium	0.81 (0.24-2.26)	1	697
Corticosteroids	0.88 (0.43-1.86)	5	4,307
Rivastigime	0.92 (0.02-36.47)	1	57
Melatonin receptor agonists	1.01 (0.31-3.41)	5	451
Iron	1.06 (0.26-5.01)	1	74
Morphine	1.60 (0.20-17.29)	1	152
Xenon	1.78 (0.11-23.57)	4	266
Propofol	2.55 (0.46-14.08)	9	1,647
Nitrous oxide	2.84 (0.47-26.81)	2	177

Intervention	OR (95%CrI)	Trials	Participants
Clonidine	3.57 (0.59-31.86)	1	142
Volatile	3.80 (0.37-33.43)	6	1,301

OR, Odds Ratio; 95%CrI, 95% Credible Interval

Delirium severity

There were 23 trials which reported delirium severity. Ten trials used the Cognitive Assessment Method – Severity (CAM-S) score, six trials used the Memorial Delirium Assessment Scale, four trials used the Delirium Rating Scale, two trials used the Intensive Care Delirium Screening Checklist score and one trial used the Delirium Severity Score. Gross and colleagues have published a crosswalk tool for the comparison of trial data using different delirium severity scores.[26] Mohammadi 2016 used the delirium severity scale, van Norden 2021 and Likhvantsev 2021 used the ICDSC neither of which had a crosswalk and were excluded from analysis. One study that used MDAS only reported change in baseline and was excluded (Marcantonio 2011). Consequently, comparison was possible for 19 of the 23 studies which reported delirium severity.

Where studies reported only median severity (with interquartile range or range), methods established by Wan and colleagues were used to estimate mean (standard deviation).[27]

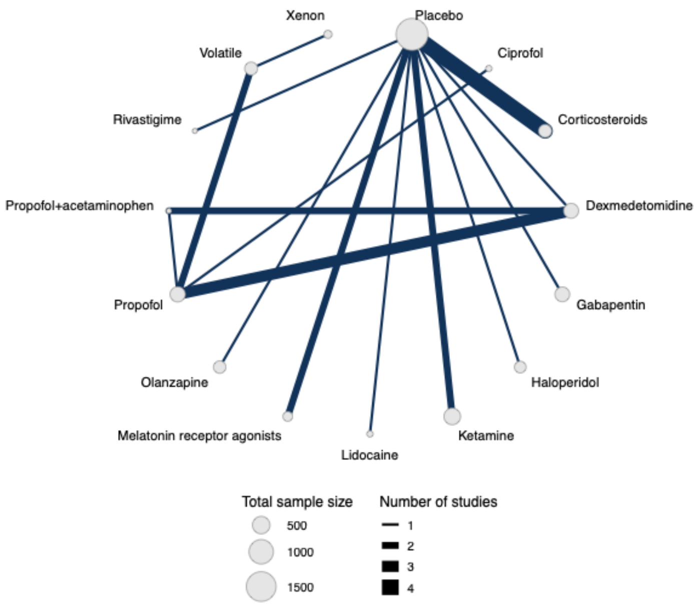

Figure 31 Network graph depicting the connectedness of the network for trials reporting delirium severity

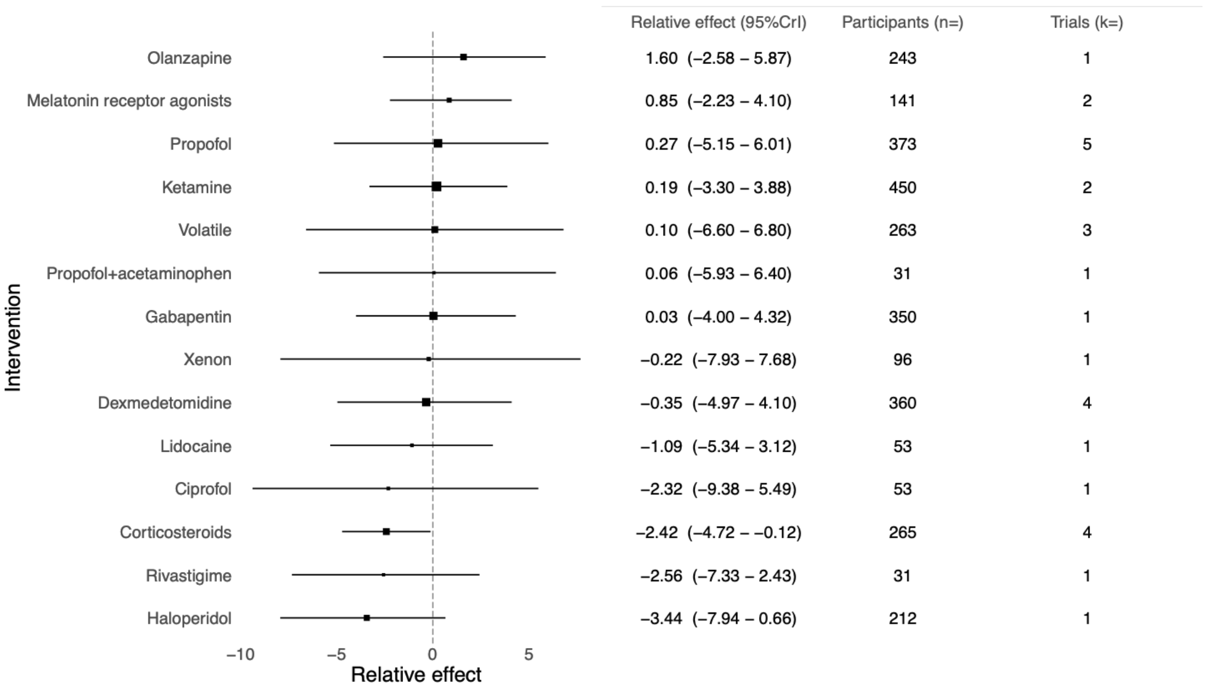

Figure 32 Forest plot of network meta-analysis results for reduction in delirium severity following surgery

Lower values favour intervention. Effect estimates are Memorial Delirium Assessment Scale scores (lower is better). Placebo is the reference value.

Table 24 Effect estimates for delirium severity (all interventions studied)

Intervention	Relative effect (95%CrI)	Trials	Participants
Haloperidol	-3.44 (-7.94 - 0.66)	1	212
Rivastigime	-2.56 (-7.33 - 2.43)	1	31
Corticosteroids	-2.42 (-4.72 - -0.12)	4	265
Ciprofol	-2.32 (-9.38 - 5.49)	1	53
Lidocaine	-1.09 (-5.34 - 3.12)	1	53
Dexmedetomidine	-0.35 (-4.97 - 4.10)	4	360
Xenon	-0.22 (-7.93 - 7.68)	1	96
Gabapentin	0.03 (-4.00 - 4.32)	1	350
Propofol+acetaminophen	0.06 (-5.93 - 6.40)	1	31
Volatile	0.10 (-6.60 - 6.80)	3	263
Ketamine	0.19 (-3.30 - 3.88)	2	450
Propofol	0.27 (-5.15 - 6.01)	5	373
Melatonin receptor agonists	0.85 (-2.23 - 4.10)	2	141

Intervention	Relative effect (95%CrI)	Trials	Participants
Olanzapine	1.60 (-2.58 - 5.87)	1	243

95%CrI, 95% Credible Interval

Length of stay

85 trials comparing 35 interventions including placebo reported the duration of admission in each treatment arm (‘hospital length of stay’). Two interventions (edavarone and penehyclidine) showed a statistically significant reduction in hospital length of stay (LOS); however both were single centre studies and each rated high risk of bias with the Cochrane RoB2 tool (Xie 2021 – edavarone, and Hongyu 2019 – penehyclidine). No other interventions studied reduced hospital length of stay relative to placebo.

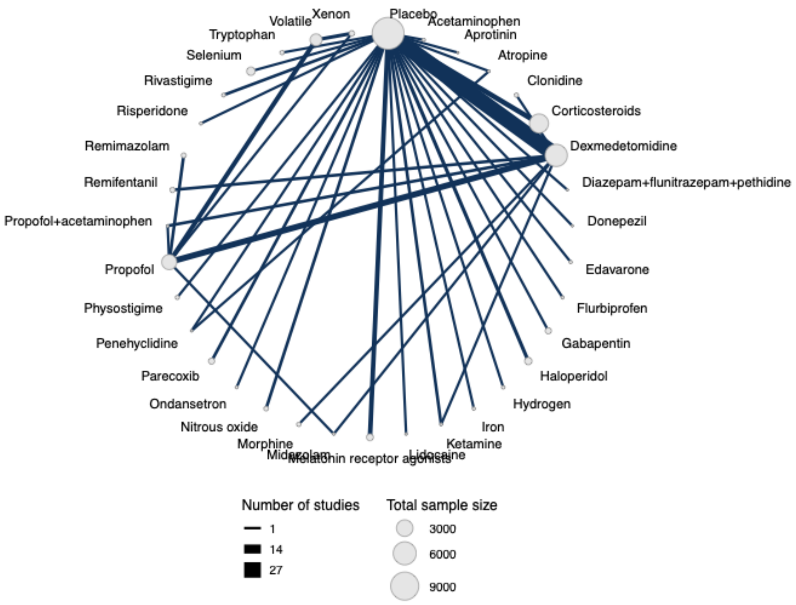

Figure 33 Network graph depicting the connectedness of the network for trials reporting length of stay in delirium prevention trials

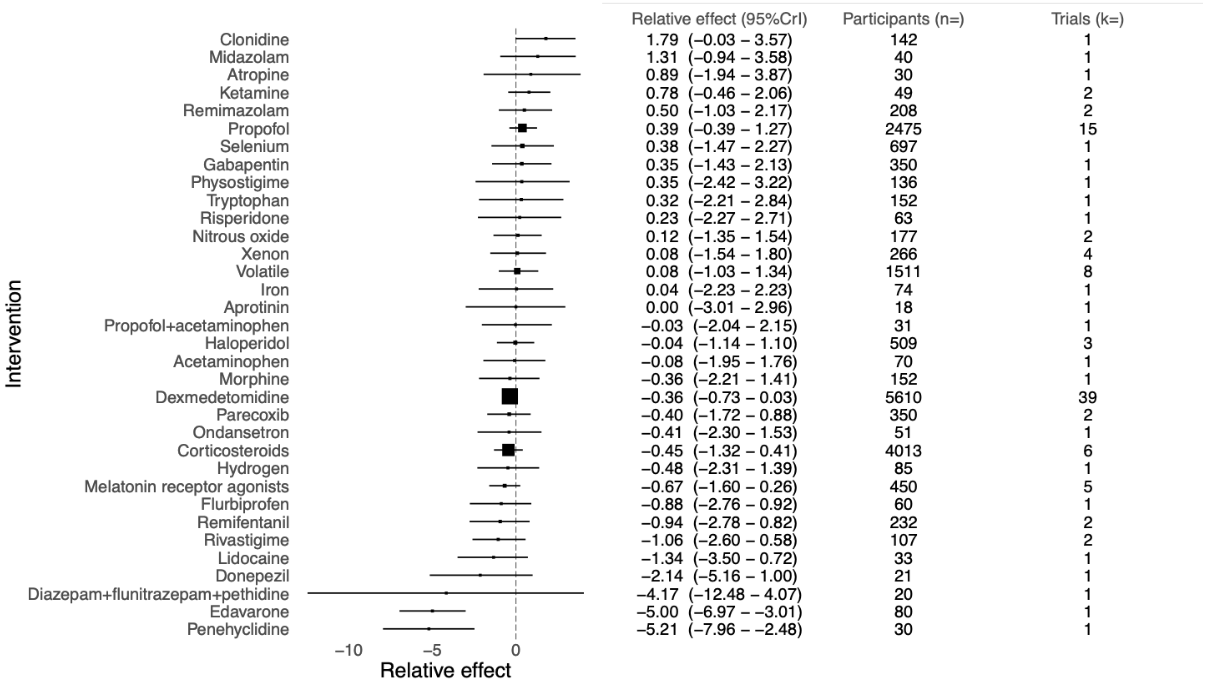

Figure 34 Forest plot of network meta-analysis results for postoperative length of stay in delirium prevention trials

Lower values favour intervention. Effect is duration of admission in days. Placebo is the reference value.

Table 25 Effect estimates for length of stay in delirium prevention trials

Intervention	Relative effect (95%CrI)	Trials	Participants
Penehyclidine	-5.21 (-7.96 - -2.48)	1	30
Edavarone	-5.00 (-6.97 - -3.01)	1	80
Diazepam+flunitrazepam+pethidine	-4.17 (-12.48 - 4.07)	1	20
Donepezil	-2.14 (-5.16 - 1.00)	1	21
Lidocaine	-1.34 (-3.50 - 0.72)	1	33
Rivastigime	-1.06 (-2.60 - 0.58)	2	107
Remifentanil	-0.94 (-2.78 - 0.82)	2	232
Flurbiprofen	-0.88 (-2.76 - 0.92)	1	60
Melatonin receptor agonists	-0.67 (-1.60 - 0.26)	5	450
Hydrogen	-0.48 (-2.31 - 1.39)	1	85
Corticosteroids	-0.45 (-1.32 - 0.41)	6	4,013
Ondansetron	-0.41 (-2.30 - 1.53)	1	51

Intervention	Relative effect (95%CrI)	Trials	Participants
Parecoxib	-0.40 (-1.72 - 0.88)	2	350
Dexmedetomidine	-0.36 (-0.73 - 0.03)	39	5,610
Morphine	-0.36 (-2.21 - 1.41)	1	152
Acetaminophen	-0.08 (-1.95 - 1.76)	1	70
Haloperidol	-0.04 (-1.14 - 1.10)	3	509
Propofol+acetaminophen	-0.03 (-2.04 - 2.15)	1	31
Aprotinin	0.00 (-3.01 - 2.96)	1	18
Iron	0.04 (-2.23 - 2.23)	1	74
Volatile	0.08 (-1.03 - 1.34)	8	1,511
Xenon	0.08 (-1.54 - 1.80)	4	266
Nitrous oxide	0.12 (-1.35 - 1.54)	2	177
Risperidone	0.23 (-2.27 - 2.71)	1	63
Tryptophan	0.32 (-2.21 - 2.84)	1	152
Physostigime	0.35 (-2.42 - 3.22)	1	136
Gabapentin	0.35 (-1.43 - 2.13)	1	350
Selenium	0.38 (-1.47 - 2.27)	1	697
Propofol	0.39 (-0.39 - 1.27)	15	2,475
Remimazolam	0.50 (-1.03 - 2.17)	2	208
Ketamine	0.78 (-0.46 - 2.06)	2	49
Atropine	0.89 (-1.94 - 3.87)	1	30
Midazolam	1.31 (-0.94 - 3.58)	1	40
Clonidine	1.79 (-0.03 - 3.57)	1	142

95%CrI, 95% Credible Interval

Quality of life

8 trials reported quality of life (QoL) after discharge, they used 6 different tools or variants of a tool, measuring the QoL between 30 days to 1 year after surgery, comprising 2720 participants. The majority of the trials (n=6/8) compared dexmedetomidine to saline. All were two arm studies; none had an active comparison arm. The data are summarized in Table 26.

Table 26 Summary of quality of life measure results in delirium prevention trials

**Study**	**Intervention**	**n=**	**QoL Intervention**	**QoL Control**	**QoL measure**	**Timepoint**	**Statistical measure**
Turan 2020	dexmedetomidine	794	51 (43–55)<br>57 (49–60)	50 (43–55)<br>57 (51–60)	SF-12 physical<br>SF-12 mental	90 days	median (IQR)
Hong 2021	dexmedetomidine	712	55.4 (14.3)<br>73.1 (13.4)<br>72.5 (5.7)<br>63.1 (10.4)	51.6 (14.6)<br>70.3 (11.1)<br>71.9 (6.2)<br>61.7 (10.8)	WHO QOL physiology<br>WHO QOL psychology<br>WHO QOL social<br>WHO QOL environment	30 days	mean (SD)
Haller 2021	nitrous oxide	140	56.7(10.4)<br>73.4(16.8)	58.4(10.3)<br>72.4(19.7)	EQ-5D utility<br>EQ-5D VAS	90 days	mean (SD)
Xie 2021	edavarone	160	74.3(12.6)<br>79.6(11.7)	61.2(13.1)<br>65.6(16.6)	Barthel	1 month<br>12 months	mean (SD)
Chitnis 2022	dexmedetomidine	70	67.5	68.71	SF-36	6 months	mean*
Qu 2023	dexmedetomidine	394	54.1 [47.7, 59.8]<br>59.0 [53.3, 67.6]<br>48.8 [43.4, 56.1]	54.1 [47.7, 57.7]<br>62.5 [56.0, 67.6]<br>48.2 [42.2, 52.5]	PROMIS physical<br>PROMIS mental<br>PROMIS function	90 days	median (IQR)
Zhang 2023	dexmedetomidine	117	62 (11)<br>70 (9)<br>73 (9)<br>66 (9)	62 (11)<br>70 (11)<br>70 (10)<br>63 (9)	WHO QOL physiology<br>WHO QOL psychology<br>WHO QOL social<br>WHO QOL environment	30 days	mean (SD)
Huet 2024	dexmedetomidine	333	45 (8.9)<br>51 (9.5)	43.3 (8.1)<br>49.3 (10.1)	SF-36 physical<br>SF-36 mental	3 months	mean (SD)

n=, number of participants; SF-12, 12 item Short Form Survey; WHO QOL, WHO Quality of Life Brief (26 item); SF-36, 36-item Short Form Survey; Barthel, Barthel Index; PROMIS, Patient-Reported Outcomes Measurement Information System®; *variance not reported; IQR, interquartile range; SD, standard deviation

Postoperative neurocognitive dysfunction (≥3 months)

15 trials assessed for postoperative cognitive dysfunction (POCD) at a time point later than discharge from hospital, of which ten reported on POCD at least three months after surgery. None of these ten trials used the same measure of POCD at the same time point precluding robust meta-analysis of the effects of the studied treatments on POCD at 3 or more months after surgery. The results of all trials assessing POCD after discharge are summarized in Table 27. Among the larger group of 15 trials that assessed POCD at various timepoints between 1 month and 12 months after surgery the most common instrument used was the Telephone Interview for Cognitive Status (or variant thereof) which was used in eight trials.

Table 27 Secondary outcome: Postoperative cognitive dysfunction measured at timepoints later than discharge from hospital

Study	Intervention	Control	n	POCD Intervention	POCD Control	POCD measure	Timepoint	Statistical Measure
Li 2017	dexmedetomidine	placebo	285	34 (32 to 37)	34 (32 to 36)	TICS-M	1 month	median (IQR)
Xie 2021	edavarone	placebo	160	39.63(4.35)<br>40.13(5.93)	33.63(5.81)<br>34.13(5.36)	TICS-M	1 month<br>12 months	mean (SD)
Hong 2021	dexmedetomidine	placebo	712	28.4 (5)	28.8 (5)	TICS-M	1 month	mean (SD)
Cao 2023	propofol	sevoflurane	1228	28.2 (4.5)	28.5 (4.6)	TICS-M	1 month	mean (SD)
Zhang 2023	dexmedetomidine	placebo	117	29 (4)	29 (3)	TICS-M	1 month	mean (SD)
Long 2024	dexmedetomidine	placebo	78	9 (7–10)	8.5 (7–10)	TICS-10	1 month	median (IQR)
Royse 2011	propofol	desflurane	182	56⁄84 (67.5)	41⁄83 (49.4)	Multiple*	3 months	n/N (%)
Ford 2020	melatonin	placebo	205	23/84 (27.4)	23/82 (28.0)	TICS-M	3 months	n/N (%)
Spies 2021	physostigime	placebo	277	10/75 (13)	4/60 (6.7)	ISPOCD†	3 months	n/N (%)
Gao 2021	dexmedetomidine	placebo	40	28.4 ± 1.3	26.6 ± 1.5	MMSE	3 months	mean (SD)
Takazawa 2023	minocycline	placebo	202	11/82<br>27.3(2.6)	3/83<br>27.1(1.2)	ISPOCD†<br>MMSE	3 months	mean (SD)
Qu 2023	dexmedetomidine	placebo	394	20.0 [19.0, 21.0]	20.0 [19.0, 21.0]	abbreviated MOCA	3 months	median (IQR)
Huet 2024	dexmedetomidine	placebo	333	23.7 (13.6)	25.4 (12.9)	CFQ	3 months	mean (SD)
Al Tmimi 2020	xenon	sevoflurane	190	31 (4.2)	32 (3.9)	TICS	6 months	mean (SD)
Chitnis 2022	dexmedetomidine	placebo	70	54.58	51.74	MCAS	6 months	mean

TICS-M, modified Telephone Interview for Cognitive Status; TICS-10, 10 item Telephone Interview for Cognitive Status; Multiple*, >1SD in two or more tests from 12 test battery (Trailmaking A and B, Controlled Word Association test, Stroop, Letter cancellation, Grooved Pegboard Dominant and Non- Dominant Hands, Rey Auditory Learning test, Digit Span, Symbol Digit Modalities); ISPOCD†, International Study of Post-Operative Cognitive Dysfunction criteria as defined in Moller et al[28]; MMSE, Mini-Mental State Exam; MOCA, Montreal Objective Cognitive Assessment; CFQ, Cognitive Failure Questionnaire; MCAS, Minnesota Cognitive Acuity Screen; n, number positive for POCD; N, sample population; IQR, interquartile range; SD, standard deviation

Postoperative complications

The granularity of reporting varied considerably, with studies often reporting only drug related adverse events, drug related adverse events, unrelated adverse events, or operation related complications. Rarely all of these complications. Rarely all of these were reported. Eight trials specifically reported no complications across all complications across all arms, although these trials only reported on one to three complications. Supplementary complications. Supplementary file 4 contains all data available for interventions where complications were where complications were reported. For the interventions where efficacy in the primary outcome was found primary outcome was found (dexmedetomidine, corticosteroids, melatonin receptor agonists, parecoxib, agonists, parecoxib, olanzapine and insulin) we qualitatively synthesized the complication rates reported for complication rates reported for these interventions.

Figure 36 to

Figure 64 report these data.

Table 28 Secondary outcome: Postoperative complications by number of trials reporting each complication

Complication	Number of trials
Hypotension	53
Bradycardia	45
Thromboembolic Event	42
Postoperative nausea and vomiting	35
Surgical site infection	25
Pneumonia	23
Stroke	23
Atrial fibrillation	21
Acute kidney injury	21
Tachycardia	20
Hypoxia	17
Arrhythmia	16
Infection	16
Myocardial Infarction	12
Renal Failure	12
Serious Adverse Event	11
Sepsis	10
Dizziness	9
Respiratory Failure	9

Complication	Number of trials
Urinary tract infection	9
Ventricular Arrhythmia	8
Composite count	7
Heart Failure	7
Adverse Events Nos	6
Extrapyramidal side effects	5
Sedation	5
Acute coronary syndrome	4
Abnormal Liver Enzymes	3
Adverse event (Drug Related)	3
Diarrhoea	3
Hallucinations	3
Heart Block	3
Supraventricular tachycardia	3
Cardiac (non specified)	2
Clavien Dindo >=2	2
Hypoglycaemia	2
Postoperative pulmonary complications	2
Prolonged QT interval	2
Respiratory	2
Seizure	2
Serious unexpected severe adverse reaction	2
Abnormal Blood Count	1
Blurred Vision	1
Clavien >=3	1
Dry Mouth	1
Electrolyte Imbalance	1
Gastrointestinal Reaction	1
Insomnia	1

Complication	Number of trials
Local anaesthetic systemic toxicity	1
Major Complications (not otherwise specified)	1
Mild Adverse Events (not otherwise specified)	1
Moderate Adverse Events (not otherwise specified)	1
Neurologic (not otherwise specified)	1
Paraesthesia	1
Postoperative pulmonary complications (Melbourne Scale >=4)	1
Pyrexia	1
Renal (not otherwise specified)	1
Rhabdomyolysis	1
Shivering	1
Skin Flushing	1
Stroke/Transient ischaemic attack	1
Surgical Complications (not otherwise specified)	1
Urinary Retention	1

Postoperative complications reported in trials of dexmedetomidine, corticosteroids, melatonin receptor agonists, parecoxib, olanzapine or insulin

Lower treatment effect values favour intervention. Placebo is the reference value.

Adverse events NOS / Unspecified adverse event(s)

Included complications reported in studies as ‘adverse events’, mild adverse events, moderate adverse events, major complications, Clavien Dindo ≥2, Clavien ≥3, Clavien Dindo ≥3, and composites of multisystem complications (e.g. respiratory and renal and cardiac without further classification)

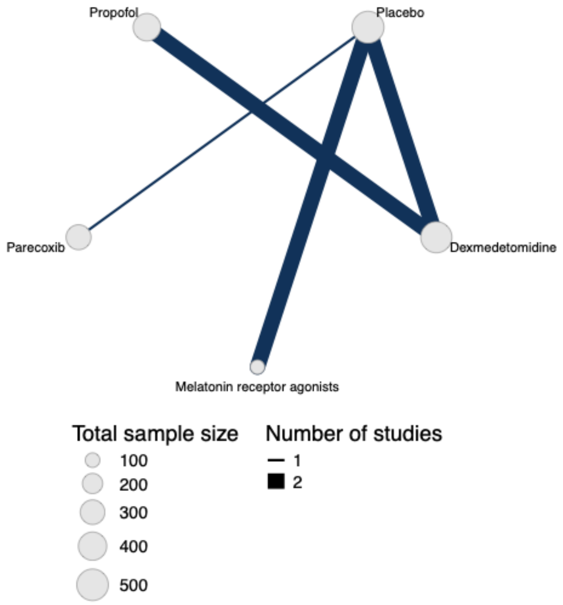

Figure 35 Network graph for the secondary outcome: Complications (Adverse events, not otherwise specified)

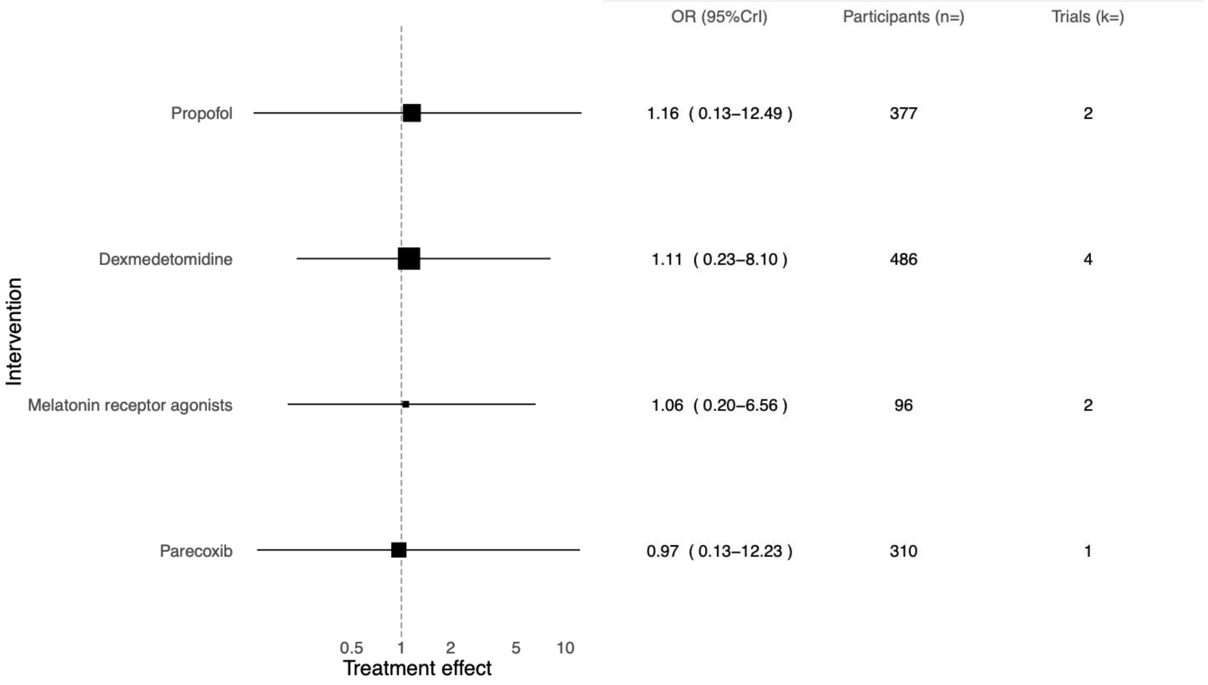

Figure 36 Forest plot of network meta-analysis results for secondary outcome: Complications (adverse events not otherwise specified)

Arrhythmias

Including atrial fibrillation, other supraventricular tachycardias, ventricular arrhythmias (bigeminy, ventricular tachycardia, ventricular fibrillation), heart block, and prolonged QT interval. Sinus bradycardia and sinus tachycardia are reported separately. There were zero events in the control arm of Mu 2017 preventing stable estimates for Parecoxib.

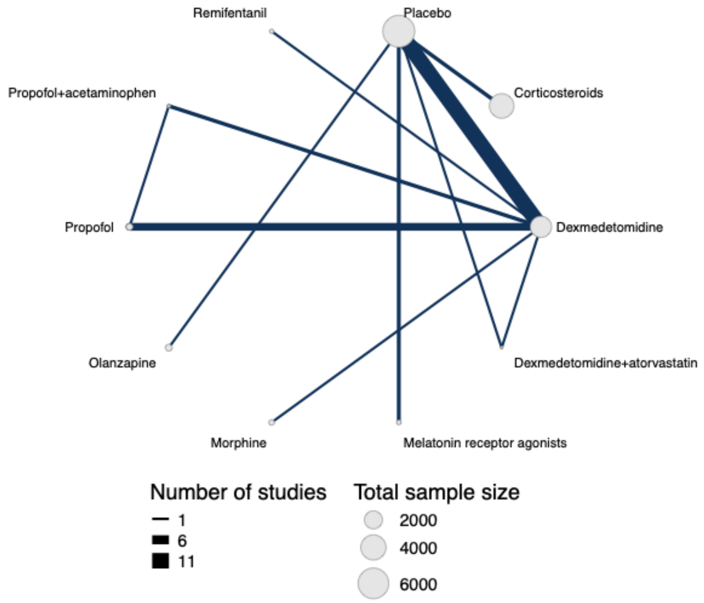

Figure 37 Network graph for the secondary outcome: Complications (arrhythmia)

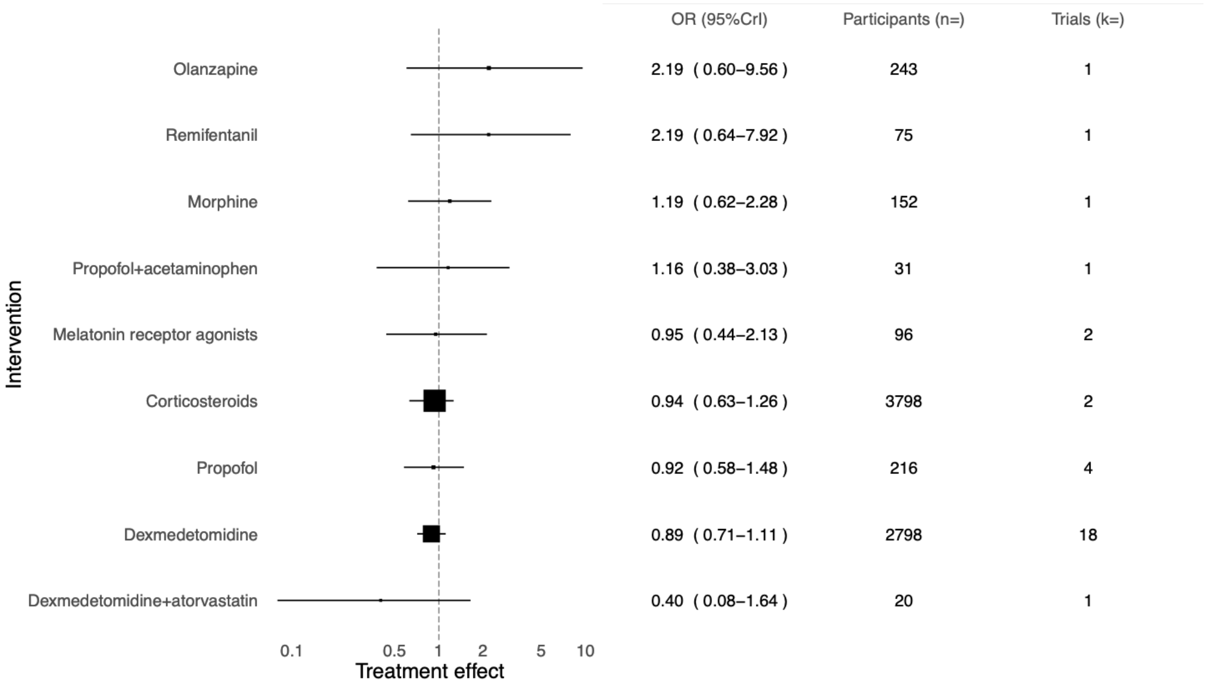

Figure 38 Forest plot of network meta-analysis results for secondary outcome: Complications (arrhythmia)

Bradycardia

Often unspecified, but where specified refers to heart rates between <45 bpm and <55 bpm. Oh 2021 (melatonin receptor agonist) excluded from this analysis – no events in control arm preventing stable estimates of effect.

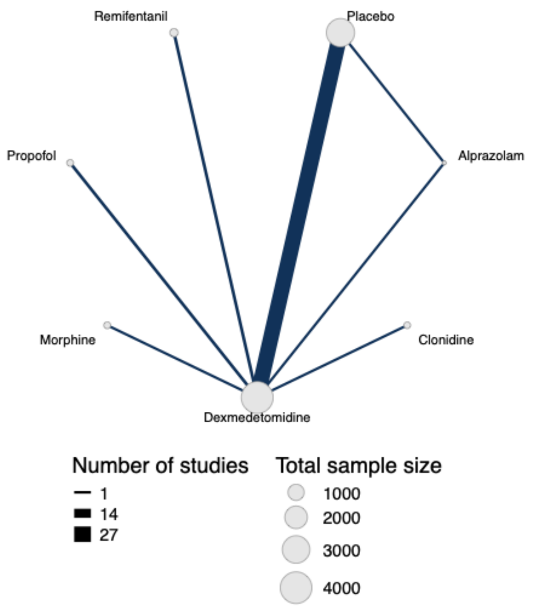

Figure 39 Network graph for the secondary outcome: Complications (bradycardia)

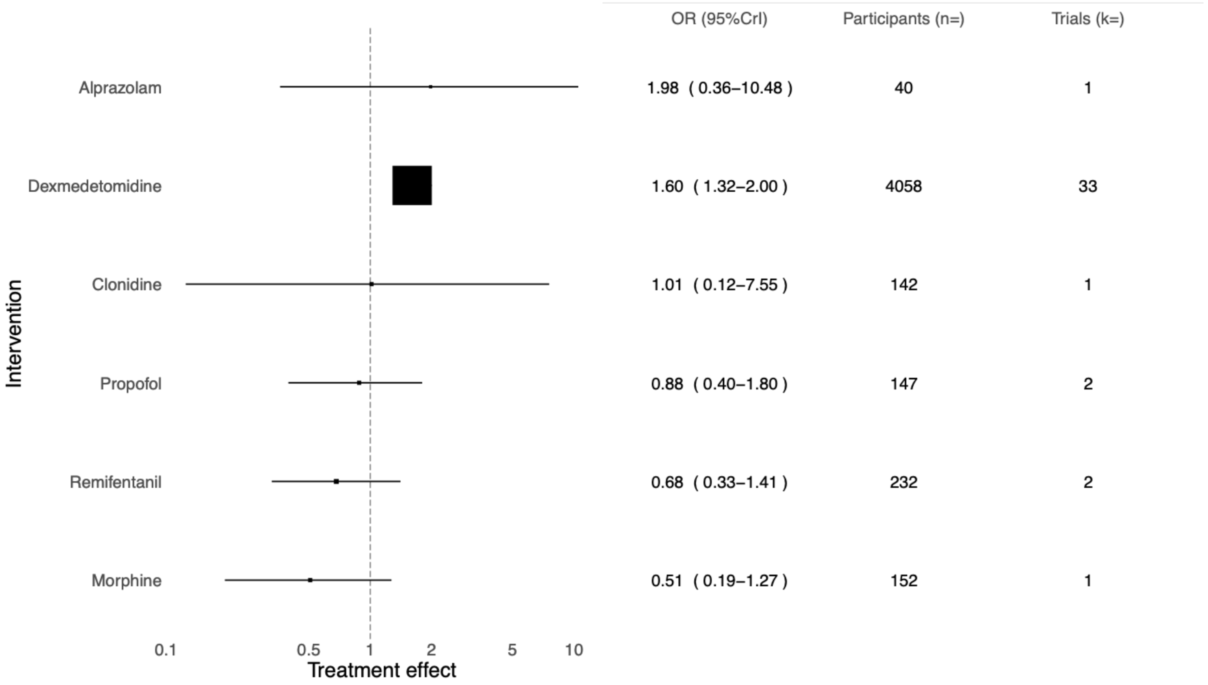

Figure 40 Forest plot of network meta-analysis results for secondary outcome: Complications (arrhythmias)

Cardiac complications

Including acute coronary syndrome, myocardial ischaemia, myocardial infarction, heart failure

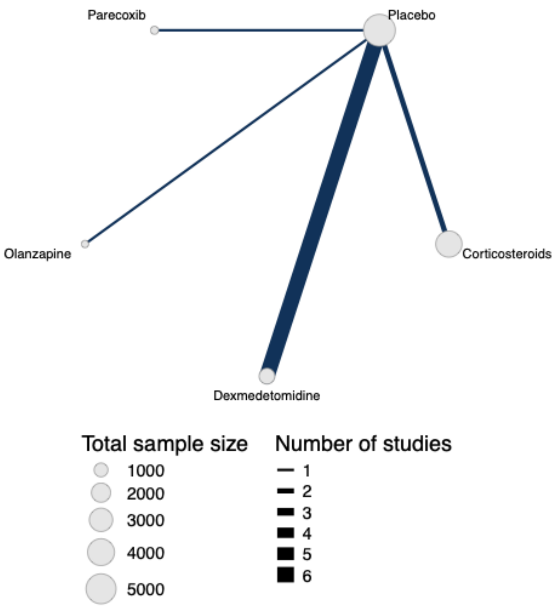

Figure 41 Network graph for the secondary outcome: Complications (Cardiac)

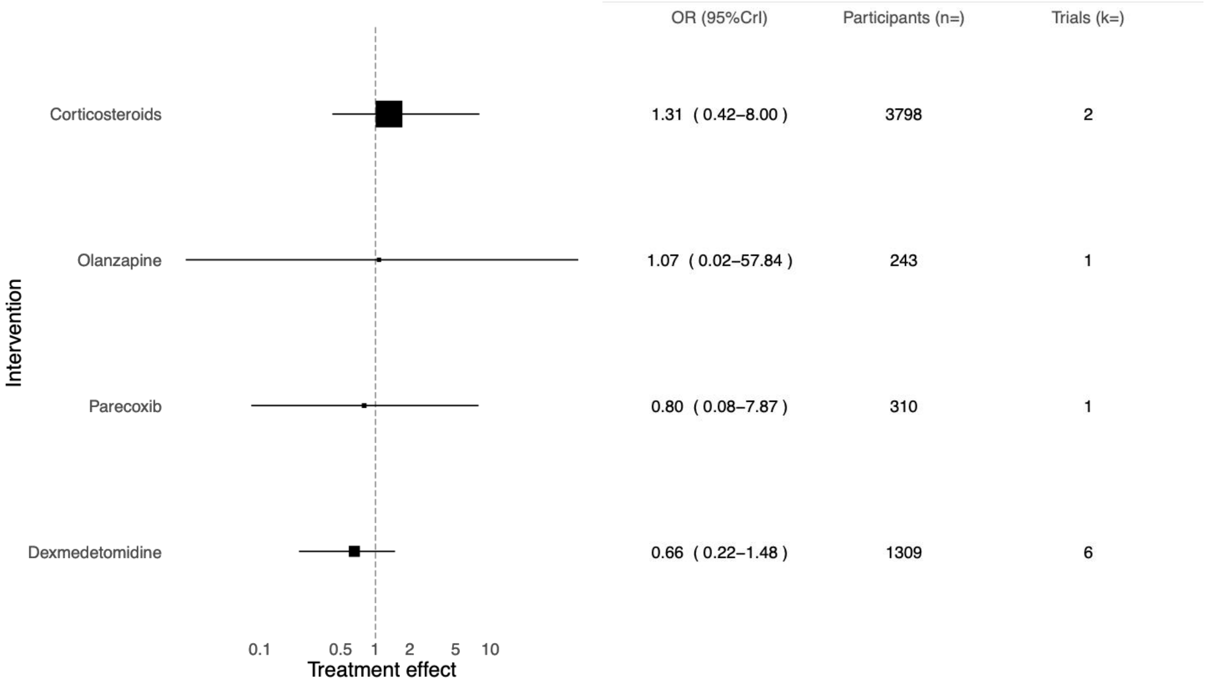

Figure 42 Forest plot of network meta-analysis results for secondary outcome: Complications (arrhythmias)

Hypotension

Often unspecified, but where specified refers to systolic blood pressure <90mmHg. Hypotension more common with dexmedetomidine. Lu 2023 (ketamine) excluded from this analysis – no events in intervention arm preventing stable estimates of effect.

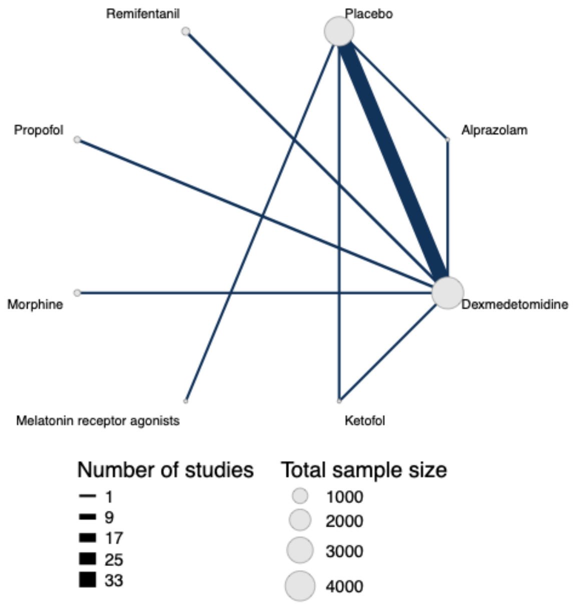

Figure 43 Network graph for the secondary outcome: Complications (hypotension)

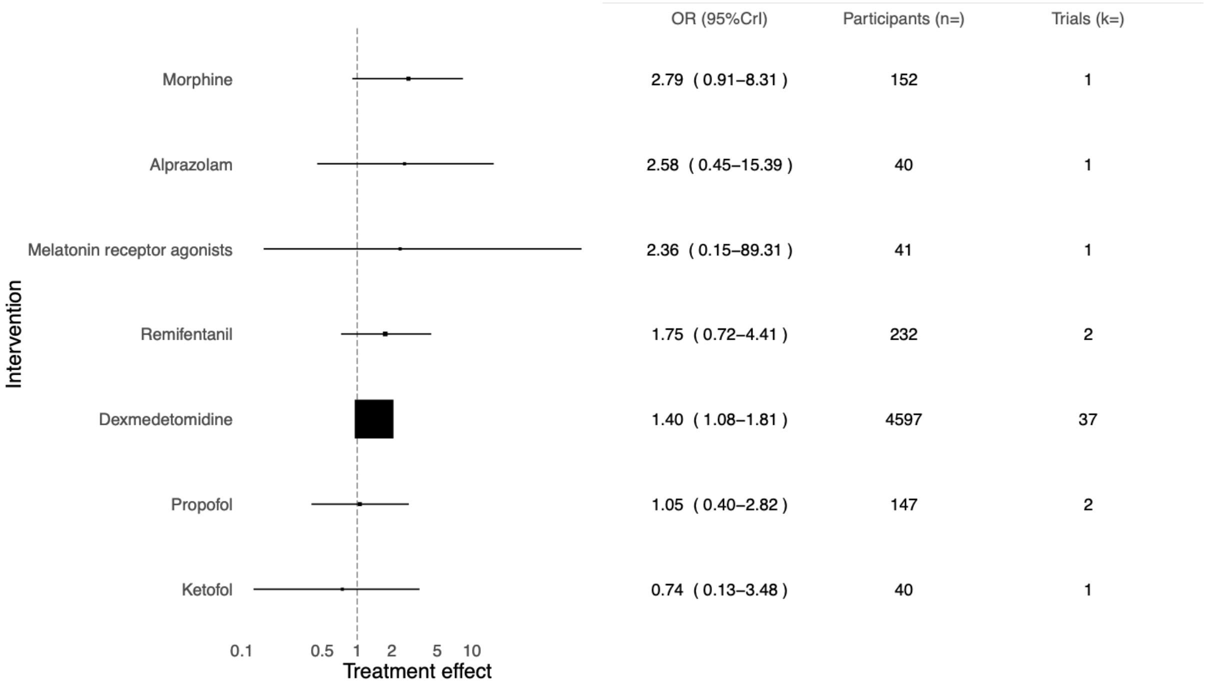

Figure 44 Forest plot of network meta-analysis results for secondary outcome: Complications (arrhythmias)

Hypoxia

Often unspecified, but where specified refers to peripheral oxygen saturation <92%.

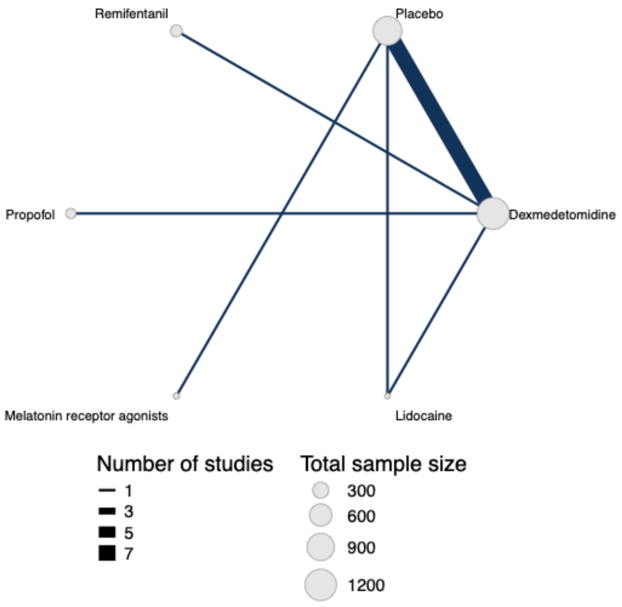

Figure 45 Network graph for the secondary outcome: Complications (hypoxia)

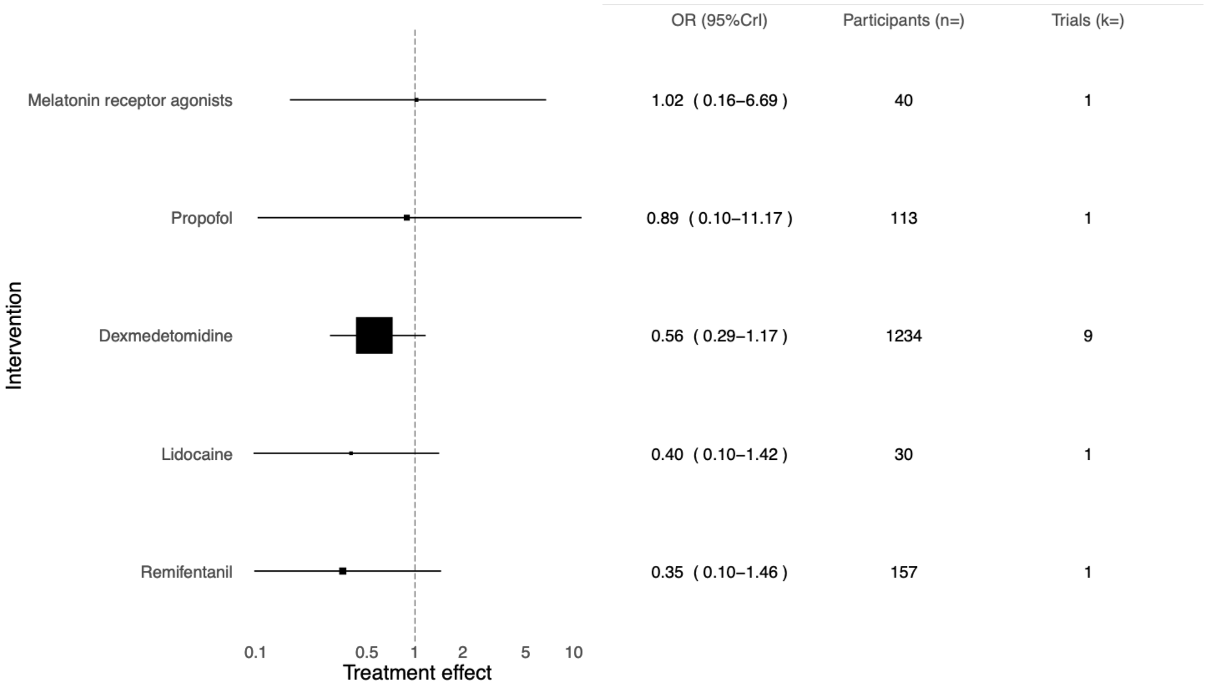

Figure 46 Forest plot of network meta-analysis results for secondary outcome: Complications (arrhythmias)

Infection

Including surgical site or wound infection, pneumonia, urinary tract infection, sepsis, pyrexia or infection with location unspecified.

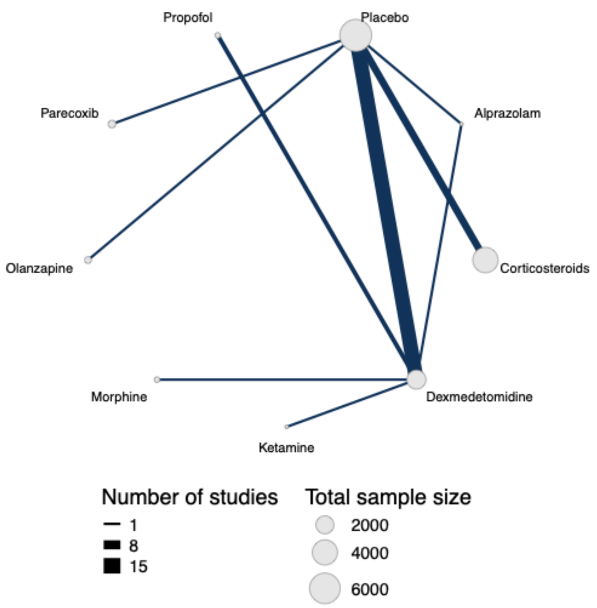

Figure 47 Network graph for the secondary outcome: Complications (infection)

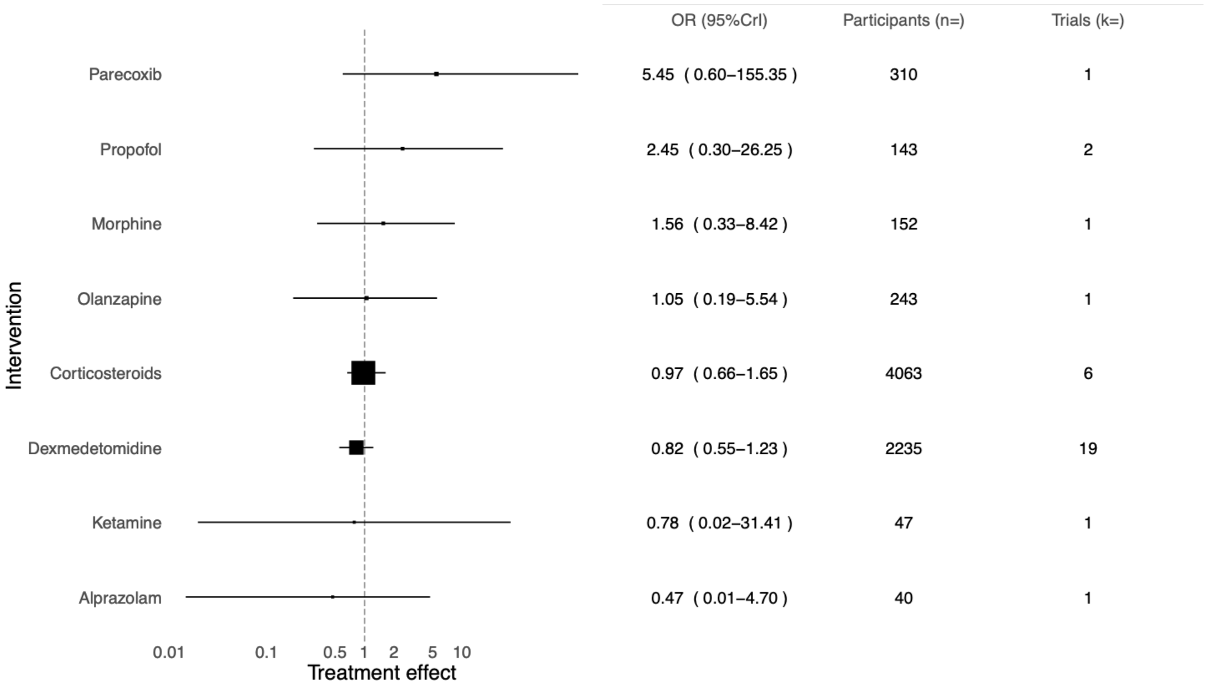

Figure 48 Forest plot of network meta-analysis results for secondary outcome: Complications (arrhythmias)

Neurological

Trials reporting on strokes, transient ischaemic attacks or otherwise unspecified neurologic complications. Ford 2020 (melatonin receptor agonist) and Mu 2017 (parecoxib) excluded due to zero events for analysis.

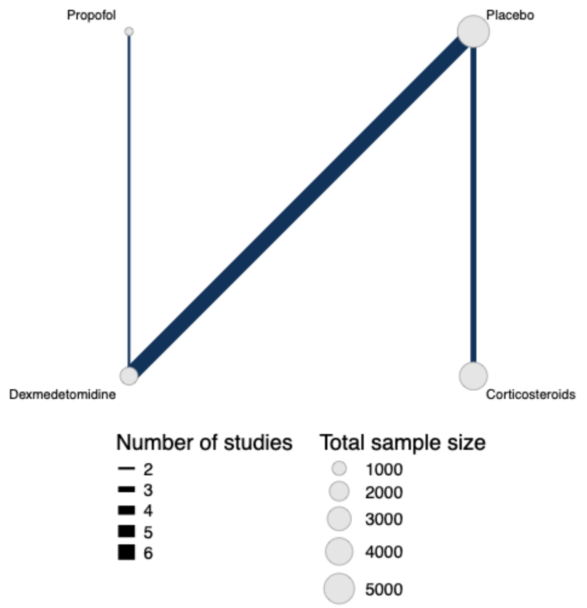

Figure 49 Network graph for the secondary outcome: Complications (neurological)

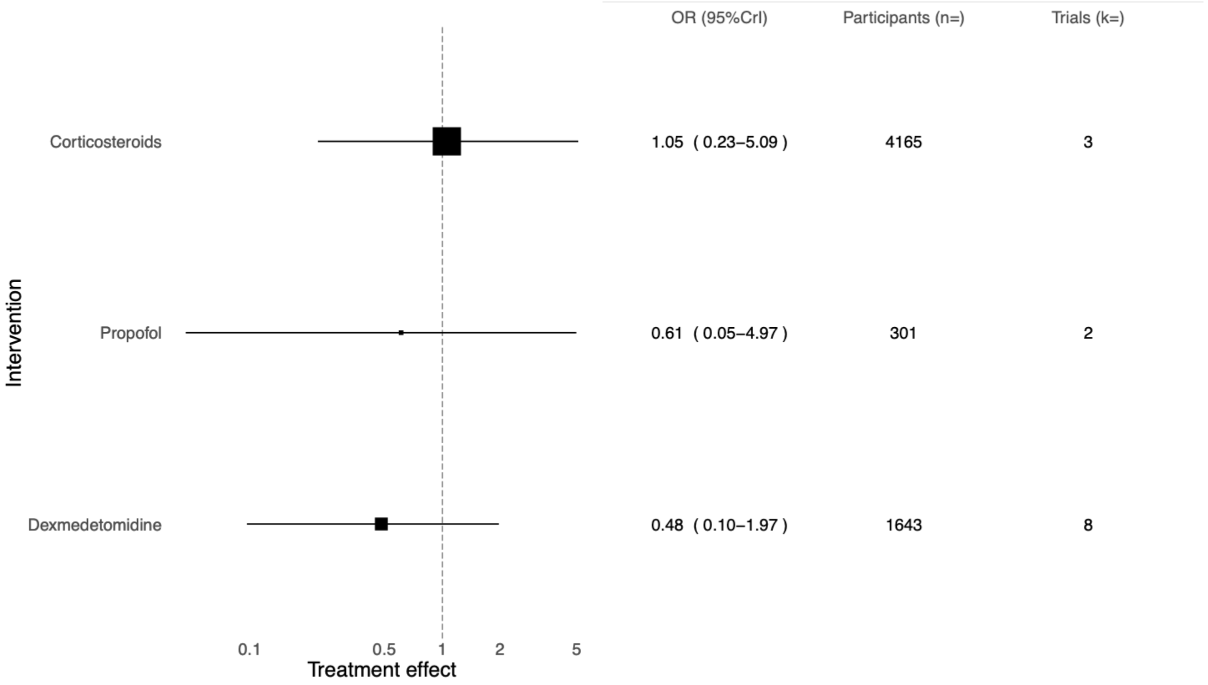

Figure 50 Forest plot of network meta-analysis results for secondary outcome: Complications (arrhythmias)

Other complications

Includes complications not elsewhere grouped: abnormal blood counts, abnormal liver enzymes, insomnia, non-delirium complications, blurred vision, diarrhoea, paraesthesia, skin flushing, hallucinations, gastrointestinal reaction, thromboembolic events, electrolyte imbalances, shivering, dizziness, dry mouth, rhabdomyolysis, urinary retention, surgical complications not otherwise specified

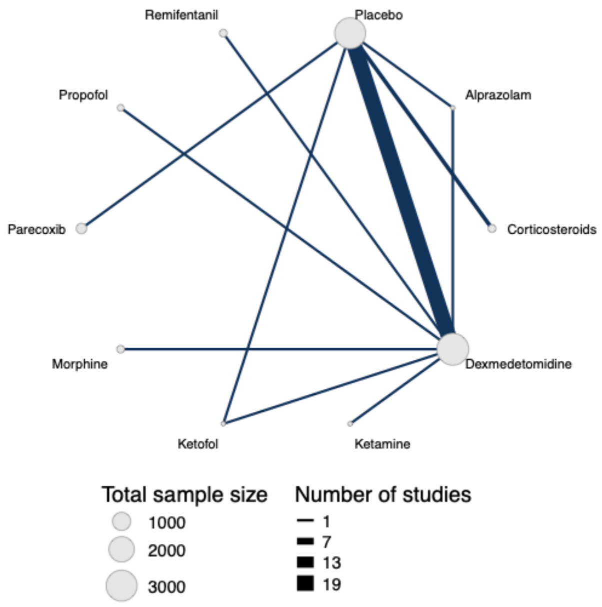

Figure 51 Network graph for the secondary outcome: Complications (other)

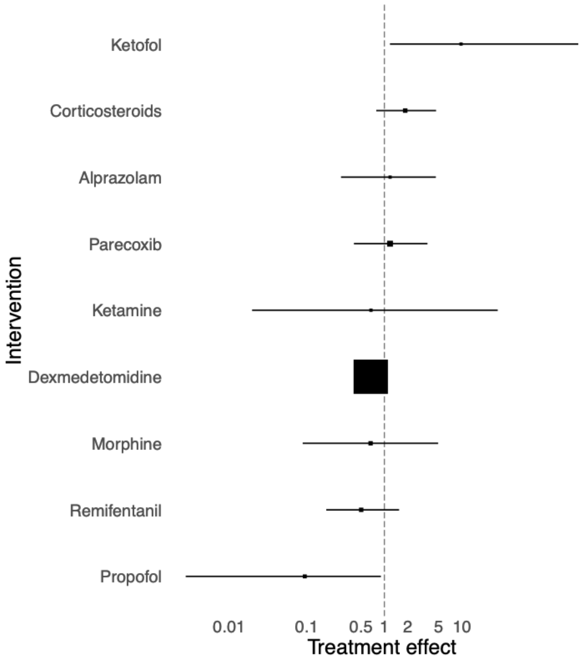

Intervention	OR (95%CrI)	Participants (n=)	Trials (k=)
Ketofol	9.72 (1.18–315.86)	40	1
Corticosteroids	1.85 (0.78–4.64)	163	3
Alprazolam	1.18 (0.28–4.60)	40	1
Parecoxib	1.18 (0.40–3.59)	310	1
Ketamine	0.67 (0.02–28.91)	47	1
Dexmedetomidine	0.67 (0.49–0.90)	3181	23
Morphine	0.66 (0.09–4.93)	152	1
Remifentanil	0.50 (0.18–1.55)	157	1
Propofol	0.09 (0.00–0.90)	113	1

Figure 52 Forest plot of network meta-analysis results for secondary outcome: Complications (arrhythmias)

Postoperative nausea and vomiting (PONV)

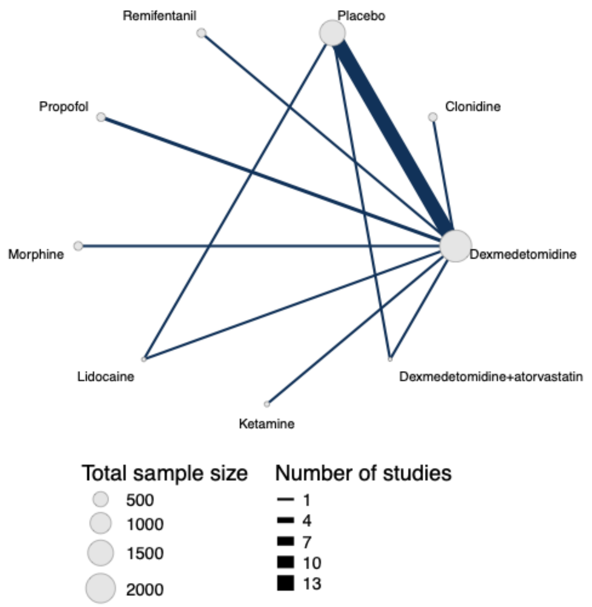

Figure 53 Network graph for the secondary outcome: Complications (postoperative nausea and vomiting)

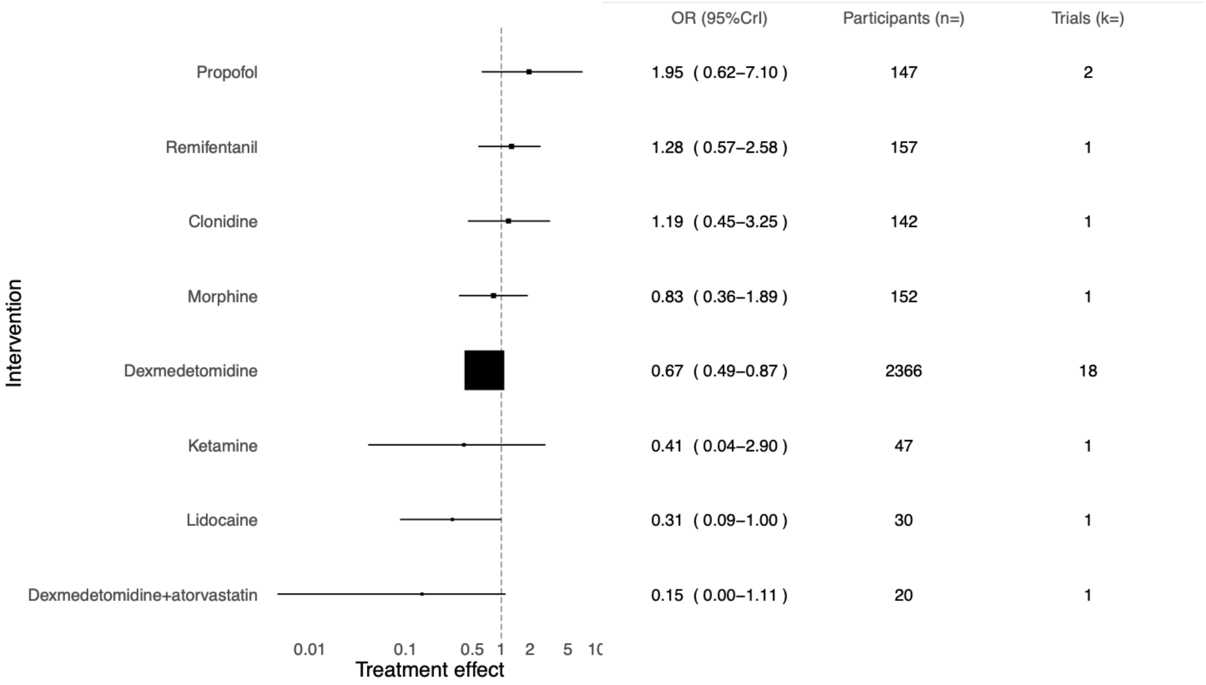

Figure 54 Forest plot of network meta-analysis results for secondary outcome: Complications (arrhythmias)

Renal

Including acute kidney injury (AKI), renal failure, renal injury not otherwise specified

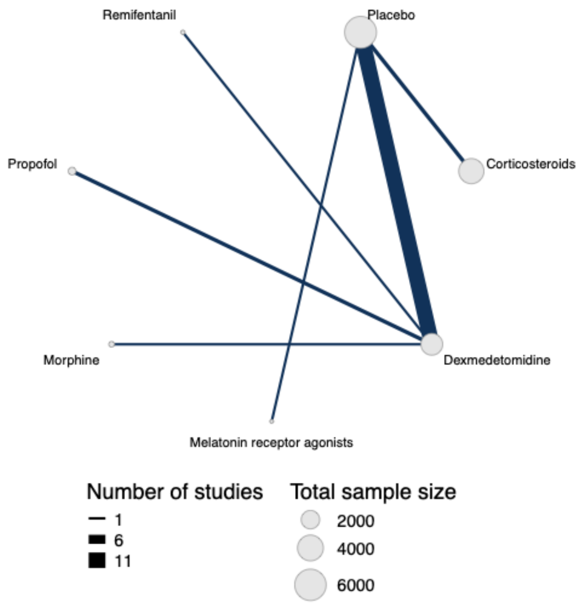

Figure 55 Network graph for the secondary outcome: Complications (renal)

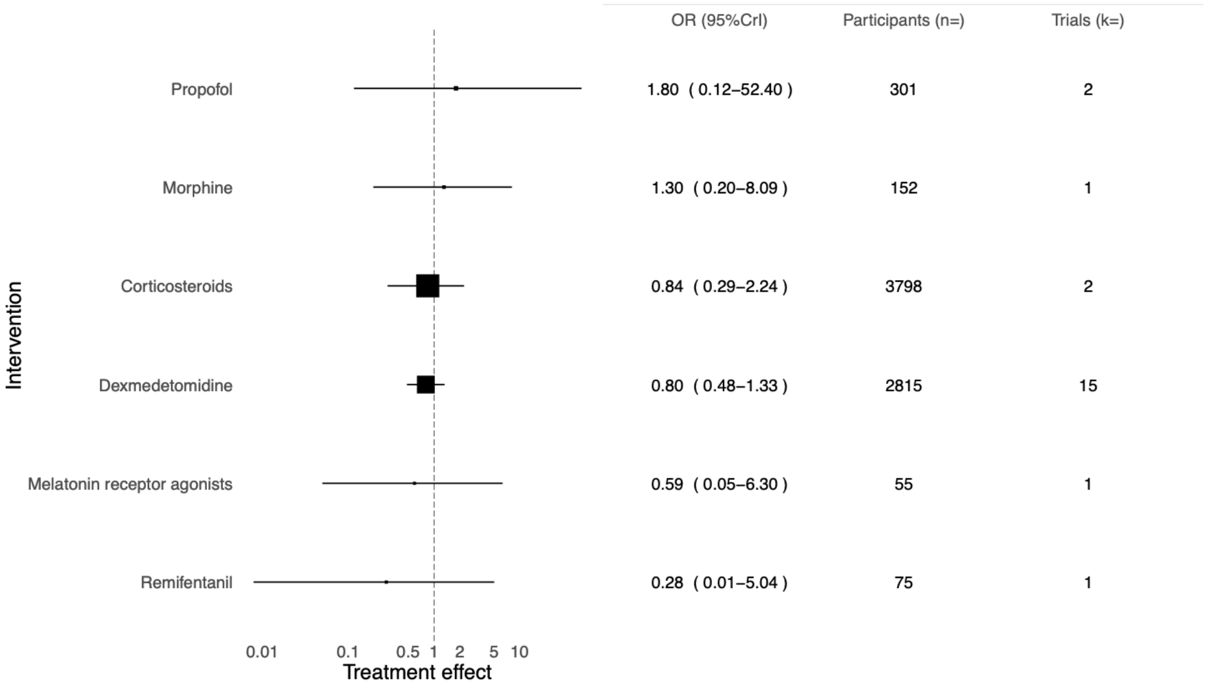

Figure 56 Forest plot of network meta-analysis results for secondary outcome: Complications (arrhythmias)

Respiratory

Respiratory depression, respiratory failure, postoperative pulmonary complications (sometimes defined as Melbourne score ≥4), and respiratory complications not otherwise specified

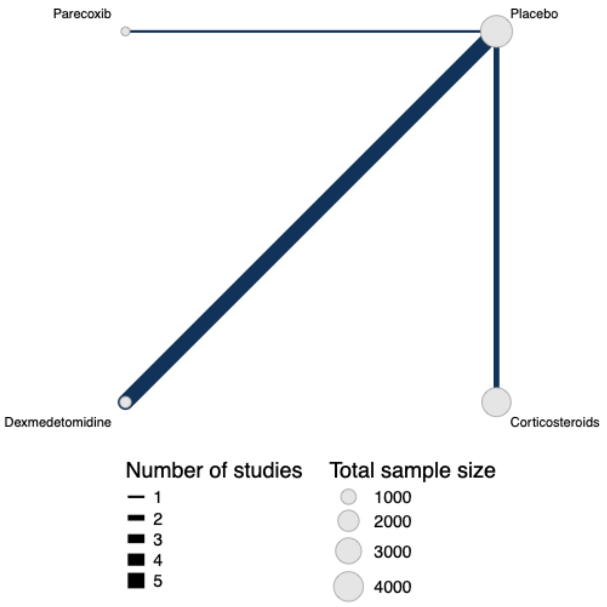

Figure 57 Network graph for the secondary outcome: Complications (respiratory)

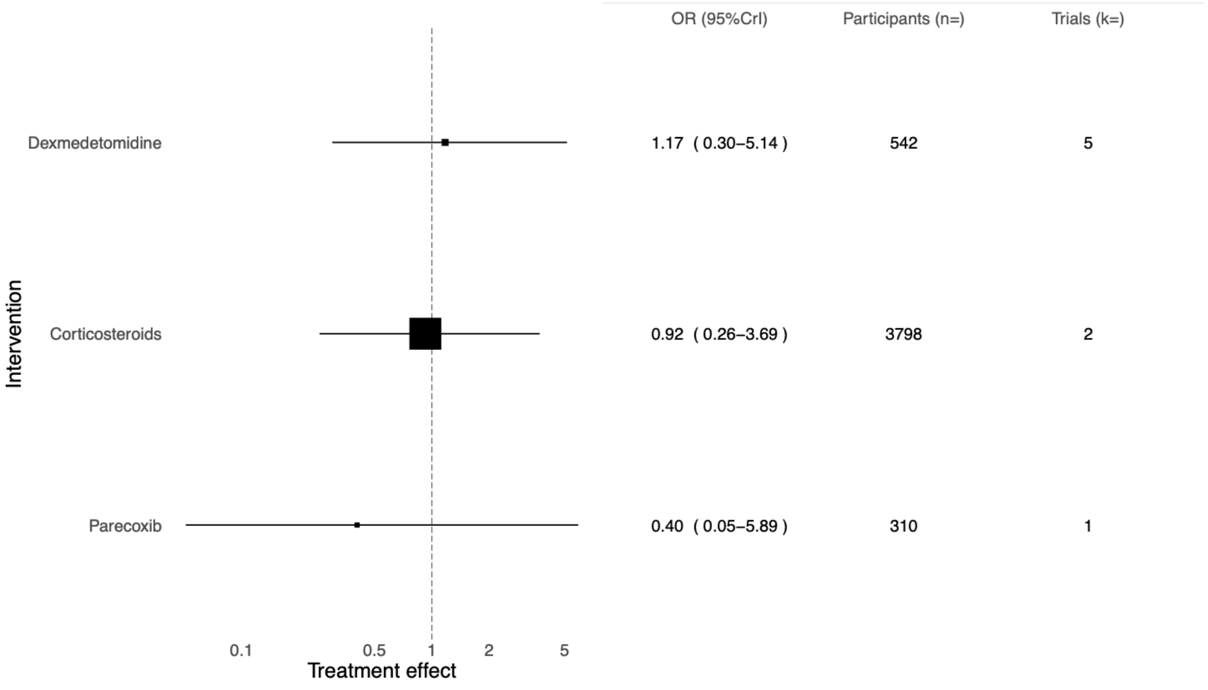

Figure 58 Forest plot of network meta-analysis results for secondary outcome: Complications (arrhythmias)

Serious adverse event(s)

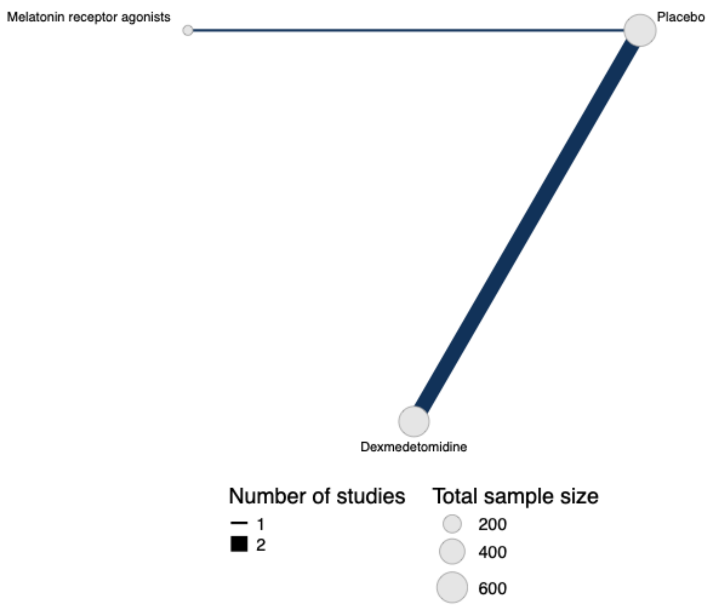

Figure 59 Network graph for the secondary outcome: Complications (serious adverse events)

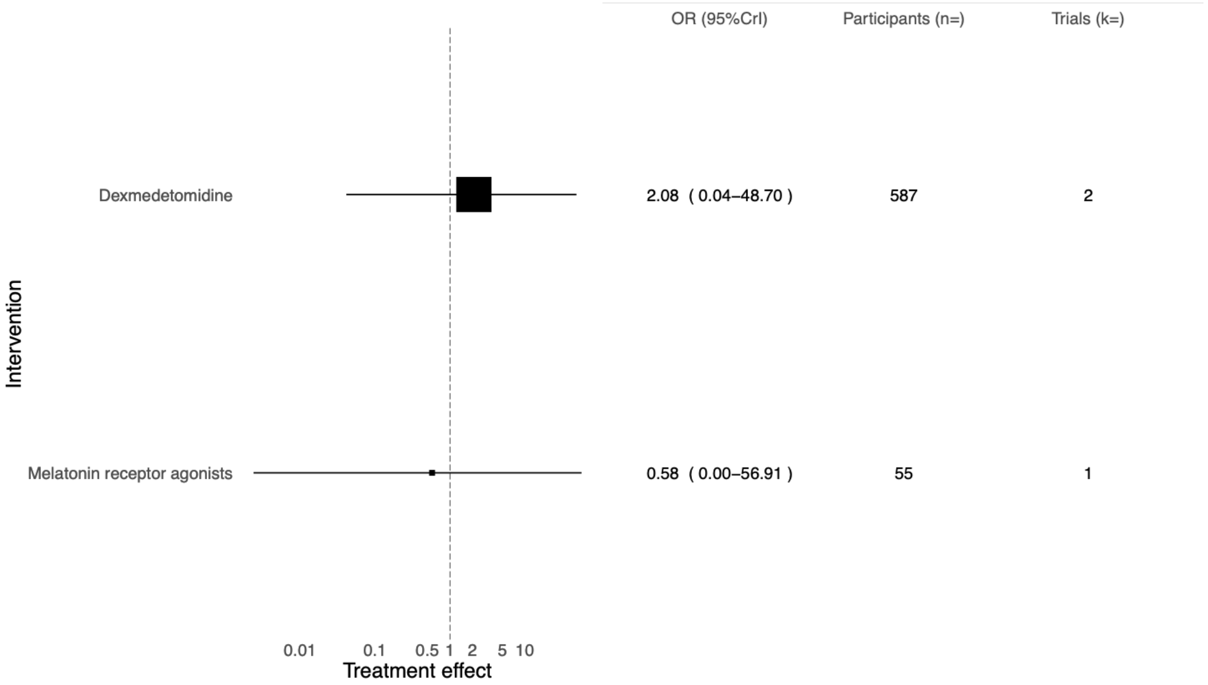

Figure 60 Forest plot of network meta-analysis results for secondary outcome: Complications (arrhythmias)

Sedation

Including drowsiness, somnolence and oversedation

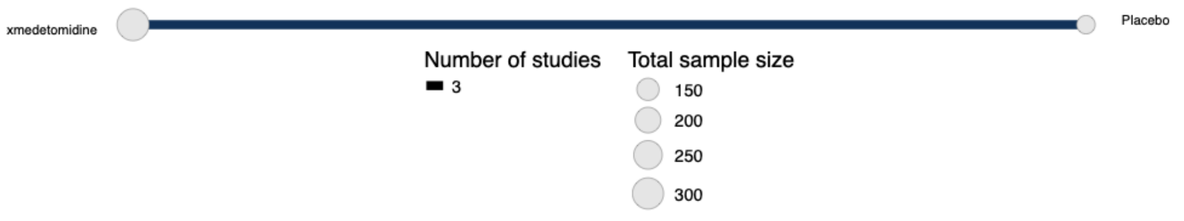

Figure 61 Network graph for the secondary outcome: Complications (sedation)

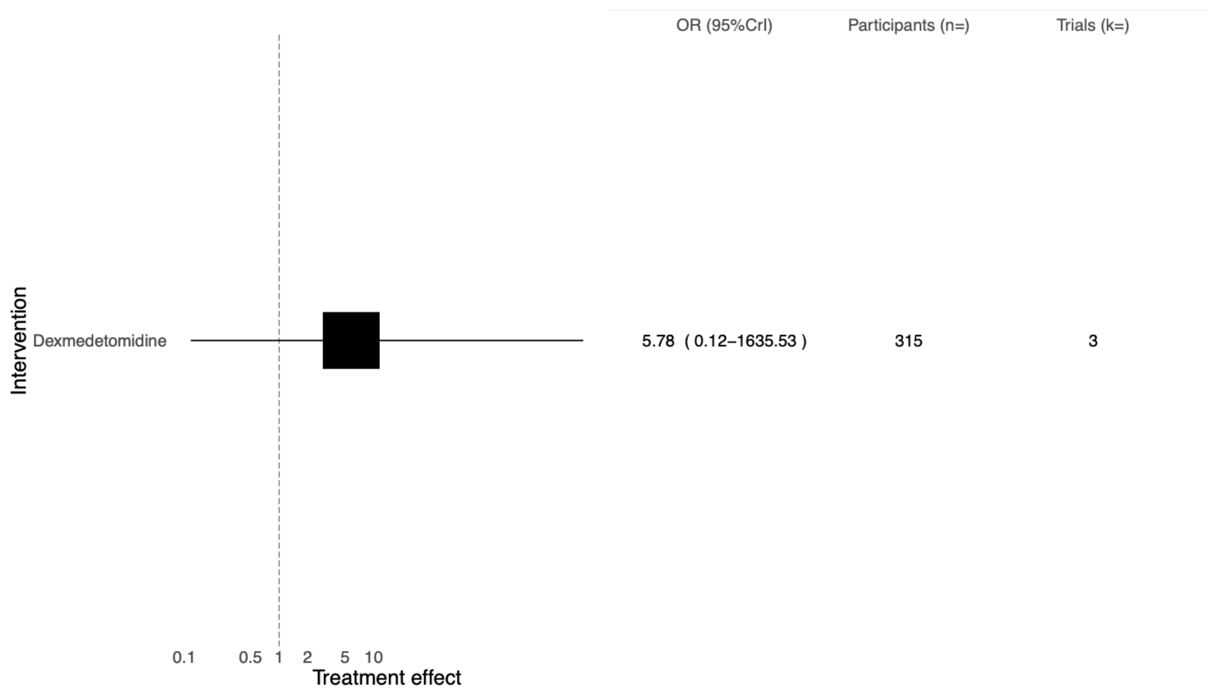

Figure 62 Forest plot of network meta-analysis results for secondary outcome: Complications (arrhythmias)

Tachycardia

Often unspecified, but where specified referred to heart rate >100 beats.min^{-1}.

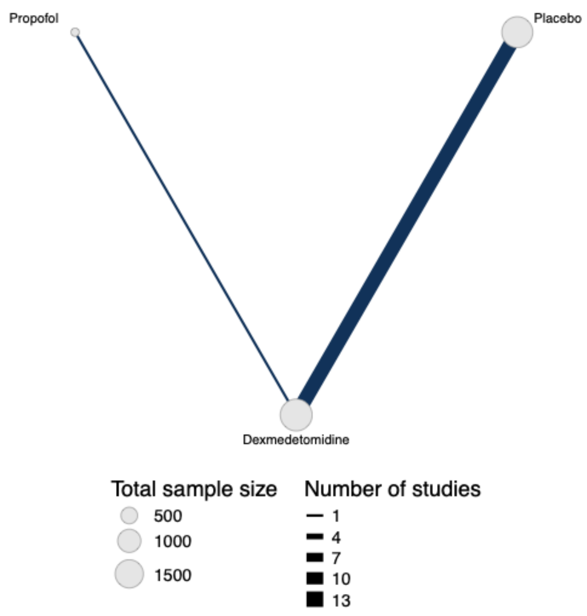

Figure 63 Network graph for the secondary outcome: Complications (tachycardiac)

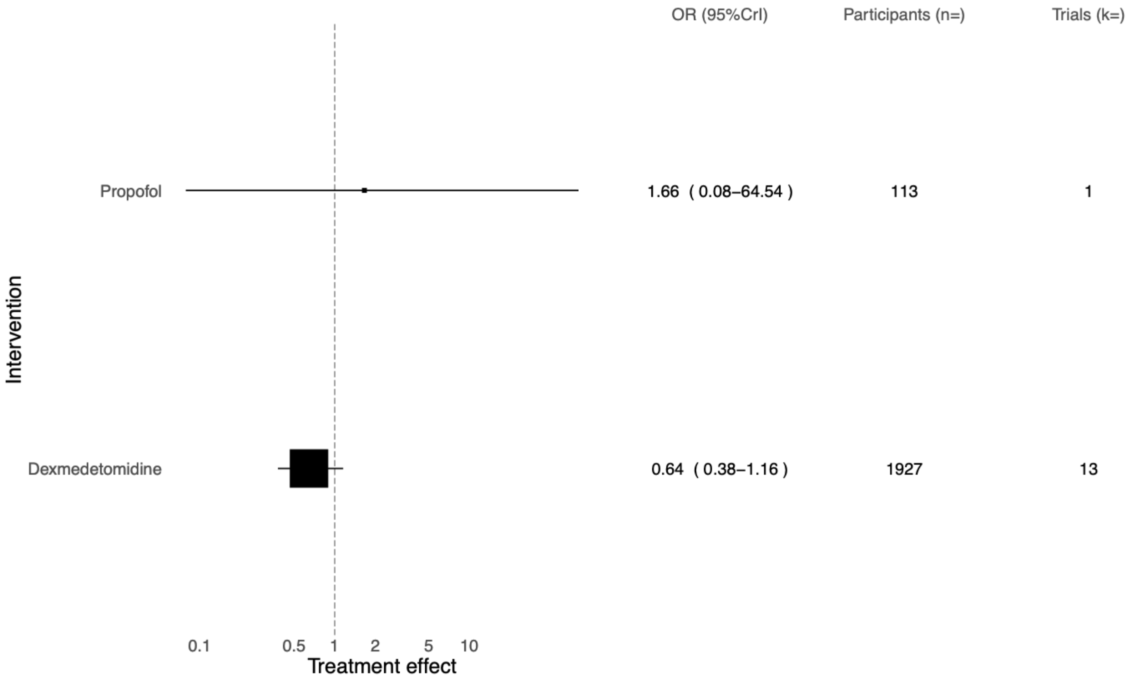

Figure 64 Forest plot of network meta-analysis results for secondary outcome: Complications (arrhythmias)

Extrapyramidal side effects (EPSE)

None of the trials of olanzapine reported on extrapyramidal side effects. EPSE are not applicable to the other interventions (dexmedetomidine, corticosteroids, melatonin receptor agonists parecoxib, nor insulin)

Hypoglycaemia

Huang 2021 (n=90) and Huang 2023 (n=90) both reported on hypoglycaemia in trials of **intranasal** insulin versus saline, however no participants in any allocation were found to have had hypoglycaemia.

Local anaesthetic systemic toxicity (LAST)

Li 2023 (n=106) reported on the incidence of LAST however there were no cases in the lidocaine nor placebo groups. LAST is not applicable to the other interventions that were investigated.

Suspected unexpected severe adverse reaction(s) (SUSAR)

Only one trial reported SUSAR (Beloeil 2021) and reported same rate of SUSAR (5/157, 3.2%) in both treatment arms, dexmedetomidine and remifentanil.

Outcomes with limited data

There were limited data to quantitatively analyse the following secondary outcomes. A narrative summary of the available data are provided below for costs, transfer to institutional care and time to return to original residence.

Cost effectiveness of pharmacological prophylaxis

Only four trials reported any health economic analysis. Djaiani 2016 and Lu 2021 both present robust data on costs associated with dexmedetomidine therapy compared to active control (propofol) and placebo (saline) respectively. Trials by Lv 2022 and Zu 2021, reported some costs but without currency or units the data were unusable. Djaiani 2016 presented mean per patient costs (total inpatient stay) of CAD$8098 for dexmedetomidine arm versus CAD$6058 for propofol arm and Lu 2021 presented median (IQR) inpatient costs of USD$6018 (5051-7035) for dexmedetomidine arm versus USD$6481 (5495-7535) for saline arm. The intervention specific cost for peri-operative dexmedetomidine from Djaiani 2016 was CAD$341.

Transfer to institutional care

Three trials reported on the discharge destination of their participants (Table 29). All were two arm single centre trials, none with an active control, all elective surgery.

Table 29 Studies reporting discharge destination of participants by treatment allocation

Study	Intervention	Total randomised	Intervention n/N (%)		Control n/N (%)	
			Home	Rehabilitation	Home	Rehabilitation
Leung 2006	Nitrous Oxide	210	75/105 (71.4)	39/105 (37.1)	81/105 (77.1)	33/105 (31.4)
Larsen 2010	Olanzapine	495	80/243 (32.9)	116/243 (47.7)	61/252 (24.2)	143/252 (56.7)
Qu 2023	Dexmedetomidine	394	161/188 (85.6)	24/188 (12.8)	167/206 (81.1)	38/206 (18.4)

n, number of participants with that discharge destination; N, number of participants randomized to that arm. Note number of discharged participants won't equal number randomized due to deaths and loss to follow up.

Time to return to original place of residence

As described above only 3 trials report the discharge destinations for participants and none specifically reported how long it took for participants to be discharged to rehabilitation settings to return to their own home. Length of stay data were much more frequently reported – refer to the earlier section on length of stay for this data.

References for supplementary file 1

1. Amin SM, Hasanin A, ElSayed OS, et al. Comparison of the hemodynamic effects of opioid-based versus lidocaine-based induction of anesthesia with propofol in older adults: a randomized controlled trial. *Anaesth Crit Care Pain Med* 2023;42(4):101225. doi: https://dx.doi.org/10.1016/j.accpm.2023.101225
2. Ma PP, Piao MH, Wang YS, et al. Influence of dexmedetomidine and sub-anesthetic dose of ketamine on postoperative delirium in elderly orthopedic patients under total intravenous anesthesia. 2013;39(1):128-32. doi: 10.7694/jldxyxb20130129
3. Jeon Y-G, Kim S, Park J-H, et al. Incidence of intraoperative hypotension in older patients undergoing total intravenous anesthesia by remimazolam versus propofol: A randomized controlled trial. *Medicine (Baltimore)* 2023;102(49):e36440. doi: https://dx.doi.org/10.1097/MD.0000000000036440
4. Beloeil H, Garot M, Lebuffe G, et al. Balanced Opioid-free Anesthesia with Dexmedetomidine versus Balanced Anesthesia with Remifentanil for Major or Intermediate Noncardiac Surgery. *Anesthesiology* 2021;134(4):541-51. doi: https://dx.doi.org/10.1097/ALN.0000000000003725
5. Greenberg S, Murphy GS, Avram MJ, et al. Postoperative Intravenous Acetaminophen for Craniotomy Patients: A Randomized Controlled Trial. *World Neurosurg* 2018;109(101528275):e554-e62. doi: https://dx.doi.org/10.1016/j.wneu.2017.10.021
6. Higgins JT, J; Chandler, J; Cumpston, M; Li, T; Page, MJ; Welch, VA (editors);. Cochrane Handbook for Systematic Reviews of Interventions (updated February 2022). Available from wwwtrainingcochraneorg/handbook: Cochrane, 2022.
7. Xu C, Li L, Lin L, et al. Exclusion of studies with no events in both arms in meta-analysis impacted the conclusions. *J Clin Epidemiol* 2020;123:91-99. doi: 10.1016/j.jclinepi.2020.03.020 [published Online First: 20200401]
8. Cheng J, Pullenayegum E, Marshall JK, et al. Impact of including or excluding both-armed zero-event studies on using standard meta-analysis methods for rare event outcome: a simulation study. *BMJ Open* 2016;6(8):e010983. doi: 10.1136/bmjopen-2015-010983 [published Online First: 20160816]
9. Spence J, Belley-Cote E, Jacobsohn E, et al. Restricted versus liberal intraoperative benzodiazepine use in cardiac anaesthesia for reducing delirium (B-Free Pilot): a pilot, multicentre, randomised, cluster crossover trial. *Br J Anaesth* 2020;125(1):38-46. doi: https://dx.doi.org/10.1016/j.bja.2020.03.030
10. Turner RM, White IR, Croudace T, et al. Analysis of cluster randomized cross-over trial data: a comparison of methods. *Stat Med* 2007;26(2):274-89. doi: 10.1002/sim.2537
11. Forbes AB, Akram M, Pilcher D, et al. Cluster randomised crossover trials with binary data and unbalanced cluster sizes: application to studies of near-universal interventions in intensive care. *Clin Trials* 2015;12(1):34-44. doi: 10.1177/1740774514559610 [published Online First: 20141204]
12. Turner RM, Davey J, Clarke MJ, et al. Predicting the extent of heterogeneity in meta-analysis, using empirical data from the Cochrane Database of Systematic Reviews. *Int J Epidemiol* 2012;41(3):818-27. doi: 10.1093/ije/dys041 [published Online First: 20120329]

13. Zhang W, Wang T, Wang G, et al. Effects of Dexmedetomidine on Postoperative Delirium and Expression of IL-1beta, IL-6, and TNF-alpha in Elderly Patients After Hip Fracture Operation. *Front Pharmacol* 2020;11(101548923):678. doi: https://dx.doi.org/10.3389/fphar.2020.00678
14. Clemmesen CG, Lunn TH, Kristensen MT, et al. Effect of a single pre-operative 125 mg dose of methylprednisolone on postoperative delirium in hip fracture patients; a randomised, double-blind, placebo-controlled trial. *Anaesthesia* 2018;73(11):1353-60. doi: https://dx.doi.org/10.1111/anae.14406
15. Huang J-W, Yang Y-F, Gao X-S, et al. A single preoperative low-dose dexamethasone may reduce the incidence and severity of postoperative delirium in the geriatric intertrochanteric fracture patients with internal fixation surgery: an exploratory analysis of a randomized, placebo-controlled trial. *J ORTHOP SURG* 2023;18(1):441. doi: https://dx.doi.org/10.1186/s13018-023-03930-2
16. Kluger MT, Skarin M, Collier J, et al. Steroids to reduce the impact on delirium (STRIDE): a double-blind, randomised, placebo-controlled feasibility trial of pre-operative dexamethasone in people with hip fracture. *Anaesthesia* 2021;76(8):1031-41. doi: https://dx.doi.org/10.1111/anae.15465
17. de Jonghe A, van Munster BC, Goslings JC, et al. Effect of melatonin on incidence of delirium among patients with hip fracture: a multicentre, double-blind randomized controlled trial. *CMAJ* 2014;186(14):E547-56. doi: https://dx.doi.org/10.1503/cmaj.140495
18. Oh ES, Leoutsakos J-M, Rosenberg PB, et al. Effects of Ramelteon on the Prevention of Postoperative Delirium in Older Patients Undergoing Orthopedic Surgery: The RECOVER Randomized Controlled Trial. *Am J Geriatr Psychiatry* 2021;29(1):90-100. doi: https://dx.doi.org/10.1016/j.jagp.2020.05.006
19. Larsen KA, Kelly SE, Stern TA, et al. Administration of olanzapine to prevent postoperative delirium in elderly joint-replacement patients: a randomized, controlled trial. *Psychosomatics* 2010;51(5):409-18. doi: https://dx.doi.org/10.1176/appi.psy.51.5.409
20. Salanti G, Ades AE, Ioannidis JP. Graphical methods and numerical summaries for presenting results from multiple-treatment meta-analysis: an overview and tutorial. *J Clin Epidemiol* 2011;64(2):163-71. doi: 10.1016/j.jclinepi.2010.03.016 [published Online First: 20100805]
21. Mbuagbaw L, Rochwerg B, Jaeschke R, et al. Approaches to interpreting and choosing the best treatments in network meta-analyses. *Syst Rev* 2017;6(1):79. doi: 10.1186/s13643-017-0473-z [published Online First: 20170412]
22. McGuinness LA, Higgins JPT. Risk-of-bias VISualization (robvis): An R package and Shiny web app for visualizing risk-of-bias assessments. *Res Synth Methods* 2021;12(1):55-61. doi: 10.1002/jrsm.1411 [published Online First: 20200506]
23. Nikolakopoulou A, Higgins JPT, Papakonstantinou T, et al. CINeMA: An approach for assessing confidence in the results of a network meta-analysis. *PLoS Med* 2020;17(4):e1003082. doi: 10.1371/journal.pmed.1003082 [published Online First: 20200403]
24. Papakonstantinou T, Nikolakopoulou A, Higgins JPT, et al. CINeMA: Software for semiautomated assessment of the confidence in the results of network meta-

analysis. *Campbell Syst Rev* 2020;16(1):e1080. doi: 10.1002/cl2.1080 [published Online First: 20200311]
25. Salanti G, Del Giovane C, Chaimani A, et al. Evaluating the quality of evidence from a network meta-analysis. *PLoS One* 2014;9(7):e99682. doi: 10.1371/journal.pone.0099682 [published Online First: 20140703]
26. Gross AL, Tommet D, D'Aquila M, et al. Harmonization of delirium severity instruments: a comparison of the DRS-R-98, MDAS, and CAM-S using item response theory. *BMC Med Res Methodol* 2018;18(1):92. doi: 10.1186/s12874-018-0552-4 [published Online First: 20180910]
27. Wan X, Wang W, Liu J, et al. Estimating the sample mean and standard deviation from the sample size, median, range and/or interquartile range. *BMC Med Res Methodol* 2014;14:135. doi: 10.1186/1471-2288-14-135 [published Online First: 20141219]
28. Moller JT, Cluitmans P, Rasmussen LS, et al. Long-term postoperative cognitive dysfunction in the elderly: ISPOCD1 study. *The Lancet* 1998;351(9106):857-61. doi: 10.1016/s0140-6736(97)07382-0
