# Supplementary material for: Effectiveness of drug interventions to prevent delirium after surgery for older adults: systematic review and network meta-analysis of randomised controlled trials
Source: BMJ. 2026 Feb 12;392:e085539. doi: 10.1136/bmj-2025-085539 (PMC12895303; doi:10.1136/bmj-2025-085539)
Supplement: Supplementary file 2 — Web appendix: Supplementary file 2 [file lunm085539.ww2.pdf]

Supplementary file 2. Included studies

There were 41084 participants included from 158 studies. The overall incidence of postoperative delirium was 14.5% (n=5957/41084). Across the placebo arms the overall incidence of delirium was 17.1% (n=2665/15565). Among active interventions the overall incidence of delirium was 12.9% (3292/25519). Of the 337 comparisons, the majority were administered intravenously 74.7% (n=252), 16.0% (n=54) orally, and 6.8% (n=23) inhaled.

Table 1. Summary of trial characteristics

Study	sample size	country	Intervention Arm	Int. events/ randomized (%)	Control Arm	Control events/ randomized (%)	Timing of intervention (all on day of surgery)	Mode of administration	Assessment tool	Postoperative ICU care (%)
Abd Ellatif 2024[1]	120	Egypt	Arm 1: Dexmedetomidine 0.2mcg/kg/h<br>Arm 3: Propofol 0.3-0.4mg/kg/h + Ketamine 0.125mg/kg/h	Arm1: 1/40 (2.5)<br>Arm 3: 2/40 (5)	Equivolume saline	7/40 (17.5)	During surgery	IV	CAM-ICU	100
Aizawa 2002[2]	40	Japan	Diazepam 0.1mg/kg intramuscular +<br>Flunitrazepam 0.04mg/kg IV infusion<br>Pethidine 1mg/kg IV	1/20 (5)	Usual care	7/20 (35)	After surgery complete	IM/IV	DSM IV	NR
Al Tmimi 2015[3]	42	Belgium	Xenon 50-60% inhaled	2/21 (9.5)	Sevoflurane 1-1.4%	8/21 (38.1)	During surgery	INH	CAM/CAM-ICU	100*
Al Tmimi 2017[4]	50	Belgium	Xenon 30% inhaled	7/25 (28)	Propofol (Marsh model) TCI to BIS 40-60	2/25 (8)	During surgery	INH/IV	CAM	100*
Al Tmimi 2020[5]	190	Belgium	Xenon inhaled 40-60 % inhaled	41/96 (42.7)	Sevoflurane inhaled 1-1.4 %	37/94 (39.4)	During surgery	INH	3D-CAM/CAM-ICU	100*
Avidan 2017[6]	672	USA, Canada, South	Arm 1: Ketamine 1mg/kg. Arm 3: Ketamine 0.5mg/kg	Arm 1: 46/223 (19.7)<br>Arm 3: 39/227	Equivolume saline	43/222 (19.4)	During surgery	IV	CAM/CAM-ICU	100*

		Korea, India		(17.2)						
Awada 2022[7]	63	Denmark	Methylprednisolone 10 mg/kg i.v. infused in 100 ml 0.9% saline	0/33 (0)	Dexamethasone 8mg IV	5/30 (16.7)	During surgery	IV	3DCAM	NR
Azeem 2018[8]	70	Egypt	Dexmedetomidine 1mcg/kg loading and infusion (0.4–0.7 µg/kg/h)	1/35 (2.9)	Morphine 10–50 µg/kg/h + midazolam 0.05 mg/kg to 0.2 mg/kg PRN	2/35 (5.7)	After surgery complete	IV	CAM-ICU	100*
Beloeil 2021[9]	314	France	Dexmedetomidine IV infusion 0.4-1.4mcg/kg/h	2/157 (1.3)	Remifentanil Cet IV infusion TCI 3-5ng/ml (0.1-0.25mcg/kg/min	0/157 (0)	During surgery	IV	CAM-ICU	NR
Bielza 2021[10]	152	Spain	Iron 200mg intravenous	16/74 (21.6)	Equivolume saline	17/78 (21.8)	Prior to start of surgery	IV	CAM	NR
Cao 2023[11]	1228	China	Propofol target-controlled infusion	50/614 (8.1)	Sevoflurane inhalation	74/614 (12.1)	During surgery	INH/IV	CAM/CAM-ICU	25.0
Chen 2021[12]	160	Taiwan	Dexmedetomidine 0.5mcg/kg/h	7/80 (8.8)	Equivolume saline	14/80 (17.5)	During surgery	IV	ICDSC	100
Chitnis 2022[13]	70	Canada	Dexmedetomidine IV infusion starting 0.5 mg/kg/h, titrated 0 to 1.5 mg/kg/h	8/35 (22.9)	Propofol 25 to 50 mg/kg/min	14/35 (40)	During surgery	IV	ICDSC	100*
Choovongkomol 2024[14]	200	Thailand	Dexmedetomidine 0.3-0.5mcg/kg	18/100 (18)	Equivolume saline	40/100 (40)	During surgery	IV	CAM/CAM-ICU	NR
Clemmesen 2018[15]	120	Denmark	Methylprednisolone 125mg intravenous injection	10/60 (16.7)	Equivolume saline	19/60 (31.7)	Prior to start of surgery	IV	CAM	NR
Coburn 2018[16]	256	France, Belgium, Germany, Spain, UK, Italy	Xenon 50% (MAC1)	12/124 (9.7)	sevoflurane 1.1-1.4% (MAC1)	18/132 (13.6)	During surgery	INH	CAM	NR
de Jonghe 2014[17]	395	Netherlands	Melatonin 3mg nocte	55/196 (28.1)	Placebo tablet	49/199 (24.6)	Prior to start of surgery	PO	DSMIV	Excluded ICU patients
Deiner 2017[18]	390	USA	Dexmedetomidine infusion (0.5 µg/kg/h) during surgery and up to 2 hours in the recovery room.	23/189 (12.2)	Equivolume saline	23/201 (11.4)	During surgery	IV	CAM/CAM-ICU	Excluded ICU patients

Deng 2021[19]	248	China	Methylene blue IV continuous infusion 2 mg/kg in 50ml saline	9/124 (7.3)	Equivolume saline	30/124 (24.2)	During surgery	IV	3DCAM	NR
Djaiani 2016[20]	183	Canada	Dexmedetomidine 0.4 µg/kg bolus followed by 0.2 to 0.7 µg/kg/h infusion	16/91 (17.6)	Propofol 25-50 µg/kg/min iv infusion	29/92 (31.5)	After surgery complete	IV	CAM-ICU	100*
Duan 2023[21]	296	China	Sevoflurane (1.0–1.5 MAC)	35/148 (23.6)	Propofol (2.0–3.0 mg/kg/h)	35/148 (23.6)	During surgery	INH/IV	ICDSC	100*
Duan 2024[22]	106	China	Remimazolam 0.05 mg/kg IV bolus, then 0.1-0.3 mg·kg-1·h-1 infusion	4/53 (7.5)	slow intravenous injection of propofol 0.3-0.5 mg/kg over 1 minute, followed by a maintenance dose of 0.5-3 mg·kg-1·h-1	15/53 (28.3)	During surgery	IV	CAM	NR
Farrer 2023[23]	199	USA	TIVA propofol plus fentanyl	13/100 (13)	Isoflurane (plus fentanyl) BIS 40-60	14/99 (14.1)	During surgery	INH/IV	CAM	NR
Fazel 2022[24]	80	Iran	Melatonin 5mg PO	8/40 (20)	Placebo tablet	16/40 (40)	Prior to start of surgery	PO	AMT	NR
Ford 2020[25]	207	Australi a	Melatonin 3mg capsules	21/100 (21)	Placebo capsule	21/105 (20)	Prior to start of surgery	PO	CAM	NR
Gajniak 2023[26]	70	Poland	lidocaine bolus of 1.5 mg/kg, followed by continuous infusion of 1–3 mg/kg/h intraoperatively, and the dose of 1–3 mg/kg/h maintained for 24–48 h after surgery	1/33 (3)	Equivolume saline	6/37 (16.2)	During surgery	IV	CAM	2.9
Gamberini 2009[27]	113	Switzerl and	Rivastigmine 1.5 mg oral TDS	18/57 (31.6)	Placebo tablet	17/56 (30.4)	Prior to start of surgery	PO	CAM	100*
Gao 2020[28]	60	China	Dexmedetomidine 1mcg/kg bolus, 0.3-0.5mcg/kg/h infusion	3/30 (10)	Equivolume saline	9/30 (30)	During surgery	IV	APA	100*
Gao 2021[29]	40	China	Dexmedetomidine loading dose0.6µg/kg , continuous infusion 0.2µg/kg/h	3/20 (15)	Equivolume saline	10/20 (50)	During surgery	IV	DSM IV	100*
Ghazaly 2023[30]	60	Egypt	Arm 1: Dexmedetomidine 1mcg/kg, Arm 3: Ketamine 1mg/kg	Arm1: 1/20 (5) Arm 3: 2/20 (10)	Equivolume saline	15/20 (75)	During surgery	IV	DOS	Exclude d ICU patients
Greenberg 2018[31]	140	USA	Acetaminophen 1000 mg IV	1/70 (1.4)	Equivolume saline	0/70 (0)	After surgery	IV	CAM-ICU	NR

							complete			
Guo 2015[32]	156	China	Dexmedetomidine 0.2mcg/kg/h	6/78 (7.7)	Equivolume saline	21/78 (26.9)	After surgery complete	IV	CAM-ICU	100
Gupta 2019[33]	100	India	Ramelteon 8mg tablet oral	2/50 (4)	Placebo tablet	6/50 (12)	Prior to start of surgery	PO	CAM	NR
Haller 2021[34]	140	Hong Kong, Switzerland	N2O 70%, 30% O2	0/72 (0)	Air 70%, 30% O2	1/68 (1.5)	During surgery	INH	DSM4	12.1
Harmon 2004[35]	36	Ireland	Aprotinin 2 × 10^6 KIU (loading dose), 2 × 10^6 KIU (added to circuit prime) and a continuous infusion of 5 × 10^5 KIU·hr–1	1/18 (5.6)	Placebo injection	3/18 (16.7)	During surgery	IV	DSM-III-R criteria,	100*
He 2021[36]	60	China	Dexmedetomidine 0.1 μg/kg/hour IV infusion	2/30 (6.7)	Equivolume saline	3/30 (10)	After surgery complete	IV	CAM-ICU	100
He 2022[37]	80	China	Dexmedetomidine 1mcg/kg bolus, 0.5mcg/kg/h infusion	3/40 (7.5)	Equivolume saline	10/40 (25)	During surgery	IV	CAM	Excluded ICU patients
He 2023[38]	60	China	Dexmedetomidine 0.5mcg/kg intravenous bolus, 0.2mcg/kg/h infusion	3/30 (10)	Equivolume saline	13/30 (43.3)	During surgery	IV	CAM	NR
He 2024[39]	120	China	Arm 1: Alprazolam 0.4mg PO, Arm 3: Dexmedetomidine 1.5mcg/kg IN	Arm1: 15/40 (37.5)<br>Arm 3: 5/40 (12.5)	Equivolume saline	13/40 (32.5)	Prior to start of surgery	IV	CAM	NR
Hollinger 2021[40]	182	Switzerland	Arm 1: Haloperidol 5mcg/kg IV,<br>Arm 3: Ketamine 1mg/kg IV,<br>Arm 4: Haloperidol 5mcg/kg + Ketamine 1mg/kg IV	Arm 1: 7/45 (15.6)<br>Arm 3: 10/47 (21.3)<br>Arm 4: 3/46 (6.5)	Placebo injection	6/44 (13.6)	During surgery	IV	DOS/NuDESC / ICDSC	Excluded ICU patients
Hong 2021[41]	712	China	Dexmedetomidine 2.5mcg IV bolus PCA lockout 8 min, background infusion of 1.25mcg/h	17/356 (4.8)	Equivolume saline	26/356 (7.3)	After surgery complete	IV	CAM/CAM-ICU	2.0
Hongyu 2019[42]	90	China	Arm 1: Penehyclidine 10mcg/kg IM, Arm 3: Atropine 10mcg/kg	Arm 1: 16/30 (53.3)<br>Arm 3: 7/30 (23.3)	Equivolume saline	5/30 (16.7)	During surgery	IV	CAM	NR
Hu 2021[43]	177	China	Dexmedetomidine 0.4mcg/kg bolus over 15 minutes before induction, 0.1mcg/kg/h infusion	15/90 (16.7)	Equivolume saline	32/87 (36.8)	During surgery	IV	CAM	Excluded ICU

			until 1h before end of surgery							patients
Hu 2022[44]	60	China	Arm 1: Dexmedetomidine 0.04 mg/mL intravenous bolus, infusion 0.4 µg/kg/h, Arm 3: Dexmedetomidine intravenous bolus 0.04 mg/mL, infusion 0.4 µg/kg/h+40mg atorvastatin daily	Arm 1: 1/20 (5) Arm 3: 1/20 (5)	Equivolume saline	3/20 (15)	During surgery	IV	CAM criteria	NR
Huang 2021[45]	90	China	Insulin 20 units intranasal	5/45 (11.1)	Equivolume saline	19/45 (42.2)	Prior to start of surgery	IV	CAM-ICU	NR
Huang 2023[46]	160	China	Dexamethasone 10mg intravenous bolus	9/80 (11.2)	Equivolume saline	21/80 (26.2)	Prior to start of surgery	IV	CAM-ICU	100
Huang 2023[47]	90	China	Arm 1: Insulin 20 units intranasal. Arm 3: Insulin 30 units intranasal	Arm1 : 9/30 (10) Arm 3: 1/30 (3)	Equivolume saline	19/30 (63.3)	During surgery	IV	Nu-DESC	NR
Hudetz 2009[48]	58	USA	Ketamine 0.5 mg/kg iv bolus	1/29 (3.4)	Equivolume saline	9/29 (31)	During surgery	IV	ICDSC	100*
Huet 2024[49]	333	France	Dexmedetomidine intravenous 0.1-1.4mcg/kg/h Infusion	20/166 (12)	Equivolume saline	20/167 (12)	After surgery complete	IV	CAM-ICU	100*
Ishii 2016[50]	59	Japan	Propofol 1.5-3 µg/mL Cet TCI	2/29 (6.9)	Sevoflurane with 1 to 1.5 minimum alveolar concentration	8/30 (26.7)	During surgery	INH/IV	CAM-ICU	100
Jaiswal 2019[52]	117	USA	Ramelteon 8mg tablet oral	19/59 (32.2)	Placebo tablet	22/58 (37.9)	Prior to start of surgery	PO	CAM-ICU	100*
Javaherforooshzadeh 2021[53]	60	Iran	Melatonin 3mg tablet	4/30 (13.3)	Placebo tablet	11/30 (36.7)	Prior to start of surgery	PO	CAM-ICU	100*
Javaherforooshzadeh 2023[54]	80	Iran	Melatonin 3mg tablet	6/40 (15)	Placebo tablet	12/40 (30)	Prior to start of surgery	PO	CAM/CAM-ICU	100*
Jeon 2023[55]	132	South Korea	Remimazolam 6mg/kg/h induction, 1-2mg/kg/h maintenance BIS 50	0/66 (0)	Propofol (Schneider 4mcg/ml Cet) induction, 2.5-4mcg/ml maintenance BIS 50	0/66 (0)	During surgery	IV	DSM V	0.8
Jiang 2023[51]	698	China	Sevoflurane or desflurane minimum Et conc 0.5 - 2 MAC inhaled	76/349 (21.8)	Propofol 3-8mg/kg/h	63/349 (18.1)	During surgery	INH/IV	CAM-ICU	100*
Kalisvaart	430	Netherla	0.5mg Haloperidol PO	32/212 (15.1)	placebo_tablet	36/218 (16.5)	After	PO	CAM/D	NR

2005[56]		nds					surgery complete		SM IV	
Kanamori 2023[57]	64	Japan	Magnesium 30mg/kg iv bolus (1h), 10mg/kg/h infusion (max 3h)	2/31 (6.5)	Equivolume saline	2/33 (6.1)	During surgery	IV	CAM-ICU	100
Kaneko 1999[58]	80	Japan	Haloperidol 5mg IV	4/40 (10)	Equivolume saline	13/40 (32.5)	After surgery complete	IV	DSM III-R	NR
Khan 2018[59]	135	USA	Haloperidol 0.5mg iv bolus	15/68 (22.1)	Equivolume saline	19/67 (28.4)	After surgery complete	IV	CAM/CAM-ICU	100
Kim 2019[60]	143	South Korea	Dexmedetomidine 0.5mcg/kg/h, BIS 45 +/-5	15/73 (20.5)	Equivolume saline	15/70 (21.4)	During surgery	IV	CAM/CAM-ICU	100
Kinouchi 2023[61]	108	Japan	ramelteon 8mg tablet oral	7/55 (12.7)	Placebo tablet	4/53 (7.5)	Prior to start of surgery	PO	CAM-ICU	NR
Kluger 2021[62]	79	New Zealand	Dexamethasone 20 mg intravenous injection	6/40 (15)	Equivolume saline	9/39 (23.1)	Prior to start of surgery	IV	4AT	NR
Lai 2023[63]	90	China	Arm 1: Dexmedetomidine 1 mcg/kg 10 minute IV bolus, continuous infusion 0.5 mcg/kg/h.<br>Arm 3: Lidocaine 1mg/kg bolus, 1mg/kg lidocaine infusion	Arm 1: 5/30 (16.7)<br>Arm 3: 9/30 (30)	Equivolume saline	4/30 (13.3)	During surgery	IV	3DCAM	NR
Larsen 2010[64]	495	USA	Olanzapine 5mg PO	28/243 (11.5)	Placebo tablet	82/252 (32.5)	Prior to start of surgery	PO	CAM/DSM-III-R	NR
Lee 2018[65]	345	South Korea	Arm 1: Dexmedetomidine 1 µg/kg bolus followed by 0.2 to 0.7 µg/kg/h infusion.<br>Arm 3: Dexmedetomidine 1 µg/kg bolus	Arm 1: 9/111 (8.1)<br>Arm 3: 21/118 (17.8)	Equivolume saline	27/116 (23.3)	During surgery	IV	CAM	17.7
Lee 2020[66]	215	South Korea	Dexmedetomidine 0.1mcg/kg/h infusion	9/107 (8.4)	Equivolume saline	6/108 (5.6)	During surgery	IV	CAM-ICU	100
Leung 2006[67]	210	USA	N2O with O2	44/105 (41.9)	O2 (with or without air)	46/105 (43.8)	During surgery	INH	CAM	NR
Leung 2006[68]	21	USA	Gabapentin 900mg PO	0/9 (0)	Placebo tablet	5/12 (41.7)	Prior to start of surgery	PO	CAM	NR

Leung 2017[69]	697	USA	Gabapentin 900mg PO	84/350 (24)	Placebo tablet	75/347 (21.6)	Prior to start of surgery	PO	CAM	NR
Li 2013[70]	80	China	Parecoxib sodium 40 mg intravenously (reduce to 20mg if <50kg)	9/40 (22.5)	Morphine 2/4mg then saline	18/40 (45)	After surgery complete	IV	DSM IV	NR
Li 2017[71]	285	China	Dexmedetomidine iv bolus 0.6 μg/kg for 10 minutes, 0.4 μg/k/h intraop, 0.1mcg.kg.h postop until extubation	7/142 (4.9)	Equivolume saline	11/143 (7.7)	During surgery	IV	CAM/CAM-ICU	100*
Li 2020[72]	619	China	Dexmedetomidine (0·6 μg/kg) bolus, infusion 0·125 ml per kg per h (0·5 μg per kg per h)	17/309 (5.5)	Equivolume saline	32/310 (10.3)	During surgery	IV	CAM/CAM-ICU	14.7
Li 2023[73]	106	China	1% Lidocaine 1 mg/kg (0.15 mL/kg) bolus, 0.15 mL/(kg·h) until the end of surgery	7/53 (13.2)	Equivolume saline	18/53 (34)	During surgery	IV	CAM	NR
Li 2023[74]	260	China	Dexmedetomidine 0.6 mg/k 10 minute IV bolus, continuous infusion then 0.4 mg/kg/h	28/130 (21.5)	Equivolume saline	60/130 (46.2)	During surgery	IV	CAM-ICU	16.2
Likhvantsev 2021[75]	175	Russia	Dexmedetomidine 0.7mcg/kg/h intraop, titrated 0.4-1.4mcg/kg/h in ICU	6/87 (6.9)	Equivolume saline	16/88 (18.2)	During surgery	IV	CAM-ICU	100*
Lin 2022[76]	170	China	Hydrogen FiH2 0.667	10/85 (11.8)	Oxygen FiO2 0.33	17/85 (20)	Prior to start of surgery	INH/IV	CAM	NR
Liu 2016[77]	61	China	Dexmedetomidine (0.2-1.5 μg/kg/h) iv infusion	0/33 (0)	Propofol (5-50 μg/kg/min)	2/34 (5.8)	During surgery	IV	CAM	1.5
Liu 2016[78]	197	China	Dexmedetomidine 0.2–0.4 mcg/kg/h	15/99 (15.2)	Equivolume saline	43/98 (43.9)	After surgery complete	IV	CAM-ICU	100*
Liu 2021[79]	120	China	Dexmedetomidine 0.5 mcg/kg 10 minute IV bolus, continuous infusion then 0.4 mcg/kg/h	5/60 (8.3)	Equivolume saline	8/60 (13.3)	During surgery	IV	3DCAM	Excluded ICU patients
Liu 2023[80]	304	China	Dexmedetomidine 0.5 μg.kg−1 bolus, maintenance infusion 0.2 μg.kg−1.hr−1	32/152 (21.1)	Equivolume saline	36/152 (23.7)	During surgery	IV	CAM	NR
Liu 2024[81]	105	China	Ciprofol 0.3-0.4mg/kg induction, 1-2.4mg/kg/h maintenance, BIS 40-60	3/53 (5.7)	Propofol 1.0–1.5 mg/kg bolus, 4-12mg/kg/h infusion maintenance, BIS 40-	7/52 (13.5)	During surgery	IV	CAM	NR

					60					
Long 2024[82]	78	China	Dexmedetomidine 0.5 µg/kg/h intravenous infusion	3/39 (7.7)	Equivolume saline	7/39 (17.9)	During surgery	IV	3DCAM /CAM-ICU	3.8
Lu 2021[83]	808	China	Dexmedetomidine bolus 0.5 µg/kg IV, maintenance 0.2 µg/kg per hour	41/404 (10.1)	Equivolume saline	43/404 (10.6)	During surgery	IV	CAM	NR
Lu 2023[84]	94	China	Dexmedetomidine 0.7 µg/kg IV bolus, 0.2-0.5 µg/kg/h infusion	9/47 (19.1)	Esketamine 0.5 mg/kg IV	2/47 (4.3)	During surgery	IV	CAM	NR
Lurati Buse 2012[85]	385	Switzerland	propofol	29/201 (14.4)	Sevoflurane inhaled	21/184 (11.4)	During surgery	INH/IV	CAM	NR
Lv 2022[86]	327	China	Dexmedetomidine 0.1 µg/kg/h	21/163 (12.9)	Equivolume saline	46/164 (28)	After surgery complete	IV	CAM	100
Ma 2013[87]	120	China	Arm 1: Dexmedetomidine 1mcg/kg, infusion 0.5mcg/kg/h. Arm 3: Ketamine 0.5mg/kg, Arm 4: Dexmedetomidine 1mcg/kg, infusion 0.5mcg/kg/h + Ketamine 0.5mg/kg	Arm 1: 2/30 (6.7)<br>Arm 3: 8/30 (26.7)<br>Arm 4: 0/30 (0)	Equivolume saline	3/30 (10)	During surgery	IV	CAM	NR
Ma 2023[88]	62	China	Esketamine 0.25 mg/kg induction; 0.125 mg/kg/h maintenance	3/31 (9.7)	Equivolume saline	4/31 (12.9)	During surgery	IV	CAM-ICU	Excluded ICU patients
Mahrose 2021[89]	110	Egypt	Melatonin tablet 5 mg	6/55 (10.9)	Usual care	15/55 (27.3)	Prior to start of surgery	PO	CAM/CAM-ICU	100*
Maldonado 2009[90]	118	USA	Arm 1: Dexmedetomidine 0.4 µg/kg bolus followed by 0.2 to 0.7 µg/kg/h infusion<br>Arm 3: Midazolam 0.5-2mg/h	Arm 1: 1/40 (5)<br>Arm 3: 15/40 (37.5)	Propofol 25-50mcg/kg/min	15/38 (39.5)	During surgery	IV	DSM IV-TR	100*
Marcantonio 2011[91]	16	USA	Donepezil 5 mg PO	3/7 (42.9)	Placebo tablet	3/9 (33.3)	Prior to start of surgery	PO	CAM	NR
Mardani 2013[92]	93	Iran	Dexamethasone 8 mg IV	4/43 (9.3)	Equivolume saline	13/50 (26)	During surgery	IV	DSM-IV	100*
Massoudi 2023[93]	100	Iran	Rivastigmine 1.5 mg orally	4/50 (8)	Placebo tablet	12/50 (24)	Prior to start of surgery	PO	CAM	100
Massoumi 2019[94]	90	Iran	Dexmedetomidine 1 µg/kg bolus, infusion of 0.2-0.7 µg/kg/h	4/45 (8.9)	Equivolume saline	9/45 (20)	During	IV	CAM-	100*

							surgery		ICU	
Mei 2018[95]	336	China	Dexmedetomidine 0.8-1mcg/kg bolus over 15-20min, infusion 0.1-0.5mcg/kg/h	11/167 (6.6)	Propofol TCI 0.8-1mcg/ml Cet	24/169 (14.2)	During surgery	IV	CAM	NR
Mei 2020[96]	240	China	Propofol TCI	35/122 (28.7)	Sevoflurane 1%−4%	24/118 (20.3)	During surgery	INH/IV	CAM	NR
Mei 2020[97]	415	China	Dexmedetomidine 0.8-1mcg/kg bolus over 15-20min, infusion 0.1-0.5mcg/kg/h	26/207 (12.6)	Propofol TCI 0.8-1mcg/ml Cet	43/208 (20.7)	During surgery	IV	CAM	NR
Mohamed 2022[98]	80	Egypt	Melatonin 5mg PO	10/40 (25)	Paracetamol 500mg	21/40 (52.5)	Prior to start of surgery	PO	AMT	NR
Mohammadi 2016[99]	45	Iran	Cyproheptadine 4mg TDS orally	3/23 (13)	Placebo tablet	7/22 (31.8)	After surgery complete	PO	CAM-ICU	100
Momeni 2021[100]	417	Belgium	dexmedetomidine 0.4mg/kg/h	31/208 (14.9)	Equivolume saline	33/209 (15.8)	During surgery	IV	CAM-ICU/CAM	100*
Moslemi 2020[101]	96	Iran	Thiamine 200 mg IV	4/48 (8.3)	Equivolume saline	12/48 (25)	After surgery complete	IV	CAM-ICU	100
Mu 2017[102]	620	China	40mg parecoxib iv bolus	19/310 (6.1)	Equivolume saline	34/310 (11)	After surgery complete	IV	CAM/CAM-ICU	6.3
Nishikawa 2004[103]	50	Japan	Propofol 4mg/ml TCI	4/25 (16)	Sevoflurane inhaled	0/25 (0)	During surgery	INH/IV	DRS	NR
Niu 2023[104]	149	China	Arm 1: Dexmedetomidine 0.6mg/kg IV bolus. Arm 3: Dexmedetomidine 0.6mcg/kg endotracheal	Arm 1: 3/49 (6.1) Arm 3: 5/50 (10)	Dexmedetomidine 1mcg/kg intranasal	14/50 (28)	During surgery	INH/IV/IN	3DCAM	Excluded ICU patients
Oh 2021[105]	80	USA	8 mg Ramelteon orally	3/41 (7.3)	Placebo tablet	2/39 (5.1)	Prior to start of surgery	PO	CAM/DSM V	0.0
Papadopoulos 2014[106]	106	Greece	Ondansetron 8mg intravenous bolus	18/51 (35.3)	Placebo injection	29/55 (52.7)	After surgery complete	IV	CAM	NR
Park 2014[107]	142	South Korea	Dexmedetomidine iv bolus (0.5 μg/kg) then infusion 0.2 -0.8 μg/kg/hr	6/67 (9)	Remifentanil 1-2.5ng/hr	17/75 (22.7)	After surgery complete	IV	CAM-ICU	100*

Prakanrattana 2007[108]	126	Thailand	Risperidone 1mg (oral dissolving) tablet	7/63 (11.1)	Dissolving sublingual placebo strip	20/63 (31.7)	After surgery complete	PO	CAM-ICU	100*
Preveden 2023[109]	120	Serbia	Dexmedetomidine infusion in doses 0.2-0.7 mcg/kg/h	7/60 (11.7)	Propofol infusion in doses 1-2 mg/kg/h.	15/60 (25)	After surgery complete	IV	CAM-ICU	100*
Qi 2023[110]	62	China	Esketamine 0.3mg/kg intravenous bolus, 0.25mg/kg/h infusion	2/31 (6.5)	Equivolume saline	7/31 (22.6)	During surgery	IV	CAM	NR
Qu 2023[111]	394	USA	Dexmedetomidine (1 µg/kg over 40 min), maximal dose of 80 µg	5/188 (2.7)	Equivolume saline	16/206 (7.8)	After surgery complete	IV	CAM	100*
Robinson 2014[112]	301	USA	L-tryptophan 1 gram enterally	61/152 (40.1)	Placebo tablet	55/149 (36.9)	After surgery complete	PO	CAM-ICU	100*
Royse 2011[113]	182	Australi a	Propofol 1.5-3 mcg.ml TCI induction Marsh, BIS40-60 maintenance	7/91 (7.7)	Sevoflurane induction, Desflurane maintenance BIS40-60	12/91 (13.2)	During surgery	INH/IV	CAM	100*
Sakic 2023[114]	60	Croatia	Dexamethasone 8mg intrathecal	1/30 (3.3)	Equivolume saline	5/30 (16.7)	During surgery	IT	CAM	NR
Sampson 2007[115]	36	UK	Donepezil 5 mg PO	2/21 (9.5)	Placebo tablet	5/15 (33.3)	After surgery complete	PO	Deliriu m Sympto m Intervie w	NR
Sauer 2014[116]	737	Netherla nds	Dexamethasone 1 mg/kg (100 mg maximum) single intravenous injection	52/367 (14.2)	Equivolume saline	55/370 (14.9)	During surgery	IV	CAM/C AM-ICU	100*
Shehabi 2009[117]	306	Australi a	Dexmedetomidine iv infusion 0.1–0.7 mcg/k/h	13/154 (8.4)	Morphine iv infusion 10-70mcg/kg/h	22/152 (14.5)	After surgery complete	IV	CAM-ICU	100*
Shen 2022[118]	120	China	Flurbiprofen 100mg iv infusion	7/60 (11.7)	Equivolume saline	15/60 (25)	During surgery	IV	CAM/C AM-ICU	NR
Shin 2023[119]	748	South Korea	Dexmedetomidine 1mcg/kg loading and infusion (0.1–0.5 µg/kg/h)	11/374 (2.9)	Propofol TCI 1-2mcg/ml Cet	24/374 (6.4)	During surgery	IV	CAM	0.1
Shokri 2020[120]	286	Egypt	Dexmedetomidine 0.7–1.2 µg/kg/h (max 1.4). 24h or ICU	12/144 (8.3)	Clonidine 0.5mcg/kg bolus then IV infusion	23/142 (16.2)	After surgery	IV	CAM-ICU	100*

			discharge		of 1–2 µg/kg/h		complete			
Siripoonyothai 2021[121]	75	Thailand	Ketamine 1-2 mg/kg/hr continuous infusion during CPB	10/37 (27)	Propofol 25-100 mcg/kg/min continuous infusion during CPB	18/38 (47.4)	During surgery	IV	CAM	100*
Soh 2020[122]	108	South Korea	Dexmedetomidine 0.4 mg/kg/h	2/54 (3.7)	Equivolume saline	7/54 (13)	During surgery	IV	DSM V	100*
Spies 2021[123]	277	German y	Physostigmine intravenous bolus 0.02 mg/kg, then 0.01 mg/kg/h	26/136 (19.1)	Placebo injection	20/141 (14.2)	During surgery	IV	DSM IV-TR	100
Stoppe 2023[124]	1394	German y, Canada	Selenium intravenous high-dose supplementation 2000 µg/L immediately postoperatively, 1000 µg/L each day in intensive care for a maximum of 10 days	112/697 (16.1)	Placebo injection	127/697 (18.2)	During surgery	IV	CAM-ICU	100*
Su 2016[125]	700	China	Dexmedetomidine infusion (0.1 µg/kg/h)	32/350 (9.1)	Equivolume saline	79/350 (22.6)	After surgery complete	IV	CAM-ICU	100
Subramaniam 2019[126]	120	USA	Arm 1: Dexmedetomidine 0.5 - 1mcg/kg IV during chest closure, followed by a maintenance infusion of 0.1 -1.4 mcg/kg/hour + Acetaminophen (dose not specified).<br>Arm 3: Dexmedetomidine 0.5 - 1mcg/kg IV during chest closure, followed by a maintenance infusion of 0.1 -1.4 mcg/kg/hour.<br>Arm 4: Propofol 20-100mcg/kg/min + acetaminophen (dose not specified)	Arm 1: 2/29 (6.9)<br>Arm 3: 8/30 (26.7)<br>Arm 4: 4/31 (12.9)	Propofol 20-100mcg/kg/min	9/30 (30)	After surgery complete	IV	CAM/CAM-ICU	100*
Sultan 2010[127]	203	Egypt	Arm 1: Melatonin 5mg tablet. Arm 3: Midazolam 7.5mg tablet. Arm 4: Clonidine 100mcg tablet	Arm 1: 5/53 (9.4)<br>Arm 3: 22/50 (44.0)<br>Arm 4: 19/51 (37.2)	Usual care	16/49 (32.7)	Prior to start of surgery	PO	AMT	Exclude d ICU patients
Takazawa 2023[128]	202	Japan	Minocycline 100mg PO	5/100 (5)	placebo_tablet	3/102 (2.9)	Prior to start of surgery	PO	CAM-ICU	NR
Tanaka 2017[129]	90	USA	Desflurane inhaled, PSI target 30-50	0/45 (0)	Propofol infusion, PSI target 30-50	1/45 (2.2)	During surgery	INH/IV	CAM	NR
Tang 2022[130]	120	China	Arm 1: Dexmedetomidine 0.3 µg/kg/h. Arm 3: Dexmedetomidine	Arm 1: 2/40 (5)<br>Arm 3: 3/40 (7.5)	Equivolume saline	9/40 (22.5)	During surgery	IV	CAM	NR

			0.6 μg/kg/h							
Turan 2020[131]	794	USA	Dexmedetomidine 0.1mcg/kg/h induction, 0.2 on CPB, 0.4 in ICU. Stopped by 24hours.	67/398 (16.8)	Equivolume saline	46/396 (11.6)	During surgery	IV	CAM-ICU	100*
van Norden 2021[132]	60	Germany	Dexmedetomidine 0.7 mcg.kg-1.h-1	5/28 (17.9)	Equivolume saline	14/32 (43.8)	During surgery	IV	CAM	NR
Vlisides 2021[133]	65	USA	Caffeine 200mg IV infusion over 1 hour	7/34 (20.6)	Equivolume 5% dextrose	14/31 (45.2)	After surgery complete	IV	CAM	NR
Wang 2012[134]	457	China	Haloperidol 0.5 mg intravenous bolus, then infusion at a rate of 0.1 mg/h for 12 hrs	35/229 (15.3)	Equivolume saline	53/228 (23.2)	After surgery complete	IV	CAM-ICU	100
Wang 2021[135]	70	China	Parecoxib sodium 40 mg intravenously	2/35 (5.7)	Equivolume saline	8/35 (22.9)	During surgery	IV	DRS	NR
Wang 2023[136]	652	China	Dexmedetomidine intravenous bolus 0.6 μg kg−1 then 0.4 μg.kg−1.h−1	47/326 (14.4)	Equivolume saline	51/326 (15.6)	Prior to start of surgery	IV	CAM	NR
Wang 2023[137]	80	China	40mg parecoxib iv bolus	4/40 (10)	Equivolume saline	11/40 (27.5)	During surgery	IV	CAM-ICU	100*
Wang 2024[138]	120	China	Esketamine 0.5mg/kg intravenous bolus	18/63 (28.6)	Equivolume saline	19/57 (33.3)	During surgery	IV	CAM	Excluded ICU patients
Whitlock 2015[139]	7507	Canada, China, India, USA, Colombia, Australia, Italy, Iran, Czech Republic, Greece, Spain, Brazil, Austria, Belgium, Hong	Methylprednisolone 250mg iv bolus	295/3755 (7.9)	Placebo injection	289/3752 (7.7)	During surgery	IV	CAM-ICU	100*

		Kong, Argentina, Chile, Ireland								
Wittwer 2023[140]	52	USA	Ketamine 1-2mg/kg iv bolus	0/26 (0)	Propofol 0.5-1mg/kg	1/26 (3.8)	During surgery	IV	CAM	100*
Wu 2023[141]	115	China	Dexmedetomidine intranasal 2mcg/kg	3/58 (5.2)	Equivolume saline	7/57 (12.3)	After surgery complete	IN/IV	3DCAM	Excluded ICU patients
Xiang 2022[142]	171	China	Methylprednisolone 2 mg/kg IV bolus	9/85 (10.6)	Equivolume saline	20/86 (23.3)	During surgery	IV	CAM-ICU	NR
Xie 2021[143]	160	China	Edavarone 30mg over 30 min IV infusion	12/80 (15)	Equivolume saline	25/80 (31.2)	During surgery	IV	CAM	1.9
Xie 2023[144]	240	China	PCIA Dexmedetomidine 3mcg/kg & sufentanil 3mcg/kg, total amount 150 ml, 2 ml bolus dose with a lock-out of 10 min and background infusion rate 2 ml/h.	4/120 (3.3)	Equivolume saline	12/120 (10)	After surgery complete	IV	CAM-ICU	71.3
Xin 2021[145]	60	China	Dexmedetomidine 0.5 μg/kg dexmedetomidine over 10 minutes before anesthesia induction, continuous infusion of 0.4 μg/kg/h until 30 minutes before the end of surgery	3/30 (10)	Equivolume saline	10/30 (33.3)	During surgery	IV	3DCAM	NR
Xing 2021[146]	110	China	Dexmedetomidine 0.5 mg/kg IV bolus	2/55 (3.6)	Equivolume saline	8/55 (14.5)	During surgery	IV	Chinese Expert Consensus	NR
Xu 2021[147]	821	China	40 mg Rosuvastatin PO	23/410 (5.6)	Placebo tablet	42/411 (10.2)	Prior to start of surgery	PO	CAM/CAM-ICU	100
Yang 2015[148]	80	China	Dexmedetomidine(4㎍/mL) : 0.5㎍/kg/hr infusion for 1 hour before operation is completed and 0.2-0.7㎍/kg/hr infusion continuously until 6:00am the next day	2/40 (5)	Equivolume saline	5/40 (12.5)	During surgery	IV	CAM-ICU	100
Yang 2023[149]	111	China	Quetiapine 12.5mg PO	8/57 (14)	Placebo tablet	12/54 (22.2)	During surgery	IV	CAM	1.6

Yang 2023[150]	315	China	Remimazolam (0.2–0.3 mg/kg bolus, variable infusion maintenance)	23/155 (14.8)	Propofol (1.0–1.5 mg/kg bolus, variable infusion maintenance)	19/160 (11.9)	Prior to start of surgery	PO	CAM	NR
Yoo 2024[151]	128	South Korea	Dexmedetomidine 1 mcg/kg 10 minute IV bolus, continuous infusion then 0.2 mcg/kg/h	4/64 (6.2)	Equivolume saline	10/64 (15.6)	During surgery	IV	CAM-ICU	Excluded ICU patients
Youn 2017[152]	62	South Korea	Rivastigmine 4.6mg patch	5/31 (16.1)	Usual care	14/31 (45.2)	Prior to start of surgery	Patch	CAM	NR
Yuefeng 2021[153]	60	China	Dexmedetomidine 0.6mcg/kg bolus over 15 minutes before induction, 0.2mcg/kg/h infusion until 30 min before end of surgery	1/30 (3.3)	NR	3/30 (10)	During surgery	IV	CAM	NR
Zhang 2020[154]	232	China	Dexmedetomidine intravenous 0.5 mcg/kg/h bolus, 0.3mcg/kg/h infusion	20/116 (17.2)	Equivolume saline	36/116 (31)	During surgery	IV	CAM	NR
Zhang 2023[155]	117	China	Dexmedetomidine 0.2mcg/kg/h	2/58 (3.4)	Equivolume saline	0/59 (0)	After surgery complete	IV	CAM-ICU	1.7
Zhao 2020[156]	416	China	Arm 1: Dexmedetomidine 0.02 mcg/kg/h,<br>Arm 3: Dexmedetomidine 0.04 mcg/kg/h,<br>Arm 4: Dexmedetomidine 0.08 mcg/kg/h	Arm 1: 15/108 (13.8)<br>Arm 3: 5/105 (4.7)<br>Arm 4: 5/102 (4.9)	Equivolume saline	19/101 (18.8)	During surgery	IV	CAM	NR
Zhou 2022[157]	125	China	Propofol (4.0–8.0 mg/kg–1/h−1) IV infusion	14/63 (22.2)	Sevoflurane 1-1.5%	13/62 (21)	During surgery	INH/IV	CAM	NR
Zhu 2023[158]	226	China	Dexmedetomidine 0.3 μg/kg IV bolus, followed by continuous administration of 0.2–0.7 μg/kg per hour. (MOAA/S) score. The target sedation level was <2 points (lighter sedation group) or >3 points (heavier sedation group)	26/113 (23)	Propofol 0.5–3.0 mg/ kg per hour	13/113 (11.5)	During surgery	IV	CAM	NR

IV, intravenous; PO, per oral; INH, inhaled; IT, intrathecal; IN, intranasal; ICU, intensive care unit; NR, not reported; *including patients admitted to ICU for extubation

At the request of a reviewer the proportion of participants admitted to intensive care postoperatively was also extracted. There were 58% (n=92) studies reporting the proportion admitted to intensive care after surgery, 42% did not report this information, 67% (n=62) studies reporting ICU admission did not differentiate admission to 'ICU' for extubation or monitoring only from admission to ICU for ongoing critical/severe illness. In the remaining 11% (n=17) studies which reported ICU admission rates the median (IQR) proportion was 2.9 (1.5 to 14.7)%.

Table 2. Summary of timing of first administration of intervention

Number of trials	Time of first administration	Percentage of all trials
30	On the day of surgery, prior to start of surgery	19.0
95	On the day of surgery, during surgery	60.1
30	On the day of surgery, after surgery complete	20.1

Table 3. Summary of validated delirium assessment tools used

Assessment tool	Number of trials using the tool*
CAM	79
CAM-ICU	62
DSM	15
3DCAM	9
ICDSC	5
AMT	3
DOS	2
DRS	2
NuDESC	2
4AT	1
APA	1
Delirium Symptom Interview	1
Other	1

The assessment tools reported were for the delirium outcome. Other tools e.g. CAM-S, MDAS were used adjunctively in trials where delirium severity was assess as a secondary outcome (see Supplementary file 1).
*Note the total will exceed 158 trials for instances where trials used both CAM and CAM-ICU.

References of included trials

1. Abd Ellatif SE, Mowafy SMS, Shahin MA. Ketofol versus Dexmedetomidine for preventing postoperative delirium in elderly patients undergoing intestinal obstruction surgeries: a randomized controlled study. *BMC anesthesiol* 2024;24(1):1. doi: https://dx.doi.org/10.1186/s12871-023-02378-5
2. Aizawa K-i, Kanai T, Saikawa Y, et al. A novel approach to the prevention of postoperative delirium in the elderly after gastrointestinal surgery. *SURG TODAY* 2002;32(4):310-4. doi: https://dx.doi.org/10.1007/s005950200044
3. Al Tmimi L, Van Hemelrijck J, Van de Velde M, et al. Xenon anaesthesia for patients undergoing off-pump coronary artery bypass graft surgery: a prospective randomized controlled pilot trial. *Br J Anaesth* 2015;115(4):550-9. doi: https://dx.doi.org/10.1093/bja/aev303
4. Al Tmimi L, Devroe S, Dewinter G, et al. Xenon as an Adjuvant to Propofol Anesthesia in Patients Undergoing Off-Pump Coronary Artery Bypass Graft Surgery: A Pragmatic Randomized Controlled Clinical Trial. *Anesthesia and analgesia* 2017;125(4):1118-28. doi: https://dx.doi.org/10.1213/ANE.0000000000002179 PT - Article
5. Al Tmimi L, Verbrugghe P, Van de Velde M, et al. Intraoperative xenon for prevention of delirium after on-pump cardiac surgery: a randomised, observer-blind, controlled clinical trial. *Br J Anaesth* 2020(372541) doi: https://dx.doi.org/10.1016/j.bja.2019.11.037
6. Avidan MS, Maybrier HR, Abdallah AB, et al. Intraoperative ketamine for prevention of postoperative delirium or pain after major surgery in older adults: an international, multicentre, double-blind, randomised clinical trial. *Lancet* 2017;390(10091):267-75. doi: https://dx.doi.org/10.1016/S0140-6736(17)31467-8
7. Awada HN, Steinthorsdottir KJ, Schultz NA, et al. High-dose preoperative glucocorticoid for prevention of emergence and postoperative delirium in liver resection: A double-blinded randomized clinical trial substudy. *Acta Anaesthesiol Scand* 2022;66(6):696-703. doi: https://dx.doi.org/10.1111/aas.14057
8. Azeem TMA, Yosif NE, Alansary AM, et al. Dexmedetomidine vs morphine and midazolam in the prevention and treatment of delirium after adult cardiac surgery; a randomized, double-blinded clinical trial. *Saudi J Anaesth* 2018;12(2):190-97. doi: https://dx.doi.org/10.4103/sja.SJA_303_17
9. Beloeil H, Garot M, Lebuffe G, et al. Balanced Opioid-free Anesthesia with Dexmedetomidine versus Balanced Anesthesia with Remifentanil for Major or Intermediate Noncardiac Surgery. *Anesthesiology* 2021;134(4):541-51. doi: https://dx.doi.org/10.1097/ALN.0000000000003725
10. Bielza R, Llorente J, Thuissard IJ, et al. Effect of intravenous iron on functional outcomes in hip fracture: a randomised controlled trial. *Age Ageing* 2021;50(1):127-34. doi: https://dx.doi.org/10.1093/ageing/afaa107
11. Cao S-J, Zhang Y, Zhang Y-X, et al. Delirium in older patients given propofol or sevoflurane anaesthesia for major cancer surgery: a multicentre randomised trial. *Br J Anaesth* 2023;131(2):253-65. doi: https://dx.doi.org/10.1016/j.bja.2023.04.024
12. Chen P-H, Tsuang F-Y, Lee C-T, et al. Neuroprotective effects of intraoperative dexmedetomidine versus saline infusion combined with goal-directed haemodynamic therapy for patients undergoing cranial surgery: A randomised controlled trial. *Eur J Anaesthesiol* 2021;38(12):1262-71. doi: https://dx.doi.org/10.1097/EJA.0000000000001532
13. Chitnis S, Mullane D, Brohan J, et al. Dexmedetomidine Use in Intensive Care Unit Sedation and Postoperative Recovery in Elderly Patients Post-Cardiac Surgery (DIRECT). *J Cardiothorac Vasc Anesth* 2022;36(3):880-92. doi: https://dx.doi.org/10.1053/j.jvca.2021.09.024

14. Choovongkomol C, Sinchai S, Choovongkomol K. Ao - Choovongkomol C, et al. Effect of a Single-dose Dexmedetomidine on Postoperative Delirium and Intraoperative Hemodynamic Outcomes in Elderly Hip Surgery; A Randomized Controlled Trial Dexmedetomidine for Postoperative Delirium. *Siriraj Med J* 2024;76(2):80-89. doi: https://dx.doi.org/10.33192/smj.v76i2.266653 PT - Article
15. Clemmesen CG, Lunn TH, Kristensen MT, et al. Effect of a single pre-operative 125 mg dose of methylprednisolone on postoperative delirium in hip fracture patients; a randomised, double-blind, placebo-controlled trial. *Anaesthesia* 2018;73(11):1353-60. doi: https://dx.doi.org/10.1111/anae.14406
16. Coburn M, Sanders RD, Maze M, et al. The hip fracture surgery in elderly patients (HIPELD) study to evaluate xenon anaesthesia for the prevention of postoperative delirium: a multicentre, randomized clinical trial. *Br J Anaesth* 2018;120(1):127-37. doi: https://dx.doi.org/10.1016/j.bja.2017.11.015
17. de Jonghe A, van Munster BC, Goslings JC, et al. Effect of melatonin on incidence of delirium among patients with hip fracture: a multicentre, double-blind randomized controlled trial. *CMAJ* 2014;186(14):E547-56. doi: https://dx.doi.org/10.1503/cmaj.140495
18. Deiner S, Luo X, Lin H-M, et al. Intraoperative Infusion of Dexmedetomidine for Prevention of Postoperative Delirium and Cognitive Dysfunction in Elderly Patients Undergoing Major Elective Noncardiac Surgery: A Randomized Clinical Trial. *JAMA surgery* 2017;152(8):e171505. doi: https://dx.doi.org/10.1001/jamasurg.2017.1505
19. Deng Y, Wang R, Li S, et al. Methylene blue reduces incidence of early postoperative cognitive disorders in elderly patients undergoing major non-cardiac surgery: An open-label randomized controlled clinical trial. *J Clin Anesth* 2021;68:110108. doi: https://dx.doi.org/10.1016/j.jclinane.2020.110108
20. Djaiani G, Silverton N, Fedorko L, et al. Dexmedetomidine versus Propofol Sedation Reduces Delirium after Cardiac Surgery: A Randomized Controlled Trial. *Anesthesiology* 2016;124(2):362-8. doi: https://dx.doi.org/10.1097/ALN.0000000000000951
21. Duan G-Y, Duan Z-X, Chen H, et al. Cognitive function and delirium following sevoflurane or propofol anesthesia for valve replacement surgery: A multicenter randomized controlled trial. *Kaohsiung J Med Sci* 2023;39(2):166-74. doi: https://dx.doi.org/10.1002/kjm2.12618
22. Duan G, Wu J, Xu Q, et al. Effects of remimazolam on early postoperative cognitive function in elderly patients with hip fracture. 2024;29(2):146-53. doi: 10.12092/j.issn.1009-2501.2024.02.004
23. Farrer TJ, Monk TG, McDonagh DL, et al. A prospective randomized study examining the impact of intravenous versus inhalational anesthesia on postoperative cognitive decline and delirium. *Appl Neuropsychol Adult* 2023(101584082):1-7. doi: https://dx.doi.org/10.1080/23279095.2023.2246612
24. Fazel MR, Mofidian S, Mahdian M, et al. The effect of melatonin on prevention of postoperative delirium after lower limb fracture surgery in elderly patients: a randomized double blind clinical trial. *Int j burns trauma* 2022;12(4):161-67.
25. Ford AH, Flicker L, Kelly R, et al. The Healthy Heart-Mind Trial: Randomized Controlled Trial of Melatonin for Prevention of Delirium. *J Am Geriatr Soc* 2020;68(1):112-19. doi: https://dx.doi.org/10.1111/jgs.16162
26. Gajniak D, Mendrala K, Cyzowski T, et al. Efficacy of Lidocaine Infusion in High-Risk Vascular Surgery—A Randomized, Double-Blind, Placebo-Controlled Single-Center Clinical Trial. 2023;12(6) doi: 10.3390/jcm12062312
27. Gamberini M, Bolliger D, Lurati Buse GA, et al. Rivastigmine for the prevention of postoperative delirium in elderly patients undergoing elective cardiac surgery--a randomized controlled trial. *Crit Care Med* 2009;37(5):1762-8. doi: https://dx.doi.org/10.1097/CCM.0b013e31819da780

28. Gao Y, Zhu X, Huang L, et al. Effects of dexmedetomidine on cerebral oxygen saturation and postoperative cognitive function in elderly patients undergoing minimally invasive coronary artery bypass surgery. *Clin Hemorheol Microcirc* 2020;74(4):383-89. doi: https://dx.doi.org/10.3233/CH-190590
29. Gao Y, Yu H, Wang W, et al. Effect of Dexmedetomidine on The Neuroglobin Expression in Elderly Patients With Minimally Invasive Coronary Artery Bypass Graft Surgery. *Heart Surg Forum* 2021;24(5):E776-E80. doi: https://dx.doi.org/10.1532/hsf.4073
30. Ghazaly HF, Hemaida TS, Zaher ZZ, et al. A pre-anesthetic bolus of ketamine versus dexmedetomidine for prevention of postoperative delirium in elderly patients undergoing emergency surgery: a randomized, double-blinded, placebo-controlled study. *BMC anesthesiol* 2023;23(1):407. doi: https://dx.doi.org/10.1186/s12871-023-02367-8
31. Greenberg S, Murphy GS, Avram MJ, et al. Postoperative Intravenous Acetaminophen for Craniotomy Patients: A Randomized Controlled Trial. *World Neurosurg* 2018;109(101528275):e554-e62. doi: https://dx.doi.org/10.1016/j.wneu.2017.10.021
32. Guo Y, Sun L-l, Chen Z-f, et al. [Preventive effect of dexmedetomidine on postoperative delirium in elderly patients with oral cancer]. *Shanghai Kou Qiang Yi Xue* 2015;24(2):236-9.
33. Gupta PK, Verma R, Kohli M, et al. The effect of ramelteon on postoperative delirium in elderly patients: a randomised double-blind study. 2019;13(12):UC15-UC19. doi: 10.7860/JCDR/2019/42635.13384
34. Haller G, Chan MTV, Combescure C, et al. The international ENIGMA-II substudy on postoperative cognitive disorders (ISEP). 2021;11(1):11631. doi: 10.1038/s41598-021-91014-8
35. Harmon DC, Ghori KG, Eustace NP, et al. Aprotinin decreases the incidence of cognitive deficit following CABG and cardiopulmonary bypass: A pilot randomized controlled study. *Can J Anesth* 2004;51(10):1002-09. doi: https://dx.doi.org/10.1007/BF03018488 PT - Article
36. He X, Cheng K-M, Duan Y-Q, et al. Feasibility of low-dose dexmedetomidine for prevention of postoperative delirium after intracranial operations: a pilot randomized controlled trial. *BMC Neurol* 2021;21(1):472. doi: https://dx.doi.org/10.1186/s12883-021-02506-z
37. He Y, Ding Y, Zhu J, et al. Effects of dexmedetomidine combined with sevoflurane on hemodynamics, cognitive function and delirium of elderly patients undergoing laparoscopic gastrointestinal tumor resection. 2022;12(3):396-99. doi: 10.3969/j.issn.2095-1264.2022.03.17
38. He Y, Cheng J, Qin H, et al. Effect of dexmedetomidine on perioperative neurocognitive disorders in elderly frail patients undergoing hip joint surgery. 2023;43(7):793-97. doi: 10.3760/cma.j.cn131073.20230322.00706
39. He J, Zhang X, Li C, et al. Dexmedetomidine nasal administration improves perioperative sleep quality and neurocognitive deficits in elderly patients undergoing general anesthesia. *BMC anesthesiol* 2024;24(1):42. doi: https://dx.doi.org/10.1186/s12871-024-02417-9
40. Hollinger A, Rust CA, Riegger H, et al. Ketamine vs. haloperidol for prevention of cognitive dysfunction and postoperative delirium: A phase IV multicentre randomised placebo-controlled double-blind clinical trial. *J Clin Anesth* 2021;68:110099. doi: https://dx.doi.org/10.1016/j.jclinane.2020.110099
41. Hong H, Zhang D-Z, Li M, et al. Impact of dexmedetomidine supplemented analgesia on delirium in patients recovering from orthopedic surgery: A randomized controlled trial. *BMC anesthesiol* 2021;21(1):223. doi: https://dx.doi.org/10.1186/s12871-021-01441-3
42. Hongyu X, Qingting W, Xiaoling S, et al. Penehyclidine hydrochloride on postoperatively cognitive function. *Med Hypotheses* 2019;129:109246. doi: https://dx.doi.org/10.1016/j.mehy.2019.109246
43. Hu J, Zhu M, Gao Z, et al. Dexmedetomidine for prevention of postoperative delirium in older adults undergoing oesophagectomy with total intravenous anaesthesia: A double-blind, randomised clinical trial. *Eur J Anaesthesiol* 2021;38:S9-S17. doi: https://dx.doi.org/10.1097/EJA.0000000000001382

44. Hu G, Long A, Wang J, et al. EFFECTS OF ORAL ATORVASTATIN ON INFLAMMATORY MARKERS AND POSTOPERATIVE DELIRIUM IN ELDERLY PATIENTS WITH HIP FRACTURE SURGERY. 2022;70(5):944-53. doi: 10.31925/farmacia.2022.5.21
45. Huang Q, Li Q, Qin F, et al. Repeated Preoperative Intranasal Administration of Insulin Decreases the Incidence of Postoperative Delirium in Elderly Patients Undergoing Laparoscopic Radical Gastrointestinal Surgery: A Randomized, Placebo-Controlled, Double-Blinded Clinical Study. *Am J Geriatr Psychiatry* 2021;29(12):1202-11. doi: https://dx.doi.org/10.1016/j.jagp.2021.02.043
46. Huang J-W, Yang Y-F, Gao X-S, et al. A single preoperative low-dose dexamethasone may reduce the incidence and severity of postoperative delirium in the geriatric intertrochanteric fracture patients with internal fixation surgery: an exploratory analysis of a randomized, placebo-controlled trial. *J ORTHOP SURG* 2023;18(1):441. doi: https://dx.doi.org/10.1186/s13018-023-03930-2
47. Huang Q, Shi Q, Yi X, et al. Effect of Repeated Intranasal Administration of Different Doses of Insulin on Postoperative Delirium, Serum tau and Abeta Protein in Elderly Patients Undergoing Radical Esophageal Cancer Surgery. *Neuropsychiatr dis treat* 2023;19(101240304):1017-26. doi: https://dx.doi.org/10.2147/NDT.S405426
48. Hudetz JA, Patterson KM, Iqbal Z, et al. Ketamine attenuates delirium after cardiac surgery with cardiopulmonary bypass. *J Cardiothorac Vasc Anesth* 2009;23(5):651-7. doi: https://dx.doi.org/10.1053/j.jvca.2008.12.021
49. Huet O, Gargadennec T, Oilleau J-F, et al. Prevention of post-operative delirium using an overnight infusion of dexmedetomidine in patients undergoing cardiac surgery: a pragmatic, randomized, double-blind, placebo-controlled trial. *Crit Care* 2024;28(1):64. doi: https://dx.doi.org/10.1186/s13054-024-04842-1
50. Ishii K, Makita T, Yamashita H, et al. Total intravenous anesthesia with propofol is associated with a lower rate of postoperative delirium in comparison with sevoflurane anesthesia in elderly patients. *J Clin Anesth* 2016;33:428-31. doi: https://dx.doi.org/10.1016/j.jclinane.2016.04.043
51. Jiang J-L, Zhang L, He L-L, et al. Volatile Versus Total Intravenous Anesthesia on Postoperative Delirium in Adult Patients Undergoing Cardiac Valve Surgery: A Randomized Clinical Trial. *Anesthesia and analgesia* 2023;136(1):60-69. doi: https://dx.doi.org/10.1213/ANE.0000000000006257
52. Jaiswal SJ, Vyas AD, Heisel AJ, et al. Ramelteon for Prevention of Postoperative Delirium: A Randomized Controlled Trial in Patients Undergoing Elective Pulmonary Thromboendarterectomy. *Crit Care Med* 2019;47(12):1751-58. doi: https://dx.doi.org/10.1097/CCM.0000000000004004
53. Javaherforoosh Zadeh F, Janatmakan F, Shafaeebejestan E, et al. Effect of Melatonin on Delirium After on-Pump Coronary Artery Bypass Graft Surgery: A Randomized Clinical Trial. *IRAN J MED SCI* 2021;46(2):120-27. doi: https://dx.doi.org/10.30476/ijms.2020.82860.1146
54. Javaherforooshzadeh F, Babazadeh Dezfoli A, Saki Malehi A, et al. The Efficacy of Dexmedetomidine alone or with Melatonin on Delirium after Coronary Artery Bypass Graft Surgery: A Randomized Clinical Trial. *Anesth pain med* 2023;13(4):e138317. doi: https://dx.doi.org/10.5812/aapm-138317
55. Jeon Y-G, Kim S, Park J-H, et al. Incidence of intraoperative hypotension in older patients undergoing total intravenous anesthesia by remimazolam versus propofol: A randomized controlled trial. *Medicine (Baltimore)* 2023;102(49):e36440. doi: https://dx.doi.org/10.1097/MD.0000000000036440
56. Kalisvaart KJ, de Jonghe JFM, Bogaards MJ, et al. Haloperidol prophylaxis for elderly hip-surgery patients at risk for delirium: a randomized placebo-controlled study. *J Am Geriatr Soc* 2005;53(10):1658-66. doi: https://dx.doi.org/10.1111/j.1532-5415.2005.53503.x

57. Kanamori H, Fujita Y, Joko R, et al. Effect of intraoperative systemic magnesium sulphate on postoperative Richmond Agitation-Sedation Scale score after endovascular repair of aortic aneurysm under general anesthesia: A double-blind, randomized, controlled trial. *PLoS ONE* 2023;18(2):e0281457. doi: https://dx.doi.org/10.1371/journal.pone.0281457
58. Kaneko T, Cai J, Ishikura T, et al. Prophylactic consecutive administration of haloperidol can reduce the occurrence of postoperative delirium in gastrointestinal surgery. 1999;42(3):179-84.
59. Khan BA, Perkins AJ, Campbell NL, et al. Preventing Postoperative Delirium After Major Noncardiac Thoracic Surgery-A Randomized Clinical Trial. *J Am Geriatr Soc* 2018;66(12):2289-97. doi: https://dx.doi.org/10.1111/jgs.15640
60. Kim JA, Ahn HJ, Yang M, et al. Intraoperative use of dexmedetomidine for the prevention of emergence agitation and postoperative delirium in thoracic surgery: a randomized-controlled trial. *Can J Anaesth* 2019;66(4):371-79. doi: https://dx.doi.org/10.1007/s12630-019-01299-7
61. Kinouchi M, Mihara T, Taguri M, et al. The Efficacy of Ramelteon to Prevent Postoperative Delirium After General Anesthesia in the Elderly: A Double-Blind, Randomized, Placebo-Controlled Trial. *Am J Geriatr Psychiatry* 2023;31(12):1178-89. doi: https://dx.doi.org/10.1016/j.jagp.2023.07.011
62. Kluger MT, Skarin M, Collier J, et al. Steroids to reduce the impact on delirium (STRIDE): a double-blind, randomised, placebo-controlled feasibility trial of pre-operative dexamethasone in people with hip fracture. *Anaesthesia* 2021;76(8):1031-41. doi: https://dx.doi.org/10.1111/anae.15465
63. Lai Y, Chen Q, Xiang C, et al. Comparison of the Effects of Dexmedetomidine and Lidocaine on Stress Response and Postoperative Delirium of Older Patients Undergoing Thoracoscopic Surgery: A Randomized Controlled Trial. *Clinical interventions in aging* 2023;18(101273480):1275-83. doi: https://dx.doi.org/10.2147/CIA.S419835
64. Larsen KA, Kelly SE, Stern TA, et al. Administration of olanzapine to prevent postoperative delirium in elderly joint-replacement patients: a randomized, controlled trial. *Psychosomatics* 2010;51(5):409-18. doi: https://dx.doi.org/10.1176/appi.psy.51.5.409
65. Lee C, Lee CH, Lee G, et al. The effect of the timing and dose of dexmedetomidine on postoperative delirium in elderly patients after laparoscopic major non-cardiac surgery: A double blind randomized controlled study. *J Clin Anesth* 2018;47:27-32. doi: https://dx.doi.org/10.1016/j.jclinane.2018.03.007
66. Lee H, Yang SM, Chung J, et al. Effect of Perioperative Low-Dose Dexmedetomidine on Postoperative Delirium After Living-Donor Liver Transplantation: A Randomized Controlled Trial. *Transplant Proc* 2020;52(1):239-45. doi: https://dx.doi.org/10.1016/j.transproceed.2019.11.015
67. Leung JM, Sands LP, Vaurio LE, et al. Nitrous oxide does not change the incidence of postoperative delirium or cognitive decline in elderly surgical patients. *Br J Anaesth* 2006;96(6):754-60. doi: https://dx.doi.org/10.1093/bja/ael106
68. Leung JM, Sands LP, Rico M, et al. Pilot clinical trial of gabapentin to decrease postoperative delirium in older patients. *Neurology* 2006;67(7):1251-3. doi: https://dx.doi.org/10.1212/01.wnl.0000233831.87781.a9
69. Leung JM, Sands LP, Chen N, et al. Perioperative Gabapentin Does Not Reduce Postoperative Delirium in Older Surgical Patients: A Randomized Clinical Trial. *Anesthesiology* 2017;127(4):633-44. doi: https://dx.doi.org/10.1097/ALN.0000000000001804
70. Li JZ, Li XZ, Wang XM, et al. Effects of parecoxib sodium analgesia on serum concentrations of neuron-specific enolase and S-100^b and postoperative cognitive function of elderly patients undergoing acute replacement of femoral head. *Nat Med J China* 2013;93(27):2152-54. doi: https://dx.doi.org/10.3760/cma.j.issn.0376-2491.2013.27.016 PT - Article

71. Li X, Yang J, Nie X-L, et al. Impact of dexmedetomidine on the incidence of delirium in elderly patients after cardiac surgery: A randomized controlled trial. *PLoS ONE* 2017;12(2):e0170757. doi: https://dx.doi.org/10.1371/journal.pone.0170757
72. Li CJ, Wang BJ, Mu DL, et al. Randomized clinical trial of intraoperative dexmedetomidine to prevent delirium in the elderly undergoing major non-cardiac surgery. *Br J Surg* 2020;107(2):e123-e32. doi: https://dx.doi.org/10.1002/bjs.11354
73. Li X, Wu J, Lan H, et al. Effect of Intraoperative Intravenous Lidocaine on Postoperative Delirium in Elderly Patients with Hip Fracture: a Prospective Randomized Controlled Trial. 2023;17:3749-56. doi: 10.2147/DDDT.S437599
74. Li S, Li R, Li M, et al. Dexmedetomidine administration during brain tumour resection for prevention of postoperative delirium: a randomised trial. *Br J Anaesth* 2023;130(2):e307-e16. doi: https://dx.doi.org/10.1016/j.bja.2022.10.041
75. Likhvantsev VV, Landoni G, Grebenchikov OA, et al. Perioperative Dexmedetomidine Supplement Decreases Delirium Incidence After Adult Cardiac Surgery: A Randomized, Double-Blind, Controlled Study. *J Cardiothorac Vasc Anesth* 2021;35(2):449-57. doi: https://dx.doi.org/10.1053/j.jvca.2020.02.035
76. Lin H, Du J, Tian Z, et al. Hydrogen Gas Treatment Improves Postoperative Delirium and Cognitive Dysfunction in Elderly Noncardiac Patients. *J pers med* 2022;13(1) doi: https://dx.doi.org/10.3390/jpm13010067
77. Liu X, Zhang K, Wang W, et al. Dexmedetomidine Versus Propofol Sedation Improves Sublingual Microcirculation After Cardiac Surgery: a Randomized Controlled Trial. 2016;30(6):1509-15. doi: 10.1053/j.jvca.2016.05.038
78. Liu Y, Ma L, Gao M, et al. Dexmedetomidine reduces postoperative delirium after joint replacement in elderly patients with mild cognitive impairment. *Aging Clin Exp Res* 2016;28(4):729-36. doi: https://dx.doi.org/10.1007/s40520-015-0492-3
79. Liu T, Tuo J, Wei Q, et al. Effect of Perioperative Dexmedetomidine Infusion on Postoperative Delirium in Elderly Patients Undergoing Oral and Maxillofacial Surgery: A Randomized Controlled Clinical Trial. *Int J Gen Med* 2022;15(101515487):6105-13. doi: https://dx.doi.org/10.2147/IJGM.S370237
80. Liu W, Wang Y, Chen K, et al. Effect of Intraoperative Dexmedetomidine Use on Postoperative Delirium in the Elderly After Laryngectomy: A Randomized Controlled Clinical Trial. *Drug Des Devel Ther* 2023;17(101475745):2933-41. doi: https://dx.doi.org/10.2147/DDDT.S424526
81. Liu Z, Jin Y, Wang L, et al. The Effect of Ciprofol on Postoperative Delirium in Elderly Patients Undergoing Thoracoscopic Surgery for Lung Cancer: a Prospective, Randomized, Controlled Trial. 2024;18:325-39. doi: 10.2147/DDDT.S441950
82. Long Y-Q, Xu Q-Y, Zhao W-M, et al. Dexmedetomidine Infusion Versus Placebo During Light or Deep Anesthesia on Postoperative Delirium in Older Patients Undergoing Major Noncardiac Surgery: A Pilot Randomized Factorial Trial. *Anesthesia and analgesia* 2024;138(1):161-70. doi: https://dx.doi.org/10.1213/ANE.0000000000006686
83. Lu Y, Fang P-P, Yu Y-Q, et al. Effect of Intraoperative Dexmedetomidine on Recovery of Gastrointestinal Function After Abdominal Surgery in Older Adults: A Randomized Clinical Trial. *JAMA netw open* 2021;4(10):e2128886. doi: https://dx.doi.org/10.1001/jamanetworkopen.2021.28886
84. Lu Y, Yin G, Jin C, et al. The Application Value of Esketamine and Dexmedetomidine in Preventing Postoperative Delirium and Hyperalgesia in Elderly Patients with Thoracic Anesthesia. *Altern Ther Health Med* 2023(9502013)
85. Lurati Buse GAL, Schumacher P, Seeberger E, et al. Randomized comparison of sevoflurane versus propofol to reduce perioperative myocardial ischemia in patients undergoing noncardiac surgery. *Circulation* 2012;126(23):2696-704. doi: https://dx.doi.org/10.1161/CIRCULATIONAHA.112.126144

86. Lv Y, Gu L. Dexmedetomidine potential in attenuating postoperative delirium in elderly patients after total hip joint replacement. *Rev Assoc Med Bras* 2022;68(9):1166-71. doi: https://dx.doi.org/10.1590/1806-9282.20210696
87. Ma PP, Piao MH, Wang YS, et al. Influence of dexmedetomidine and sub-anesthetic dose of ketamine on postoperative delirium in elderly orthopedic patients under total intravenous anesthesia. 2013;39(1):128-32. doi: 10.7694/jldxyxb20130129
88. Ma J, Wang F, Wang J, et al. The Effect of Low-Dose Esketamine on Postoperative Neurocognitive Dysfunction in Elderly Patients Undergoing General Anesthesia for Gastrointestinal Tumors: A Randomized Controlled Trial. *Drug Des Devel Ther* 2023;17(101475745):1945-57. doi: https://dx.doi.org/10.2147/DDDT.S406568
89. Mahrose R, ElSerwi H, Maurice A, et al. Postoperative delirium after coronary artery bypass graft surgery: dexmedetomidine infusion alone or with the addition of oral melatonin. 2021;37(1):62-68. doi: 10.1080/11101849.2021.1885956
90. Maldonado JR, Wysong A, van der Starre PJA, et al. Dexmedetomidine and the reduction of postoperative delirium after cardiac surgery. *Psychosomatics* 2009;50(3):206-17. doi: https://dx.doi.org/10.1176/appi.psy.50.3.206
91. Marcantonio ER, Palihnich K, Appleton P, et al. Pilot randomized trial of donepezil hydrochloride for delirium after hip fracture. *J Am Geriatr Soc* 2011;59(7503062):S282-8. doi: https://dx.doi.org/10.1111/j.1532-5415.2011.03691.x
92. Mardani D, Bigdelian H. Prophylaxis of dexamethasone protects patients from further post-operative delirium after cardiac surgery: A randomized trial. *J res med sci* 2013;18(2):137-43.
93. Massoudi N, Mohit B, Fathi M, et al. The impact of rivastigmine on post-surgical delirium and cognitive impairment; a randomized clinical trial. *Int J Geriatr Psychiatry* 2023;38(7):e5970. doi: https://dx.doi.org/10.1002/gps.5970
94. Massoumi G, Mansouri M, Khamesipour S. Comparison of the incidence and severity of delirium and biochemical factors after coronary artery bypass grafting with dexmedetomidine: A randomized double-blind placebo-controlled clinical trial study. *ARYA Atheroscler* 2019;15(1):14-21. doi: https://dx.doi.org/10.22122/arya.v15i1.1748
95. Mei B, Meng G, Xu G, et al. Intraoperative Sedation With Dexmedetomidine is Superior to Propofol for Elderly Patients Undergoing Hip Arthroplasty: A Prospective Randomized Controlled Study. *Clin J Pain* 2018;34(9):811-17. doi: https://dx.doi.org/10.1097/AJP.0000000000000605
96. Mei X, Zheng H-L, Li C, et al. The Effects of Propofol and Sevoflurane on Postoperative Delirium in Older Patients: A Randomized Clinical Trial Study. *J Alzheimers Dis* 2020;76(4):1627-36. doi: https://dx.doi.org/10.3233/JAD-200322
97. Mei B, Xu G, Han W, et al. The Benefit of Dexmedetomidine on Postoperative Cognitive Function Is Unrelated to the Modulation on Peripheral Inflammation: A Single-center, Prospective, Randomized Study. *Clin J Pain* 2020;36(2):88-95. doi: https://dx.doi.org/10.1097/AJP.0000000000000779
98. Mohamed SA, Rady A, Youssry M, et al. Performance of Melatonin as Prophylaxis in Geriatric Patients with Multifactorial Risk for Postoperative Delirium Development: A Randomized Comparative Study. *Turk anestezi reanim derg* 2022;50(3):178-86. doi: https://dx.doi.org/10.5152/TJAR.2022.20017
99. Mohammadi M, Ahmadi M, Khalili H, et al. Cyproheptadine for the Prevention of Postoperative Delirium: A Pilot Study. *Ann Pharmacother* 2016;50(3):180-7. doi: https://dx.doi.org/10.1177/1060028015624938
100. Momeni M, Khalifa C, Lemaire G, et al. Propofol plus low-dose dexmedetomidine infusion and postoperative delirium in older patients undergoing cardiac surgery. *Br J Anaesth* 2021;126(3):665-73. doi: https://dx.doi.org/10.1016/j.bja.2020.10.041

101. Moslemi R, Khalili H, Mohammadi M, et al. Thiamine for Prevention of Postoperative Delirium in Patients Undergoing Gastrointestinal Surgery: A Randomized Clinical Trial. *J res pharm pract* 2020;9(1):30-35. doi: https://dx.doi.org/10.4103/jrpp.JRPP_19_124
102. Mu D-L, Zhang D-Z, Wang D-X, et al. Parecoxib Supplementation to Morphine Analgesia Decreases Incidence of Delirium in Elderly Patients After Hip or Knee Replacement Surgery: A Randomized Controlled Trial. *Anesthesia and analgesia* 2017;124(6):1992-2000. doi: https://dx.doi.org/10.1213/ANE.0000000000002095
103. Nishikawa K, Nakayama M, Omote K, et al. Recovery characteristics and post-operative delirium after long-duration laparoscope-assisted surgery in elderly patients: propofol-based vs. sevoflurane-based anesthesia. *Acta Anaesthesiol Scand* 2004;48(2):162-8. doi: https://dx.doi.org/10.1111/j.0001-5172.2004.00264.x
104. Niu J-Y, Yang N, Tao Q-Y, et al. Effect of Different Administration Routes of Dexmedetomidine on Postoperative Delirium in Elderly Patients Undergoing Elective Spinal Surgery: A Prospective Randomized Double-Blinded Controlled Trial. *Anesthesia and analgesia* 2023;136(6):1075-83. doi: https://dx.doi.org/10.1213/ANE.0000000000006464
105. Oh ES, Leoutsakos J-M, Rosenberg PB, et al. Effects of Ramelteon on the Prevention of Postoperative Delirium in Older Patients Undergoing Orthopedic Surgery: The RECOVER Randomized Controlled Trial. *Am J Geriatr Psychiatry* 2021;29(1):90-100. doi: https://dx.doi.org/10.1016/j.jagp.2020.05.006
106. Papadopoulos G, Pouangare M, Papathanakos G, et al. The effect of ondansetron on postoperative delirium and cognitive function in aged orthopedic patients. 2014;80(4):444-51.
107. Park JB, Bang SH, Chee HK, et al. Efficacy and safety of dexmedetomidine for postoperative delirium in adult cardiac surgery on cardiopulmonary bypass. *Korean j thorac cardiovasc surg* 2014;47(3):249-54. doi: https://dx.doi.org/10.5090/kjtcs.2014.47.3.249
108. Prakanrattana U, Prapaitrakool S. Efficacy of risperidone for prevention of postoperative delirium in cardiac surgery. *Anaesth Intensive Care* 2007;35(5):714-9. doi: https://dx.doi.org/10.1177/0310057X0703500509
109. Preveden M, Zdravkovic R, Vickovic S, et al. Dexmedetomidine vs. propofol sedation reduces the duration of mechanical ventilation after cardiac surgery - a randomized controlled trial. *Eur Rev Med Pharmacol Sci* 2023;27(16):7644-52. doi: https://dx.doi.org/10.26355/eurrev_202308_33418
110. Qi M, Li Y, Zhang T, et al. Effect of ultrasound-guided single fascia iliaca compartment block combined with esketamine on postoperative delirium in elderly patients undergoing hip fracture surgery. 2023;43(9):1062-66. doi: 10.3760/cma.j.cn131073.20230508.00908
111. Qu JZ, Mueller A, McKay TB, et al. Nighttime dexmedetomidine for delirium prevention in non-mechanically ventilated patients after cardiac surgery (MINDDS): A single-centre, parallel-arm, randomised, placebo-controlled superiority trial. *EClinicalMedicine* 2023;56(101733727):101796. doi: https://dx.doi.org/10.1016/j.eclinm.2022.101796
112. Robinson TN, Dunn CL, Adams JC, et al. Tryptophan supplementation and postoperative delirium--a randomized controlled trial. *J Am Geriatr Soc* 2014;62(9):1764-71. doi: https://dx.doi.org/10.1111/jgs.12972
113. Royse CF, Andrews DT, Newman SN, et al. The influence of propofol or desflurane on postoperative cognitive dysfunction in patients undergoing coronary artery bypass surgery. *Anaesthesia* 2011;66(6):455-64. doi: https://dx.doi.org/10.1111/j.1365-2044.2011.06704.x
114. Sakic L, Tonkovicc D, Hrgovic Z, et al. Spinal Dexamethasone Effect on Cognitive Disorders After Hip Surgery. *Med Arh* 2023;77(1):18-23. doi: https://dx.doi.org/10.5455/medarh.2023.77.18-23

115. Sampson EL, Raven PR, Ndhlovu PN, et al. A randomized, double-blind, placebo-controlled trial of donepezil hydrochloride (Aricept) for reducing the incidence of postoperative delirium after elective total hip replacement. *Int J Geriatr Psychiatry* 2007;22(4):343-9. doi: https://dx.doi.org/10.1002/gps.1679
116. Sauer AMC, Slooter AJC, Veldhuijzen DS, et al. Intraoperative Dexamethasone and Delirium After Cardiac Surgery: A Randomized Clinical Trial. *Anesthesia and analgesia* 2014;119(5):1046-52. doi: https://dx.doi.org/10.1213/ANE.0000000000000248 PT - Article
117. Shehabi Y, Grant P, Wolfenden H, et al. Prevalence of delirium with dexmedetomidine compared with morphine based therapy after cardiac surgery: a randomized controlled trial (DEXmedetomidine COmpared to Morphine-DEXCOM Study). *Anesthesiology* 2009;111(5):1075-84. doi: https://dx.doi.org/10.1097/ALN.0b013e3181b6a783
118. Shen L, Chen JQ, Yang XL, et al. Flurbiprofen used in one-lung ventilation improves intraoperative regional cerebral oxygen saturation and reduces the incidence of postoperative delirium. *Front Psychiatry* 2022;13:889637. doi: https://dx.doi.org/10.3389/fpsyt.2022.889637 PT - Article
119. Shin H-J, Woo Nam S, Kim H, et al. Postoperative Delirium after Dexmedetomidine versus Propofol Sedation in Healthy Older Adults Undergoing Orthopedic Lower Limb Surgery with Spinal Anesthesia: A Randomized Controlled Trial. *Anesthesiology* 2023;138(2):164-71. doi: https://dx.doi.org/10.1097/ALN.0000000000004438
120. Shokri H, Ali I. A randomized control trial comparing prophylactic dexmedetomidine versus clonidine on rates and duration of delirium in older adult patients undergoing coronary artery bypass grafting. *J Clin Anesth* 2020;61:109622. doi: https://dx.doi.org/10.1016/j.jclinane.2019.09.016
121. Siripoonyothai S, Sindhvananda W. Comparison of postoperative delirium within 24 hours between ketamine and propofol infusion during cardiopulmonary bypass machine: A randomized controlled trial. *Ann Card Anaesth* 2021;24(3):294-301. doi: https://dx.doi.org/10.4103/aca.ACA_85_20
122. Soh S, Shim J-K, Song J-W, et al. Effect of dexmedetomidine on acute kidney injury after aortic surgery: a single-centre, placebo-controlled, randomised controlled trial. *Br J Anaesth* 2020(372541) doi: https://dx.doi.org/10.1016/j.bja.2019.12.036
123. Spies CD, Knaak C, Mertens M, et al. Physostigmine for prevention of postoperative delirium and long-term cognitive dysfunction in liver surgery: A double-blinded randomised controlled trial. *Eur J Anaesthesiol* 2021;38(9):943-56. doi: https://dx.doi.org/10.1097/EJA.0000000000001456
124. Stoppe C, McDonald B, Meybohm P, et al. Effect of High-Dose Selenium on Postoperative Organ Dysfunction and Mortality in Cardiac Surgery Patients: The SUSTAIN CSX Randomized Clinical Trial. *JAMA Surgery* 2023;158(3):235-44. doi: https://dx.doi.org/10.1001/jamasurg.2022.6855 PT - Article
125. Su X, Meng Z-T, Wu X-H, et al. Dexmedetomidine for prevention of delirium in elderly patients after non-cardiac surgery: a randomised, double-blind, placebo-controlled trial. *Lancet* 2016;388(10054):1893-902. doi: https://dx.doi.org/10.1016/S0140-6736(16)30580-3
126. Subramaniam B, Shankar P, Shaefi S, et al. Effect of Intravenous Acetaminophen vs Placebo Combined With Propofol or Dexmedetomidine on Postoperative Delirium Among Older Patients Following Cardiac Surgery: The DEXACET Randomized Clinical Trial. *JAMA* 2019;321(7):686-96. doi: https://dx.doi.org/10.1001/jama.2019.0234
127. Sultan SS. Assessment of role of perioperative melatonin in prevention and treatment of postoperative delirium after hip arthroplasty under spinal anesthesia in the elderly. *Saudi J Anaesth* 2010;4(3):169-73. doi: https://dx.doi.org/10.4103/1658-354X.71132

128. Takazawa T, Horiuchi T, Orihara M, et al. Prevention of Postoperative Cognitive Dysfunction by Minocycline in Elderly Patients after Total Knee Arthroplasty: A Randomized, Double-blind, Placebo-controlled Clinical Trial. *Anesthesiology* 2023;138(2):172-83. doi: https://dx.doi.org/10.1097/ALN.0000000000004439
129. Tanaka P, Goodman S, Sommer BR, et al. The effect of desflurane versus propofol anesthesia on postoperative delirium in elderly obese patients undergoing total knee replacement: A randomized, controlled, double-blinded clinical trial. *J Clin Anesth* 2017;39:17-22. doi: https://dx.doi.org/10.1016/j.jclinane.2017.03.015
130. Tang Y, Wang Y, Kong G, et al. Prevention of dexmedetomidine on postoperative delirium and early postoperative cognitive dysfunction in elderly patients undergoing hepatic lobectomy. *Zhong Nan Da Xue Xue Bao Yi Xue Ban* 2022;47(2):219-25. doi: https://dx.doi.org/10.11817/j.issn.1672-7347.2022.210280
131. Turan A, Duncan A, Leung S, et al. Dexmedetomidine for reduction of atrial fibrillation and delirium after cardiac surgery (DECADE): a randomised placebo-controlled trial. *Lancet* 2020;396(10245):177-85. doi: https://dx.doi.org/10.1016/S0140-6736(20)30631-0
132. van Norden J, Spies CD, Borchers F, et al. The effect of peri-operative dexmedetomidine on the incidence of postoperative delirium in cardiac and non-cardiac surgical patients: a randomised, double-blind placebo-controlled trial. *Anaesthesia* 2021;76(10):1342-51. doi: https://dx.doi.org/10.1111/anae.15469
133. Vlisides PE, Li D, McKinney A, et al. The Effects of Intraoperative Caffeine on Postoperative Opioid Consumption and Related Outcomes After Laparoscopic Surgery: A Randomized Controlled Trial. *Anesthesia and analgesia* 2021;133(1):233-42. doi: https://dx.doi.org/10.1213/ANE.0000000000005532
134. Wang W, Li H-L, Wang D-X, et al. Haloperidol prophylaxis decreases delirium incidence in elderly patients after noncardiac surgery: a randomized controlled trial*. *Crit Care Med* 2012;40(3):731-9. doi: https://dx.doi.org/10.1097/CCM.0b013e3182376e4f
135. Wang Y-C, Yu W-Z. [Prospective study on the effect of parecoxib sodium analgesia on pain and stress response after surgery in elderly patients with hip fracture]. *Zhongguo Gu Shang* 2021;34(7):612-6. doi: https://dx.doi.org/10.12200/j.issn.1003-0034.2021.07.005
136. Wang HB, Jia Y, Zhang CB, et al. A randomised controlled trial of dexmedetomidine for delirium in adults undergoing heart valve surgery. *Anaesthesia* 2023;78(5):571-76. doi: https://dx.doi.org/10.1111/anae.15983
137. Wang J-H, Liu T, Bai Y, et al. The effect of parecoxib sodium on postoperative delirium in elderly patients with hip arthroplasty. *Front Pharmacol* 2023;14(101548923):947982. doi: https://dx.doi.org/10.3389/fphar.2023.947982
138. Wang M, Che J, Chen L, et al. Effect of low-dose esketamine on postoperative cognitive function in elderly patients undergoing non-cardiac surgery. *Chinese J Anesth* 2024;44(1):36-40. doi: https://dx.doi.org/10.3760/cma.j.cn131073.20230823.00108 PT - Article
139. Whitlock RP, Devereaux PJ, Teoh KH, et al. Methylprednisolone in patients undergoing cardiopulmonary bypass (SIRS): a randomised, double-blind, placebo-controlled trial. *Lancet* 2015;386(10000):1243-53. doi: https://dx.doi.org/10.1016/S0140-6736(15)00273-1
140. Wittwer ED, Cerhan JH, Schroeder DR, et al. Impact of ketamine versus propofol for anesthetic induction on cognitive dysfunction, delirium, and acute kidney injury following cardiac surgery in elderly, high-risk patients. *Ann Card Anaesth* 2023;26(3):274-80. doi: https://dx.doi.org/10.4103/aca.aca_106_22
141. Wu J, Liu X, Ye C, et al. Intranasal dexmedetomidine improves postoperative sleep quality in older patients with chronic insomnia: a randomized double-blind controlled trial. *Front Pharmacol* 2023;14(101548923):1223746. doi: https://dx.doi.org/10.3389/fphar.2023.1223746

142. Xiang X-B, Chen H, Wu Y-L, et al. The Effect of Preoperative Methylprednisolone on Postoperative Delirium in Older Patients Undergoing Gastrointestinal Surgery: A Randomized, Double-Blind, Placebo-Controlled Trial. *J Gerontol A Biol Sci Med Sci* 2022;77(3):517-23. doi: https://dx.doi.org/10.1093/gerona/glab248
143. Xie H-H, Ma H-Y, Zhang S, et al. Impact of edaravone on serum CXC chemokine ligand-13 levels and perioperative neurocognitive disorders in elderly patients with hip replacement. *Chin Med J* 2021;134(13):1610-15. doi: https://dx.doi.org/10.1097/CM9.0000000000001492
144. Xie K, Chen J, Tian L, et al. Postoperative infusion of dexmedetomidine via intravenous patient-controlled analgesia for prevention of postoperative delirium in elderly patients undergoing surgery. *Aging Clin Exp Res* 2023;35(10):2137-44. doi: https://dx.doi.org/10.1007/s40520-023-02497-6
145. Xin X, Chen J, Hua W, et al. Intraoperative dexmedetomidine for prevention of postoperative delirium in elderly patients with mild cognitive impairment. *Int J Geriatr Psychiatry* 2021;36(1):143-51. doi: https://dx.doi.org/10.1002/gps.5406
146. Xing C, Yan C. Effects of dexmedetomidine on the incidence of postoperative delirium and plasma S-100β protein levels following hip surgery in the elderly population. 2021;15(3):207-11. doi: 10.6890/IJGE.202107_15(3).0004
147. Xu XQ, Luo JZ, Li XY, et al. Effects of perioperative rosuvastatin on postoperative delirium in elderly patients: a randomized, double-blind, and placebo-controlled trial. 2021;9(21):5909-20. doi: 10.12998/wjcc.v9.i21.5909
148. Yang X, Li Z, Gao C, et al. Effect of dexmedetomidine on preventing agitation and delirium after microvascular free flap surgery: a randomized, double-blind, control study. *J Oral Maxillofac Surg* 2015;73(6):1065-72. doi: https://dx.doi.org/10.1016/j.joms.2015.01.011
149. Yang YP, Ding YY, Wang YY, et al. [Effects of preoperative quetiapine on postoperative delirium and sleep quality in elderly orthopaedic patients]. *Chung Hua I Hsueh Tsa Chih* 2023;103(41):3252-57. doi: https://dx.doi.org/10.3760/cma.j.cn112137-20230719-00029
150. Yang JJ, Lei L, Qiu D, et al. Effect of Remimazolam on Postoperative Delirium in Older Adult Patients Undergoing Orthopedic Surgery: a Prospective Randomized Controlled Clinical Trial. 2023;17:143-53. doi: 10.2147/DDDT.S392569
151. Yoo SH, Jue MJ, Kim Y-H, et al. The Effect of Dexmedetomidine on the Mini-Cog Score and High-Mobility Group Box 1 Levels in Elderly Patients with Postoperative Neurocognitive Disorders Undergoing Orthopedic Surgery. *J Clin Med* 2023;12(20) doi: https://dx.doi.org/10.3390/jcm12206610
152. Youn YC, Shin H-W, Choi B-S, et al. Rivastigmine patch reduces the incidence of postoperative delirium in older patients with cognitive impairment. *Int J Geriatr Psychiatry* 2017;32(10):1079-84. doi: https://dx.doi.org/10.1002/gps.4569
153. Yuefeng N, Li Z, Jun C. Effects of dexmedetomidine on short-term prognosis of elderly patients with gastric cancer after laparoscopic radical gastrectomy. 2021;33(2):134-37. doi: 10.3760/cma.j.cn115355-20200326-00149
154. Zhang W, Wang T, Wang G, et al. Effects of Dexmedetomidine on Postoperative Delirium and Expression of IL-1beta, IL-6, and TNF-alpha in Elderly Patients After Hip Fracture Operation. *Front Pharmacol* 2020;11(101548923):678. doi: https://dx.doi.org/10.3389/fphar.2020.00678
155. Zhang ZF, Su X, Zhao Y, et al. Effect of mini-dose dexmedetomidine supplemented intravenous analgesia on sleep structure in older patients after major noncardiac surgery: A randomized trial. *Sleep Med* 2023;102:9-18. doi: https://dx.doi.org/10.1016/j.sleep.2022.12.006 PT - Article
156. Zhao W, Hu Y, Chen H, et al. The Effect and Optimal Dosage of Dexmedetomidine Plus Sufentanil for Postoperative Analgesia in Elderly Patients With Postoperative Delirium and Early Postoperative Cognitive Dysfunction: A Single-Center, Prospective, Randomized, Double-Blind, Controlled Trial. *Front neurosci* 2020;14(101478481):549516. doi: https://dx.doi.org/10.3389/fnins.2020.549516

157. Zhou Y, Li Z, Ma Y, et al. The Effect of Propofol versus Sevoflurane on Postoperative Delirium in Parkinson's Disease Patients Undergoing Deep Brain Stimulation Surgery: An Observational Study. *Brain sci* 2022;12(6) doi: https://dx.doi.org/10.3390/brainsci12060689
158. Zhu S, Liu Y, Wang X, et al. Different Sedation Strategies in Older Patients Receiving Spinal Anesthesia for Hip Surgery on Postoperative Delirium: A Randomized Clinical Trial. *Drug Des Devel Ther* 2023;17(101475745):3845-54. doi: https://dx.doi.org/10.2147/DDDT.S439543
